# Impact of sugary drink taxes on beverage calories purchased in a national fast food restaurant chain: A quasi-experimental study

**DOI:** 10.1371/journal.pmed.1004642

**Published:** 2026-04-02

**Authors:** Pasquale E. Rummo, Juan A. Echenique, Erilia Wu, Tod Mijanovich, Sunita M. Desai, Marie A. Bragg, Beth C. Weitzman, Brian Elbel

**Affiliations:** 1 Department of Population Health, New York University Grossman School of Medicine, New York, New York, United States of America; 2 Steinhardt School of Culture, Education, and Human Development, New York University, New York, New York, United States of America; 3 Marketing Department, Stern School of Business, New York University, New York, New York, United States of America; 4 Wagner Graduate School of Public Service, New York University, New York, New York, United States of America; University of Cambridge, UNITED KINGDOM OF GREAT BRITAIN AND NORTHERN IRELAND

## Abstract

**Background:**

Sugary drink taxes have been implemented in several U.S. jurisdictions, but we know little about the impact of taxes on calories purchased in restaurants. The impact may differ in restaurant (vs. non-restaurant) settings because restaurant consumers may be less likely to travel to other jurisdictions for a single meal, choose no beverage or non-taxed beverages, decrease their beverage size, or order combo meals where the drink is bundled with other items at a single price.

**Methods and findings:**

We used six years of transaction-level sales data (2015–2020) from 7,341 Taco Bell restaurant locations to estimate the association of sugary drink policies with beverage calories purchased in the drive-through setting of fast food restaurants over time. Taco Bell restaurants represents a large sample size of data from several U.S. jurisdictions across a long follow-up period, which is unique in the literature. We defined the treatment group as restaurants in five jurisdictions where taxes were ever implemented (Albany, CA; Cook County, IL; Oakland, CA; Philadelphia, PA; Seattle, WA) (*n* = 60 restaurants). We identified a group of comparison restaurants where taxes were never implemented using synthetic control methods (*n* = 60 restaurants). We used a difference-in-differences design with calendar month and restaurant fixed effects to compare changes in outcomes between groups between the baseline (3–14 months prior to tax implementation) and 3- to 24-month follow-up periods, overall and by jurisdiction. Our primary outcome measure was beverage calories per transaction, from individually-purchased beverages and combo meals (separately). In the baseline period, average beverage calories per transaction were 51.1 (SD = 8.6) in the tax group and 42.3 (SD = 7.4) in the comparison group; and 119.5 (SD = 15.3) and 115.0 (SD = 23.0) beverage calories per transaction in combo meals. Overall, we observed no association between taxes and changes in beverage calories per transaction between groups during the follow-up period, including from individual beverage items (difference-in-differences = −0.3 (95% CI [−0.8, 1.2]) and combo meals (difference-in-differences = −4.3 (95% CI [−13.5, 5.0]). We observed similar results by location, except in Oakland, CA, where customers purchased 16.8 (95% CI 19.6, 14.1) fewer beverage calories per transaction from combo meals; the association was null after conditioning on the purchase of a beverage (difference-in-differences = −1.01 [−4.93, 2.92)]). The main limitations of our study methodology include the exclusion of beverage calorie data from in-store transactions and that the majority of the restaurants in our sample were located in Cook County.

**Conclusions:**

Though we observed differences in certain jurisdictions, overall our findings suggest that sugary drink taxes may not be effective in reducing beverage calorie consumption in fast food restaurants.

## Author summary

**Why was this study done?:**

- Sugary drink taxes have been implemented in several U.S. jurisdictions, but we know little about how the taxes affect calories purchased in fast food restaurants.
- The impact may differ in restaurant (vs. non-restaurant) settings because restaurant consumers may be less likely to travel to other jurisdictions for a single meal, choose no beverage or non-taxed beverages, decrease their beverage size, or order combo meals where the drink is bundled with other items at a single price.

**What did the researchers do and find?:**

- Using six years of transaction-level sales data from 120 Taco Bell restaurant locations and a difference-in-differences design, we estimated the association of sugary drink policies with beverage calories purchased in the drive-through setting of fast food restaurants.
- Overall, we observed no association between taxes and changes in beverage calories per transaction during the follow-up period, overall and by location (Albany, CA; Cook County, IL; Oakland, CA; Philadelphia, PA; Seattle, WA).

**What do these findings mean?:**

- Overall, our findings suggest that sugary drink taxes may not be effective in reducing beverage calorie consumption in fast food restaurants, potentially because the sizes of sugary drink taxes in the U.S. are too small, sugary drink prices in restaurant settings may be inelastic, and/or other non-fiscal mechanisms.
- Our study was limited the exclusion of beverage calorie data from in-store transactions and how the majority of the restaurants in our sample were located in Cook County.

## Abstract 3

In a quasi-experimental study, Pasquale Rummo and colleagues investigate the association between sugary drink taxes and calories purchased from beverages per transaction and jurisdiction, in the drive-through setting of 7341 Taco Bell restaurants based in five USA jurisdictions.

## Introduction

Consuming sugary drinks contributes to higher total energy intake and an increased risk of obesity and heart disease [1,2], with variation by age, income, educational attainment, and race/ethnicity [3,4]. Sugary drink taxes are a policy tool designed to improve dietary behaviors and mitigate the risk of nutrition-related diseases by increasing the price of sugary drink products in food retail spaces. They are typically imposed as volume-based excise taxes (i.e., fee per ounce) [5]. In the U.S., sugary drink taxes have been implemented in several jurisdictions between 2015 and 2018, including Albany, CA, Berkeley, CA, Boulder, CO, Cook County, IL, Oakland, CA, Philadelphia, PA, San Francisco, CA, Seattle, WA, and a tax was passed Santa Cruz, CA in 2024. Current taxes range from one to two cents per ounce and apply to most calorically sweetened beverages in all locations. In Cook County and Philadelphia, non-calorically sweetened beverages are also taxed; and the tax was repealed in Cook County after 4 months of implementation.

Most research on sugary drink taxes focuses on sales that occur in non-restaurant retailers, such as supermarkets. A systematic review estimates that taxes in grocery stores are associated with a 15% decrease in sugary drink sales [5], though some studies reported no impact in Berkeley, San Francisco, Seattle, and Oakland [6–9]. More recent work corroborates the finding that sugary drink taxes curb purchases of taxed beverages in food stores [10–17], including in Albany, Berkeley, Boulder, Oakland, Philadelphia, San Francisco, and Seattle; and that taxes targeting both calorically and non-calorically sweetened beverages may be associated with greater reductions in sugary drink purchases (compared to taxing only calorically sweetened beverages) [10]. Previous work also suggests that taxes result in decreases in self-reported consumption of sugary drinks in Berkeley [18,19], but not in Philadelphia [20,21], Oakland [7], or San Francisco [19]. Those studies also suggest that reductions in self-reported intake occur primarily for soda but not other types of beverages [20,22,23]. Other work shows sugary drink taxes are associated with reduced tooth decay [24] and BMI [23,25], and improvements in perinatal health [26]. Many other countries have enacted sugary drink taxes and research suggests those taxes are linked to decreases in purchases and dietary intake of sugary drinks, though results vary by location and type of beverage [27,28]. Few studies, however, have evaluated the impact of sugary drink taxes in restaurant settings, domestically or internationally.

About one-third of Americans are estimated to consume food from fast food restaurants on a given day [29,30], where sugary drinks comprise >80% of beverages listed on menus [31]. Approximately 16% of sugary drinks consumed are purchased in fast food restaurants [32]. The impact of sugary drink taxes may differ in restaurant (vs. non-restaurant) settings because restaurant consumers may be less likely to travel to other jurisdictions for a single meal, choose no beverage or non-taxed beverages, or decrease their beverage size. Pass-through may also differ, given how consumers typically order drinks intended for consumption by one person and combo meals where the drink is bundled with other items at a single price. Previous work, including our own, has shown no significant price changes in restaurants located in Seattle, WA, Albany, CA, and Oakland, CA in response to sugary drink taxes [33–35], and, in contrast, full pass-through in cities taxing both calorically and non-calorically sweetened beverages (Cook County, Philadelphia). While taxes typically affect purchase behaviors via price changes, it is also possible that sugary drink taxes influence consumption in locations with no pass-through via a “signaling effect,” [36] whereby information (e.g., public discussion of the tax) independently leads to a reduction in sugary drink purchases.

To our knowledge, only one study has assessed the impact of sugary drink taxes on restaurant purchases, which found that the tax was associated with reduced purchases of taxed juice drinks in fast food restaurants but not other types of taxed beverages in Philadelphia [37]. Despite the strengths of the study, the impact of the tax was only examined in one jurisdiction, so we do not know how associations might have varied by jurisdiction, which is important because tax rate, taxable beverages, and other unmeasured factors (e.g., cultural norms) vary by jurisdiction. Additionally, the relatively small sample size and short period of data collection are key limitations. In the U.K., one restaurant chain increased the price of sugary drinks by 10 pence per ounce and researchers observed a large decrease in sales, but the price increase was paired with several non-fiscal components (e.g., beverage menu redesign) that make it difficult for comparison [38].

To address the gaps in the literature, we used six years of transaction-level sales data from 120 Taco Bell restaurants in five jurisdictions where sugary drink tax policies were implemented and in jurisdictions where such taxes were never implemented. Using a quasi-experimental design, we estimated the association between sugary drink taxes and calories purchased from beverages per transaction by jurisdiction. We hypothesized that sugary drink taxes would be associated with a decrease in calories purchased from beverages per transaction in all jurisdictions.

## Methods

### Data sources

Taco Bell provided data from 7,439 Taco Bell restaurants in the U.S. that were open at any time during the study period, representing 6.86 billion transactions from 2015 to 2020. Taco Bell is the third most popular fast food restaurant chain in the U.S., where half of the population eats at least once a month [39,40]. Taco Bell doubled its international footprint in the past six years, with ~1,100 locations outside of the U.S. and plans to triple the count in the next five years [41]. Transaction data included the date, time, and location of the purchase; how the order was placed (drive-through, eat-in, takeout); the name, quantity, and price of items purchased, including customizations; whether a beverage was ordered as an individual item or part of a combo meal; and the type and size of beverages in drive-through transactions. Menu items were standard and did not vary across restaurant locations, excepting slight variation during production. It was not possible to assign calories to self-serve beverage purchases with respect to eat-in and takeout transactions, so our analyses only included drive-through transactions (70% of all purchases).

Between 2015 and 2018, several jurisdictions implemented sugary drink taxes, including Albany, CA, Berkeley, CA, Oakland, CA, San Francisco, CA, Boulder, CO, Cook County, IL, Philadelphia, PA, and Seattle, WA. We used information from the Center for Science in the Public Interest to identify dates of sugary drink tax implementation, complemented by legislation source text, peer-reviewed literature, news articles, and direct communication with Taco Bell (Table 1). We required that restaurants in the analytic sample have monthly transaction data in the baseline period, which we defined as the three to 14 months prior to the date of location-specific sugary drink tax implementation. Based on this criterion, our final sample includes 47 restaurants in Cook County, IL—where the tax was repealed after four months—one restaurant in Albany, CA, and four restaurants in each of the following cities: Oakland, CA, Philadelphia, PA, and Seattle, WA; and only 5 restaurant locations were excluded from the follow-up period at 24-months due to closures or a lack of data availability (Table A in S1 Appendix). We observed no restaurant data in Berkeley, CA and incomplete data during the baseline period in Boulder, CO and San Francisco, CA.

**Table 1:** Distribution of restaurants in soda tax group and comparison group by location.

Location	Date of tax implementation	Tax rate	Average list price^a^ of beverages ($/oz), individual items	Average sales price^b^ of beverages ($/oz), individual items	Average list price^a^ of combo meals ($/oz)	Average sales price^b^ of combo meals ($/oz)	Taxable beverages	*N*
Philadelphia, PA	Jan 2017	$0.015/oz	$0.0912	$0.0973	$6.61	$5.35	SSB + NCS	4 (7%)
Albany, CA	Apr 2017	$0.01/oz	$0.0886	$0.0951	$6.32	$5.04	SSB	1 (2%)
Oakland, CA	Jul 2017	$0.01/oz	$0.0917	$0.1071	$6.58	$5.18	SSB	4 (7%)
Seattle, WA	Jan 2018	$0.0175/oz	$0.0900	$0.0933	$6.32	$5.39	SSB	4 (7%)
Cook County, IL	Aug – Dec 2017^c^	$0.01/oz	$0.0859	$0.0916	$6.14	$5.19	SSB + NCS	47 (78%)

^a^List price defined as the average item price listed on the menu.

^b^Sales price defined as the average item price on the menu, weighted by sales of that item.

^c^Tax repealed.

SSB, sugar-sweetened beverage; NCS, non-caloric sweetened beverage.

### Comparison group

Restaurants were eligible for inclusion in a comparison group if they were located in a jurisdiction with no sugary drink tax policy (*n* = 7,341) before or during the study period. We also required that restaurants in both groups have monthly transaction data during the baseline period, and complete data on all matching variables (*n* = 5,775).

To construct a comparison unit for each restaurant in the sugary drink tax groups, we used synthetic control methods. This approach generates a comparison unit for each restaurant in the tax groups using a weighted average of restaurants in cities without sugary drink taxes. The weights are calculated based on the outcome variable and an array of restaurant- and community-level characteristics in the baseline period (Table B in S1 Appendix) [42–44]. Each synthetic comparison unit in our study, therefore, represents a weighted average of data from multiple potential comparison restaurants. We excluded data from two months before the location-specific date of sugary drink tax implementation from the weighting process, to account for anticipatory effects.

To reduce computational burden, we restricted the pool of potential comparison restaurants to 100 per restaurant in the sugary drink tax group. This was based on the nearest Mahalanobis distance of the restaurant characteristics vector with the nearest match. We then constructed the synthetic control unit for each of the 60 restaurants in the sugary drink tax group using the tidysynth package in R. The weights of comparison units added up to one for each of the restaurants in the tax group (see eMethods in S1 Appendix for code). After weighting, the restaurant- and community-level characteristics of treatment and comparison groups were similar (Table B in S1 Appendix). Out of the 100 donors that were selected for each treatment unit, 5.85 donors had a non-trivial weight per treated unit on average. See Fig A in S1 Appendix for a map of the geographic locations of comparison restaurants, overall and by jurisdiction.

### Outcomes

Our two primary outcomes were the average number of calories per transaction for beverages purchased with a combo meal and without a combo meal. Secondary outcomes included total calories, sugar (grams) from beverages, count of beverages, and the percentage of transactions with a beverage. We used MenuStat, a nutrition database of foods and beverages served by national chain restaurants [45], to assign calories to unique menu items (*N* = 4,712). We matched over 95% of total purchased items every quarter using both automated and manual matching of menu items and nutrition information (see eMethods in S1 Appendix for methods).

### Statistical analysis

To account for potential restaurant-level differences in the timing of tax implementation, we excluded data from two months before and two months after the effective date of location-specific sugary drink taxes. We used a two-way fixed effects regression model to compare the differences in beverage calories purchased per transaction before and after sugary drink tax implementation between tax and comparison restaurants by jurisdiction [46]. The use of a two-way fixed effects model as a complement to our matching approach eliminates observed baseline differences between groups [47–49], a potential weakness of synthetic control methods.

The model is as follows


$$\begin{array}{c} Y_{it}=\beta_{\text{0}}+\beta_{\text{1}}{tax}_{i}+\beta_{\text{2}}month_{tj}+\sum_{j=-\text{1}\text{4}}^{\text{2}\text{4}}\beta_{\text{3}j}({tax}_{i}\times month_{tj})+\alpha_{k}+\tau_{i}+\in_{i} \end{array}$$

where i denotes a treated or untreated restaurant; t denotes relative month from implementation, with 0 being the implementation month, presented as a vector of factor variables; k denotes calendar months 1–12 for January to December; Y denotes the outcome; the vector $\alpha_{k}$ denotes calendar month fixed effects to control for seasonality; the vector $\tau_{i}$ denotes restaurant fixed effects to control for time-invariant confounding at the restaurant-level; and $\in_{i}$ denotes the restaurant-level random error term. We implemented robust standard errors to account for heteroskedasticity. We also used a bootstrap method with 100 reiterations to construct 95% confidence intervals for estimates derived from the one restaurant unit located in Albany, CA.

We stratified by jurisdiction to capture potential location-specific differences in the impact of taxes, including tax rate, taxable beverages (e.g., inclusion of non-caloric sweeteners), and other unmeasured factors (e.g., cultural norms). Transaction data were aggregated as restaurant-month observations. Given how covariances were parameterized as factors in our models, we accounted for the covariances between months in our estimation of confidence intervals using the R library ‘marginaleffects’ and the ‘hypotheses’ function. To assess parallel trends in the pre-period, we performed equivalence tests using the R library ‘EquiTrends’ [50]. Our primary (“unconditional”) model included all transactions whether or not they included a beverage, which captured any change in beverage size as well as decisions not to purchase a beverage.

We assessed heterogeneity by time of day, with strata defined as late night (00:00–03:59), breakfast (04:00–10:59), lunch (11:00–13:59), afternoon (14:00–16:59), dinner (17:00–20:59), and evening (21:00–23:59), to test the hypothesis that sugary drink taxes may be more effective during “off-peak” hours. We estimated the impact of sugary drink taxes in the two-year follow-up period in all analyses as the mean of the relative month difference-in-differences estimates during the 3–24 months following tax implementation (or 3–4 months for Cook County, IL). Although the tax was repealed in Cook County after 4 months of implementation, we aggregated those data with the other restaurants in the full sample because we observed pass-through in restaurants in Cook County after the repeal of the tax, which would theoretically induce changes in purchasing behaviors in the absence of taxes. Because of potential item-level variation in the amount of pass-through, all analyses were also run separately for individual beverage items and combo meals. We also estimated (“conditional”) models using only transactions that included beverages, including separate models for individual beverage items, where the denominator included beverage calories from individual beverage items, but excluded calories from beverages in combo meals; and for combo meals, where the denominator included beverage calories from all combo meals that include at least one beverage, but excluded calories from individual beverage items. Our rationale for estimating conditional models was to capture potential compensatory behaviors, such as shifting from combo meals with beverages to combo meals without beverages between time points (~76% of combo meals include a combo meal beverage).

To assess the degree to which results were sensitive to our approach to matching, the specification of the baseline and follow-up periods, differential behavioral responses to tax repeal and the extent of pass-through, and differential loss-to-follow-up, we re-estimated our models using (1) no matching, (2) a 6-month baseline period, (3) no data from only one month before and one month after the date of location-specific sugary drink taxes, (4) only 2 months of follow-up data across the full sample, (5) a stratified sample of Cook County restaurants and all other restaurants, (6) a stratified sample of locations with and without pass-through, and (7) only restaurants that were open for at least 12, 18, and 24 months following sugary drink tax implementation. We also re-estimated models with only tax-eligible beverages, which included 95%, 94%, 86%, 80%, and 80% of beverages in Philadelphia, Cook County, Oakland, Albany, and Seattle, respectively; the higher percentage of tax-eligible beverages in Philadelphia and Cook is due to the inclusion of artificially sweetened beverages. We conducted analyses using R version 4.3.1. Analyses were planned in January 2024 and we made the following changes in response to peer-review comments: re-estimating analyses with a different wash-out period, a different number of months of follow-up data, and only tax-eligible beverages; and stratifying the sample by Cook County restaurants versus all other restaurants, and locations with and without pass-through. Reporting followed the STROBE reporting guidelines for cohort studies [51]. Human subjects were not involved in the design and conduct of this research (see S1 STROBE Checklist). This study was deemed exempted from review by the NYU Grossman School of Medicine Office of Science and Research Institutional Review Board because the use of de-identified transaction data is considered minimal risk.

## Results

**Fig 1:**
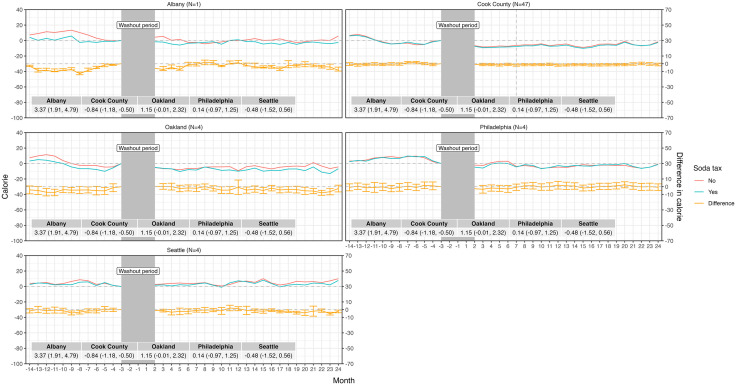
Difference-in-differences model estimates of beverage calories purchased per transaction after implementation of taxes, individual items, conditional,^a^ by location. NOTE: The vertical dashed line represents the date of repeal for Cook County, IL. ^a^Conditional on a transaction including a beverage item.

**Fig 2:**
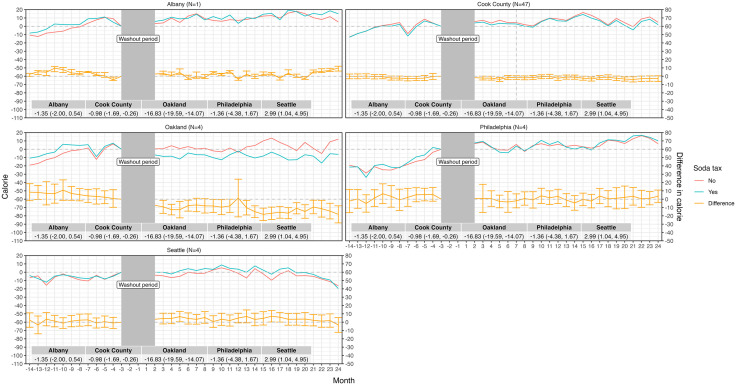
Difference-in-differences model estimates of beverage calories purchased per transaction after implementation of taxes, combo meals, unconditional,^a^ by location. NOTE: The vertical dashed line represents the date of repeal for Cook County, IL. ^a^Unconditional on a transaction including a beverage item.

The final sample size across all analyses was 120 unique Taco Bell restaurants and 4,368 restaurant-month observations, with 60 restaurants in the sugary drink tax group and 60 synthetic control units (Table A in S1 Appendix). A little over half of transactions included a beverage in the baseline period; and about a third of beverages were purchased individually versus in combo meals (Table C.1 in S1 Appendix), with little variation by time of day and tax group (Figs D.1 and D.2 in S1 Appendix). Total calories, beverage sugar, and beverage count per transaction were similar between groups at baseline (Tables C.2, C.3, and C.4 in S1 Appendix). In the baseline period, average calories of individual beverage items per transaction were 51.1 (SD = 8.6) in the tax group and 42.3 (SD = 7.4) in the comparison group; and 119.5 (SD = 15.3) and 115.0 (SD = 23.0) beverage calories per transaction in combo meals (Table 2). Trends in beverage calories purchased were parallel between tax and comparison groups in all jurisdictions in the baseline period, including beverage calories purchased from individual beverage items and combo meals (Figs 1 and 2).

**Table 2:** Descriptive statistics of the beverage calories purchased, single items and combo meals, overall and by location.

	*Beverage calories, single items*				*Beverage calories, combo meals*			
	Tax group		Comparison group		Tax group		Comparison group	
	Baseline	Follow-up	Baseline	Follow-up	Baseline	Follow-up	Baseline	Follow-up
	Mean (SD)		Mean (SD)		Mean (SD)		Mean (SD)	
***Unconditional*** ^a^								
*Philadelphia, PA (n = 4)*	60.5 (12.4)	52.8 (11.5)	48.4 (9.3)	40.4 (8.1)	125.3 (21.8)	146.5 (19.3)	128.8 (25.1)	151.1 (28.3)
*Albany, CA (n = 1)*	57.5 (5.0)	51.9 (2.5)	40.9 (2.9)	37.5 (1.7)	94.5 (8.0)	103.5 (3.8)	57.5 (5.0)	51.9 (2.5)
*Oakland, CA (n = 4)*	55.0 (5.9)	47.7 (30.0)	45.7 (5.7)	40.0 (4.6)	126.7 (10.5)	135.2 (7.7)	139.9 (19.6)	131.5 (13.4)
*Seattle, WA (n = 4)*	50.9 (3.6)	51.6 (3.0)	44.8 (4.2)	46.1 (4.6)	118.6 (7.7)	123.1 (6.6)	97.2 (6.9)	105.1 (7.3)
*Cook County, IL (n = 47)*	51.4 (8.3)	45.9 (6.8)	42.7 (7.2)	36.5 (6.6)	118.0 (15.4)	125.6 (13.7)	112.6 (21.9)	120.2 (20.4)
***Conditional*** ^b^								
*Philadelphia, PA (n = 4)*	270.1 (30.4)	265.6 (26.9)	257.8 (20.4)	255.8 (24.5)	358.6 (14.9)	362.9 (17.1)	365.9 (17.2)	367.4 (31.4)
*Albany, CA (n = 1)*	236.5 (5.2)	233.9 (3.5)	219.5 (9.0)	208.2 (7.2)	318.2 (5.4)	317.0 (6.1)	318.0 (7.1)	312.5 (6.2)
*Oakland, CA (n = 4)*	259.2 (11.6)	252.8 (7.6)	245.3 (21.2)	231.4 (16.7)	356.8 (12.4)	356.4 (11.0)	366.2 (25.6)	365.4 (19.6)
*Seattle, WA (n = 4)*	236.8 (6.3)	243.8 (7.2)	221.4 (24.8)	232.0 (24.7)	336.7 (10.3)	343.3 (11.3)	321.2 (18.6)	332.3 (20.5)
*Cook County, IL (n = 47)*	252.6 (21.0)	248.8 (18.3)	248.1 (25.6)	241.4 (23.5)	343.0 (17.6)	342.3 (17.5)	335.5 (23.5)	334.6 (22.1)

^a^Conditional on a transaction including a beverage item.

^b^Unconditional on a transaction including a beverage item.

SD, standard deviation.

In all jurisdictions, we observed little to no impact of beverage tax policies on beverage calories purchased per transaction between groups in the 3- to 24-month follow-up period from individual beverage items or combo meals, unconditional on the inclusion of beverages in transactions (Table 3). One exception was Oakland, CA, where customers ordered on average 16.8 (95% CI −19.6, −14.1) fewer beverage calories per transaction from combo meals, relative to the comparison group. Results were similar for all estimates conditional on the inclusion of beverages in transactions (Figs B and C in S1 Appendix), and also by month in the follow-up period (Table G in S1 Appendix). However, the impact on beverage calories from combo meals in Oakland, CA was null after conditioning (difference-in-differences = −1.01 (95% CI [−4.93, 2.92]), suggesting that customers shifted to purchasing combo meals without beverages in the follow-up period, with no meaningful change in beverage calories purchased from combo meals (only). We also observed wide variation in estimates by restaurant within jurisdiction (Figs G.1 and G.2 and Table H in S1 Appendix).

**Table 3:** Difference-in-differences estimates of calories purchased per transaction after implementation of sugary drink taxes, overall and by location, average effect across months 3–24.

	*Beverage calories, single items*			*Beverage calories, combo meals*		
	Difference, soda tax restaurants	Difference, comparison restaurants	Difference-in-differences	Difference, soda tax restaurants	Difference, comparison restaurants	Difference-in-differences
	β (95% CI)	β (95% CI)	β (95% CI)	β (95% CI)	β (95% CI)	β (95% CI)
***Unconditional*** ^a^						
Philadelphia, PA (*n* = 4)	−8.50 (−9.36, −7.64)	−8.65 (−9.35, −7.95)	0.14 (−0.97, 1.25)	21.88 (19.33, 24.42)	23.23 (21.59, 24.87)	−1.36 (−4.38, 1.67)
Albany, CA (*n* = 1)^c^	−3.61 (−3.61, −3.61)	−6.99 (−8.40, −5.52)	3.37 (1.91, 4.79)	9.99 (9.99, 9.99)	11.34 (9.45, 11.99)	−1.35 (−2.00, 0.54)
Oakland, CA (*n* = 4)	−5.92 (−6.93, −4.90)	−7.07 (−7.63, −6.51)	1.15 (−0.01, 2.32)	−7.89 (−10.14, −5.64)	8.94 (7.34, 10.54)	−16.83 (−19.59, −14.07)
Seattle, WA (*n* = 4)	0.25 (−0.73, 1.22)	0.73 (0.37, 1.09)	−0.48 (−1.52, 0.56)	6.02 (4.67, 7.38)	3.03 (1.62, 4.44)	2.99 (1.04, 4.95)
Cook County, IL (*n* = 47)^d^	−6.36 (−6.65, −6.08)	−5.52 (−5.70, −5.35)	−0.84 (−1.18, −0.50)	7.14 (6.51, 7.77)	8.11 (7.78, 8.44)	−0.98 (−1.69, −0.26)
***Conditional*** ^b^						
Philadelphia, PA (*n* = 4)	−1.65 (−3.89, 0.59)	−6.88 (−8.66, −5.09)	5.22 (2.36, 8.09)	5.10 (1.00, 9.20)	2.91 (1.49, 4.34)	2.19 (−2.15, 6.53)
Albany, CA (*n* = 1)^c^	−11.38 (−11.38, −11.38)	−3.70 (−12.74, 2.60)	−7.69 (−13.98, 1.36)	−3.69 (−3.69, −3.69)	−2.68 (−4.42, 1.89)	−1.01 (−5.58, 0.73)
Oakland, CA (*n* = 4)	−13.58 (−16.77, −10.39)	−5.46 (−6.68, −4.24)	−8.12 (−11.53, −4.71)	−0.02 (−3.42, 3.39)	0.99 (−0.97, 2.94)	−1.01 (−4.93, 2.92)
Seattle, WA (*n* = 4)	2.84 (−0.35, 6.04)	7.84 (6.23, 9.45)	−5.00 (−8.57, −1.42)	3.95 (2.08, 5.81)	4.53 (3.49, 5.57)	−0.58 (−2.72, 1.55)
Cook County, IL (*n* = 47)^d^	−6.57 (−7.58, −5.56)	−3.80 (−4.35, −3.24)	−2.77 (−3.93, −1.62)	−1.26 (−1.93, −0.58)	0.08 (−0.26, 0.41)	−1.33 (−2.09, −0.58)

^a^Conditional on a transaction including a beverage item.

^b^Unconditional on a transaction including a beverage item.

^c^The absence of a confidence interval for Albany, CA estimates is due to the sample size of one restaurant.

^d^Estimates correspond to an average effect across 5 months, given how the tax was repealed in Cook County, IL.

CI, confidence interval.

In the overall sample, we observed no impact of beverage tax policies on secondary outcomes between groups over time (Table D in S1 Appendix), with no meaningful difference in the count of beverages per transaction or the percentage of transactions with a beverage, regardless of pass-through; and little to no variation by time of day (Figs F.1 and F.2 in S1 Appendix). In Philadelphia, however, we observed fewer total calories purchased in the tax group in the follow-up period (difference-in-differences = −28.2 (95% CI [−40.86, −15.51]), with a larger impact during breakfast hours (difference-in-differences = −72.6 (95% CI [−90.2, −55.0]). In Oakland, we also observed fewer total calories purchased in the tax group (difference-in-differences = −45.7 (95% CI [−56.0, −33.4]), but, in contrast, no impact during breakfast hours (difference-in-differences = 0.7 (95% CI [−14.3, 15.7]).

Our results were robust to the length of time restaurants were open following sugary drink tax implementation (Table F and Figs G.1 and G.2 in S1 Appendix), indicating that restaurant closures did not drive our findings. Our results were also robust to using an unmatched sample of restaurants, using a 6-month baseline period, using a ±1-month washout period, restricting to 2 months of follow-up data across the full sample, stratifying the sample by Cook County restaurants and all other restaurants, and stratifying the sample by locations with and without pass-through (Table G in S1 Appendix). We also did not observe meaningful differences in estimates in models with only tax-eligible beverages.

## Discussion

We found that the implementation of sugary drink taxes in fast food restaurants in five U.S. jurisdictions was not associated with meaningful changes in beverage calories purchased over time. In contrast, a systematic review reported that sugary drink taxes are associated with a ~15% reduction in sugary drink sales in supermarkets and other types of non-restaurant retailers,^5^ with similar findings in more recent studies [10–16]. One jurisdiction in our analysis where we did find an impact of the tax was Oakland, CA, where customers ordered ~14% fewer beverage calories per transaction from combo meals, and ~7% fewer *total* calories per transaction, relative to the comparison group. We similarly observed that customers ordered ~32% fewer total calories in restaurants in Philadelphia, PA, but no difference in beverage calories, suggesting that customers in Oakland and Philadelphia may have changed their non-beverage purchases after tax implementation. We also observed variation in the magnitude of association by restaurant within locations, potentially due to differences in baseline consumption, social norms, or other unmeasured population-level characteristics.

In a separate study using the same data, we found that the extent of pass-through varied by jurisdiction, with complete pass-through on individual beverage items in Philadelphia, PA, and Cook County, IL, where taxes applied to both calorically and non-calorically sweetened beverages, and partial pass-through to combo meal prices in Cook County (and not in Philadelphia). However, price changes did not lead to purchase changes in Philadelphia and Cook County. There was no pass-through on any purchases with beverages in Oakland, CA, Seattle, WA, and Albany, CA in that study. In contrast, we observed changes in total calories purchased in Oakland and Philadelphia, which suggests that customers in Philadelphia may have changed their purchases of non-beverage items in response to tax implementation. In both cities, a “signaling effect” may have also shifted customers to purchase meals with lower overall calories, which is corroborated by prior work showing how the implementation of the beverage tax in Philadelphia was associated with decreases in the perceived healthfulness of taxed beverages two years later [52]. In Cook County, the public discussion of tax repeal may also have independently lead to a reduction in sugary drink purchases, above and beyond changes in price.

Our results are consistent with the one other study assessing the impact of sugary drink taxes on sales from restaurant purchases. Grummon and colleagues followed a cohort of adults in Philadelphia and Baltimore who submitted all food and beverage receipts before and after the implementation of the Philadelphia tax in 2016, and reported that the tax did not lead to a reduction in purchases of taxed beverages in fast food restaurants, except for juice drinks with added sugars (which are not sold in Taco Bell restaurants), and no reduction in purchases of taxed beverages of any type in sit-down restaurants [37]. The study only examined one jurisdiction’s tax policy, with no reporting on any type of calories purchased, but the longitudinal panel was a key strength. As with this previous study, our results suggest that sugary drink taxes in the range of one to two cents per ounce may not be effective in fast food restaurants, in contrast to their impact in food stores (e.g., supermarkets, pharmacies). Our results also did not meaningfully differ in locations where we observed pass-through, regardless of tax repeal. This provides additional evidence to suggest that the sizes of sugary drink taxes in the U.S. are too small to effect meaningful changes to restaurant purchases, potentially because restaurant consumers may be less likely to travel to other jurisdictions for a single meal, choose no beverage or non-taxed beverages, or decrease their beverage size. We cannot observe whether larger taxes would be more effective using real-world data, but simulation studies using hypothetical restaurant data may be instructive in the future.

A key limitation of our study was the exclusion of beverage calorie data from in-store transactions (~30% of the sample), due to the nature of self-serve fountain beverage purchases. We do not know the demographic characteristics of the person(s) who made the transactions, but it is possible that those who order food in a drive-through setting differ from those who order food in-store (e.g., age differences) [53]. In the absence of randomization, our quasi-experimental approach was rigorous and accounted for secular trends over time; and our synthetic control matching approach allowed for closely parallel trends in the baseline period. That said, it is possible that unobserved characteristics of comparison restaurants were correlated with sugary drink tax implementation, and thus our approach to matching may have been biased. In addition, our data may not be generalizable to all restaurant settings or jurisdictions, including locations with sugary tax policies where we lacked sufficient or any transaction data. The majority of the restaurants in our sample (47 out of 60) were located in Cook County, where the tax was repealed after 4 months of implementation; pass-through was observed for the two years after implementation of the tax in Cook County, however, and the 13 restaurants located outside of Cook County represent hundreds of thousands of transactions. Overall, we maintain that our limitations are an acceptable trade-off of analyzing a detailed set of objective data from millions of transactions in multiple jurisdictions.

The findings from our quasi-experimental study are valuable in that we used millions of transactions across five jurisdictions and demonstrate that these sugary drink taxes were not effective in the fast food restaurants in our sample. The changes in total calories purchased in some jurisdictions, however, provide evidence that customers may change their non-beverage purchases after tax implementation, potentially due to location-specific variation in the nature of price changes. Though we observed variation in estimates by fast food restaurant within jurisdictions, the impact of sugary drink taxes in aggregate, in combination with our evaluation of price changes, suggests that the sizes of sugary drink taxes in the U.S. are too small to change beverage calories purchased in fast food restaurants.

## Supporting information

S1 Appendix **Table A.** Number of restaurants open in tax group and comparison group, by location and months open after tax implementation. **Table B.** Restaurant-level and community-level characteristics of restaurants used for synthetic control matching, by location, unweighted and weighted. SD = standard deviation. **Table C.1.** Descriptive statistics of secondary outcomes, transaction percentages, overall and by location and time of day. ^a^Unconditional on a transaction including a beverage item. ^b^Conditional on a transaction including a beverage item. **Table C.2.** Descriptive statistics of secondary outcomes, total calories, overall and by location and time of day. SD = standard deviation. ^a^Unconditional on a transaction including a beverage item. ^b^Conditional on a transaction including a beverage item. **Table C.3.** Descriptive statistics of secondary outcomes, beverage sugar (g), overall and by location and time of day. SD = standard deviation. ^a^Unconditional on a transaction including a beverage item. ^b^Conditional on a transaction including a beverage item. **Table C.4.** Descriptive statistics of secondary outcomes, beverage count, overall and by location and time of day. SD = standard deviation. ^a^Unconditional on a transaction including a beverage item. ^b^Conditional on a transaction including a beverage item. **Table D.** Difference-in-differences model-based estimates of purchase outcomes after tax implementation, by location and time of day, average effect across months 3–24. CI = confidence interval. ^a^Unconditional on a transaction including a beverage item. ^b^Conditional on a transaction including a beverage item. ^c^We used a bootstrap method with 100 reiterations to construct 95% confidence intervals for estimates derived from the one restaurant unit located in Albany, CA. ^d^Estimates correspond to an average effect across 5 months, given how the tax was repealed in Cook County, IL. **Table E.** Difference-in-differences model estimates of beverage calories purchased per transaction, by months open after tax implementation. CI = confidence interval. ^a^Unconditional on a transaction including a beverage item. ^b^We used a bootstrap method with 100 reiterations to construct 95% confidence intervals for estimates derived from the one restaurant unit located in Albany, CA. ^c^Conditional on a transaction including a beverage item. **Table F.** Difference-in-differences model-based estimates of beverage calories purchased per transaction after tax implementation, 6-month baseline period. CI = confidence interval. ^a^Unconditional on a transaction including a beverage item. ^b^Conditional on a transaction including a beverage item. **Table G.** Difference-in-differences model-based estimates of beverage calories purchased per transaction after tax implementation, by location and month. CI = confidence interval. ^a^The absence of a confidence interval for Albany, CA estimates is due to the sample size of one restaurant. ^b^Unconditional on a transaction including a beverage item. ^c^Conditional on a transaction including a beverage item. **Table H.** Difference-in-differences model-based estimates of beverage calories purchased per transaction after tax implementation, by restaurant location. **Fig A.** Map of the locations of the restaurants in the tax group and comparison group in the final sample. **Fig B.** Difference-in-differences model estimates^a^ of beverage calories purchased per transaction, by location, individual items, conditional^b^. ^a^Estimates in legends represent difference-in-differences estimates with 95% confidence intervals. ^a^Conditional on a transaction including a beverage item. **Fig C.** Difference-in-differences model estimates^a^ of beverage calories purchased per transaction, by location, combo meals, conditional^b^. ^a^Estimates in legends represent difference-in-differences estimates with 95% confidence intervals. ^b^Conditional on a transaction including a beverage item. **Fig D.1.** Percentage sales of individual items by location and time of day, tax group and comparison group. **Fig D.2.** Percentage sales of combo meals, by location and time of day, tax group and comparison group. **Fig E.1.** Difference-in-differences model estimates of beverage calories purchased per transaction, individual items, by location and time of day, unconditional. ^a^Unconditional on a transaction including a beverage item. **Fig E.2.** Difference-in-differences model estimates of beverage calories purchased per transaction, combo meals, by location and time of day, unconditional. ^a^Unconditional on a transaction including a beverage item. **Fig F.1.** Difference-in-differences model estimates of beverage calories purchased from individual items per transaction after tax implementation, by months open after tax implementation, unconditional. ^a^Unconditional on a transaction including a beverage item. **Fig F.2.** Difference-in-differences model estimates of beverage calories purchased from combo meals per transaction after tax implementation, by months open after soda tax implementation, unconditional. ^a^Unconditional on a transaction including a beverage item. **Fig G.1.** Difference-in-differences model estimates of beverage calories purchased per transaction after tax implementation, by restaurant, individual items, unconditional. ^a^Unconditional on a transaction including a beverage item. **Fig G.2.** Difference-in-differences model estimates of beverage calories purchased per transaction after tax implementation, by restaurant, combo meals, conditional, unconditional. ^a^Unconditional on a transaction including a beverage item. eMethods. Supplementary description of matching procedures and statistical analyses.
(DOCX)

S1 STROBE Checklist Checklist of items that should be included in reports of observational studies. This checklist is reproduced from the STROBE Statement (Strengthening the Reporting of Observational Studies in Epidemiology) and is licensed under the Creative Commons Attribution 4.0 International (CC BY 4.0). Source: https://www.strobe-statement.org/.
(DOCX)
