# Supplementary material for: Impact of sugary drink taxes on beverage calories purchased in a national fast food restaurant chain: A quasi-experimental study
Source: PLoS Med. 2026 Apr 2;23(4):e1004642. doi: 10.1371/journal.pmed.1004642 (PMC13046137; doi:10.1371/journal.pmed.1004642)
Supplement: S1 Appendix — Table A. Number of restaurants open in tax group and comparison group, by location and months open after tax implementation. Table B. Restaurant-level and community-level characteristics of restaurants used for synthetic control matching, by location, unweighted and weighted. SD = standard deviation. Table C.1. Descriptive statistics of secondary outcomes, transaction percentages, overall and by location and time of day. aUnconditional on a transaction including a beverage item. bConditional on a transaction including a beverage item. Table C.2. Descriptive statistics of secondary outcomes, total calories, overall and by location and time of day. SD = standard deviation. aUnconditional on a transaction including a beverage item. bConditional on a transaction including a beverage item. Table C.3. Descriptive statistics of secondary outcomes, beverage sugar (g), overall and by location and time of day. SD = standard deviation. aUnconditional on a transaction including a beverage item. bConditional on a transaction including a beverage item. Table C.4. Descriptive statistics of secondary outcomes, beverage count, overall and by location and time of day. SD = standard deviation. aUnconditional on a transaction including a beverage item. bConditional on a transaction including a beverage item. Table D. Difference-in-differences model-based estimates of purchase outcomes after tax implementation, by location and time of day, average effect across months 3–24. CI = confidence interval. aUnconditional on a transaction including a beverage item. bConditional on a transaction including a beverage item. cWe used a bootstrap method with 100 reiterations to construct 95% confidence intervals for estimates derived from the one restaurant unit located in Albany, CA. dEstimates correspond to an average effect across 5 months, given how the tax was repealed in Cook County, IL. Table E. Difference-in-differences model estimates of beverage calories purchased per transaction, by month [file pmed.1004642.s001.docx]

**Table A.** Number of restaurants open in tax group and comparison group, by location and months open after tax implementation

|  | Restaurants, open ever (n), **unmatched** | Restaurants with data available in baseline period (n), **matched**, **open ever** | Restaurants with data available in baseline period (n (%)), **matched, open ≥12 months** | Restaurants with data available in baseline period (n (%)), **matched**, **open ≥18 months** | Restaurants with data available in baseline period (n (%)), **matched**, **open ≥24 months** |
| --- | --- | --- | --- | --- | --- |
|  |  |  |  |  |  |
| Philadelphia, PA | 6 | 4 | 4 | 4 | 3 |
| Comparison restaurants | 7,013 | 4 | 4 | 4 | 3 |
|  |  |  |  |  |  |
| Albany, CA | 1 | 1 | 1 | 1 | 1 |
| Comparison restaurants | 7,057 | 1 | 1 | 1 | 1 |
|  |  |  |  |  |  |
| Oakland, CA | 4 | 4 | 4 | 4 | 4 |
| Comparison restaurants | 7,100 | 4 | 4 | 4 | 4 |
|  |  |  |  |  |  |
| Seattle, WA | 9 | 4 | 2 | 2 | 2 |
| Comparison restaurants | 7,233 | 4 | 2 | 2 | 2 |
|  |  |  |  |  |  |
| Cook County, IL | 73 | 47 | 45 | 43 | 42 |
| Comparison restaurants | 7,123 | 47 | 45 | 43 | 42 |

**Table B.** Restaurant-level and community-level characteristics of restaurants used for synthetic control matching, by location, unweighted and weighted

|  |  | *Weighted* | *Unweighted* |
| --- | --- | --- | --- |
|  | Tax group | Comparison group | |
|  | Mean (SD) | Mean (SD) | Mean (SD) |
| **Philadelphia, PA (n=4)** |  |  |  |
| *Restaurant-level characteristics* |  |  |  |
| Company owned (%) | 0.00 | 0.00 | 0.02 |
| Joint brand (%) | 50.0 (70.7) | 34.1 (48.2) | 81.0 (24.0) |
| Mean calorie per transaction, baseline (mean(SD)) | 1332 (232) | 1352 (202) | 1352 (63) |
| Number of transactions, baseline (mean (SD)) | 11022 (3024) | 9632 (3709) | 11296 (860) |
| Mean sales price, combo meal, baseline (mean(SD)) | 5.35 (0.18) | 5.01 (0.21) | 4.97 (0.12) |
| Mean sales price, single beverage, baseline (mean(SD)) | 0.10 (0.01) | 0.09 (0.00) | 0.09 (0.00) |
| Mean spending, baseline, (mean(SD)) | 7.17 (1.22) | 6.81 (0.58) | 6.68 (0.27) |
|  |  |  |  |
| *Community-level characteristics* |  |  |  |
| Population count (mean (SD)) | 4903 (2021) | 4590 (1472) | 4647 (513) |
| Less than 18 years of age (%) | 14.4 (9.2) | 23.6 (1.7) | 21.2 (1.3) |
| Greater than 65 years of age (%) | 16.7 (8.7) | 14.4 (3.8) | 16.3 (1.7) |
| Male (%) | 50.9 (6.0) | 48.6 (2.0) | 49.1 (1.0) |
| Asian population (%) | 12.9 (9.3) | 1.8 (1.2) | 4.8 (1.6) |
| Black population (%) | 9.3 (14.6) | 21.4 (23.2) | 8.8 (1.7) |
| Hispanic population (%) | 8.7 (3.4) | 20.9 (15.4) | 14.3 (2.9) |
| White population (%) | 65.4 (23.7) | 53.1 (34.4) | 68.4 (6.1) |
| Median household income (mean (SD)) | 61503 (11161) | 55883 (12594) | 66342 (3615) |
| Income per capita (mean (SD)) | 34234 (4968) | 28421 (5424) | 34088 (1130) |
| No high school degree (%) | 57.3 (7.0) | 55.7 (2.7) | 58.5 (2.3) |
| College degree or higher (%) | 26.5 (8.0) | 15.3 (4.3) | 21.5 (2.4) |
|  |  |  |  |
| **Albany, CA (n=1)** |  |  |  |
| *Restaurant-level characteristics* |  |  |  |
| Company owned (%) | - | - | - |
| Joint brand (%) | - | - | - |
| Mean calorie per transaction, baseline (mean(SD)) | 1242 | 1242 | 1310 |
| Number of transactions, baseline (mean (SD)) | 7370 | 10695 | 10768 |
| Mean sales price, combo meal, baseline (mean(SD)) | 5.04 | 4.85 | 4.89 |
| Mean sales price, single beverage, baseline (mean(SD)) | 0.10 | 0.09 | 0.09 |
| Mean spending, baseline, (mean(SD)) | 6.37 | 6.15 | 6.41 |
|  |  |  |  |
| *Community-level characteristics* |  |  |  |
| Population count (mean (SD)) | 3275 | 3483 | 4514 |
| Less than 18 years of age (%) | 23.0 | 21.3 | 22.3 |
| Greater than 65 years of age (%) | 14.3 | 12.7 | 14.3 |
| Male (%) | 49.3 | 48.0 | 49.1 |
| Asian population (%) | 42.2 | 25.0 | 19.0 |
| Black population (%) | 6.4 | 10.5 | 8.2 |
| Hispanic population (%) | 7.8 | 11.1 | 15.0 |
| White population (%) | 30.8 | 48.6 | 52.2 |
| Median household income (mean (SD)) | 115000 | 94713 | 85980 |
| Income per capita (mean (SD)) | 55197 | 44387 | 41915 |
| No high school degree (%) | 77.8 | 64.6 | 65.7 |
| College degree or higher (%) | 48.1 | 31.0 | 30.2 |
|  |  |  |  |
| **Oakland, CA (n=4)** |  |  |  |
| *Restaurant-level characteristics* |  |  |  |
| Company owned (%) | 0.00 (0.00) | 3.19 (5.52) | 0.03 (0.02) |
| Joint brand (%) | - | - | - |
| Mean calorie per transaction, baseline (mean(SD)) | 1366 (89) | 1366 (83) | 1363 (66) |
| Number of transactions, baseline (mean (SD)) | 12818 (1701) | 11634 (313) | 11881 (446) |
| Mean sales price, combo meal, baseline (mean(SD)) | 5.18 (0.35) | 4.89 (0.23) | 4.96 (0.19) |
| Mean sales price, single beverage, baseline (mean(SD)) | 0.11 (0.01) | 0.09 (0.00) | 0.09 (0.00) |
| Mean spending, baseline, (mean(SD)) | 7.32 (0.12) | 6.66 (0.08) | 6.68 (0.07) |
|  |  |  |  |
| *Community-level characteristics* |  |  |  |
| Population count (mean (SD)) | 5244 (1515) | 5068 (461) | 4564 (403) |
| Less than 18 years of age (%) | 20.8 (10.3) | 20.9 (1.7) | 21.6 (2.5) |
| Greater than 65 years of age (%) | 10.5 (3.0) | 13.7 (2.4) | 15.3 (1.1) |
| Male (%) | 46.8 (4.1) | 50.5 (2.6) | 48.9 (0.7) |
| Asian population (%) | 12.9 (8.8) | 5.9 (3.4) | 5.5 (0.8) |
| Black population (%) | 28.3 (8.8) | 17.5 (6.5) | 11.8 (1.6) |
| Hispanic population (%) | 35.7 (21.9) | 31.1 (10.7) | 21.6 (6.0) |
| White population (%) | 17.1 (17.2) | 41.3 (6.2) | 56.4 (5.0) |
| Median household income (mean (SD)) | 69597 (14805) | 62016 (11874) | 65217 (2770) |
| Income per capita (mean (SD)) | 35200 (18547) | 29131 (5199) | 33346 (3430) |
| No high school degree (%) | 66.7 (5.1) | 60.2 (0.3) | 60.3 (1.2) |
| College degree or higher (%) | 24.6 (16.7) | 16.2 (5.0) | 20.8 (3.8) |
|  |  |  |  |
| **Seattle, WA (n=4)** |  |  |  |
| *Restaurant-level characteristics* |  |  |  |
| Company owned (%) | 66.67 (57.74) | 13.42 (21.90) | 0.37 (0.33) |
| Joint brand (%) | - | - | - |
| Mean calorie per transaction, baseline (mean(SD)) | 1292 (81) | 1292 (79) | 1303 (70) |
| Number of transactions, baseline (mean (SD)) | 10483 (1540) | 12660 (1239) | 11824 (612) |
| Mean sales price, combo meal, baseline (mean(SD)) | 5.39 (0.14) | 5.12 (0.10) | 5.13 (0.08) |
| Mean sales price, single beverage, baseline (mean(SD)) | 0.09 (0.00) | 0.09 (0.00) | 0.09 (0.00) |
| Mean spending, baseline, (mean(SD)) | 6.87 (0.24) | 6.73 (0.30) | 6.68 (0.14) |
|  |  |  |  |
| *Community-level characteristics* |  |  |  |
| Population count (mean (SD)) | 4534 (1007) | 4487 (875) | 4632 (276) |
| Less than 18 years of age (%) | 14.4 (7.6) | 21.4 (3.0) | 20.1 (1.9) |
| Greater than 65 years of age (%) | 12.5 (6.6) | 15.3 (4.7) | 15.4 (2.1) |
| Male (%) | 50.2 (5.2) | 49.4 (2.6) | 49.3 (0.3) |
| Asian population (%) | 18.8 (5.2) | 8.9 (5.9) | 6.2 (1.8) |
| Black population (%) | 5.7 (6.3) | 11.0 (4.8) | 8.7 (2.0) |
| Hispanic population (%) | 7.3 (4.3) | 13.2 (3.7) | 13.1 (0.6) |
| White population (%) | 60.9 (6.0) | 62.0 (11.4) | 66.9 (3.8) |
| Median household income (mean (SD)) | 101863 (3673) | 74009 (14530) | 76469 (2993) |
| Income per capita (mean (SD)) | 62721 (17349) | 40499 (7142) | 40981 (3177) |
| No high school degree (%) | 77.4 (7.5) | 64.5 (6.4) | 63.4 (2.3) |
| College degree or higher (%) | 49.1 (20.2) | 29.0 (9.7) | 28.5 (4.3) |
|  |  |  |  |
| **Cook County, IL (n=47)** |  |  |  |
| *Restaurant-level characteristics* |  |  |  |
| Company owned (%) | 0.00 (0.00) | 2.20 (6.87) | 0.04 (0.03) |
| Joint brand (%) | 16.7 (40.8) | 31.9 (37.1) | 74.5 (14.7) |
| Mean calorie per transaction, baseline (mean(SD)) | 1239 (122) | 1242 (113) | 1312 (67) |
| Number of transactions, baseline (mean (SD)) | 9008 (1978) | 10233 (1147) | 10882 (507) |
| Mean sales price, combo meal, baseline (mean(SD)) | 5.19 (0.27) | 5.04 (0.22) | 5.05 (0.14) |
| Mean sales price, single beverage, baseline (mean(SD)) | 0.09 (0.01) | 0.09 (0.00) | 0.09 (0.00) |
| Mean spending, baseline, (mean(SD)) | 6.43 (0.42) | 6.36 (0.37) | 6.52 (0.10) |
|  |  |  |  |
| *Community-level characteristics* |  |  |  |
| Population count (mean (SD)) | 4634 (1621) | 4593 (849) | 4525 (361) |
| Less than 18 years of age (%) | 21.6 (7.7) | 22.0 (3.1) | 22.1 (1.7) |
| Greater than 65 years of age (%) | 17.4 (9.6) | 15.4 (3.2) | 16.1 (2.0) |
| Male (%) | 48.5 (4.3) | 49.1 (2.4) | 49.1 (0.8) |
| Asian population (%) | 7.7 (8.2) | 5.9 (4.9) | 4.2 (1.4) |
| Black population (%) | 14.3 (24.4) | 17.9 (19.2) | 10.4 (6.3) |
| Hispanic population (%) | 26.3 (24.7) | 17.0 (10.9) | 18.0 (6.7) |
| White population (%) | 49.4 (25.4) | 55.6 (17.9) | 63.5 (8.0) |
| Median household income (mean (SD)) | 75363 (30157) | 73488 (20924) | 69688 (9478) |
| Income per capita (mean (SD)) | 38420 (17579) | 36467 (10362) | 35455 (5575) |
| No high school degree (%) | 62.1 (10.9) | 62.2 (5.2) | 60.5 (2.9) |
| College degree or higher (%) | 24.9 (13.5) | 23.4 (8.3) | 22.3 (4.4) |

SD=standard deviation

**Table C.1.** Descriptive statistics of secondary outcomes, transaction percentages, overall and by location and time of day

|  | *% of transactions with a beverage* | | | | *% of beverage transactions from individual items (vs. combo meals)* | | | |
| --- | --- | --- | --- | --- | --- | --- | --- | --- |
|  | Tax group | | Comparison group | | Tax group | | Comparison group | |
|  | Baseline | Follow-up | Baseline | Follow-up | Baseline | Follow-up | Baseline | Follow-up |
|  | % | % | % | % | % | % | % | % |
| ***UNCONDITIONAL***^b^ |  |  |  |  |  |  |  |  |
| ***Philadelphia, PA (n=4)*** |  |  |  |  |  |  |  |  |
| Total | 53.0 | 55.9 | 56.2 | 58.9 | 32.6 | 25.8 | 37.1 | 31.2 |
| *Time of day* |  |  |  |  |  |  |  |  |
| Late night | 48.0 | 52.0 | 48.4 | 53.4 | 40.8 | 30.1 | 41.7 | 33.9 |
| Breakfast | 45.7 | 46.0 | 49.3 | 51.8 | 50.6 | 44.1 | 50.8 | 43.6 |
| Lunch | 57.5 | 60.7 | 61.2 | 64.0 | 37.7 | 30.1 | 42.9 | 35.2 |
| Afternoon | 57.7 | 57.8 | 58.7 | 60.1 | 10.4 | 10.3 | 19.7 | 19.4 |
| Dinner | 50.0 | 53.7 | 52.5 | 56.0 | 38.7 | 28.8 | 40.4 | 31.6 |
| Evening | 49.8 | 54.3 | 52.7 | 55.9 | 40.3 | 30.2 | 40.0 | 32.4 |
|  |  |  |  |  |  |  |  |  |
| ***Albany, CA (n=1)*** |  |  |  |  |  |  |  |  |
| Total | 46.2 | 48.8 | 52.8 | 54.2 | 38.6 | 35.2 | 45.1 | 39.7 |
| *Time of day* |  |  |  |  |  |  |  |  |
| Late night | 39.1 | 44.0 | 49.9 | 51.8 | 36.0 | 38.7 | 46.9 | 40.0 |
| Breakfast | 40.6 | 44.2 | 47.0 | 48.1 | 54.6 | 46.4 | 60.4 | 52.8 |
| Lunch | 53.0 | 55.2 | 59.4 | 60.3 | 48.9 | 42.0 | 51.6 | 44.9 |
| Afternoon | 53.2 | 51.4 | 56.6 | 56.5 | 16.8 | 19.2 | 23.1 | 23.1 |
| Dinner | 42.0 | 46.2 | 48.8 | 51.0 | 42.4 | 36.7 | 48.9 | 41.3 |
| Evening | 38.9 | 45.3 | 49.4 | 52.0 | 38.3 | 35.8 | 48.2 | 41.5 |
|  |  |  |  |  |  |  |  |  |
|  |  |  |  |  |  |  |  |  |
| ***Oakland, CA (n=4)*** |  |  |  |  |  |  |  |  |
| Total | 55.9 | 52.8 | 55.6 | 55.8 | 31.4 | 30.5 | 35.4 | 31.1 |
| *Time of day* |  |  |  |  |  |  |  |  |
| Late night | 54.8 | 52.6 | 53.3 | 54.4 | 34.7 | 33.4 | 37.8 | 33.3 |
| Breakfast | 47.2 | 48.0 | 46.6 | 47.6 | 49.7 | 44.5 | 54.9 | 49.0 |
| Lunch | 59.3 | 56.3 | 60.3 | 60.1 | 39.4 | 35.8 | 42.5 | 36.9 |
| Afternoon | 61.4 | 53.7 | 59.7 | 57.0 | 11.4 | 14.9 | 17.1 | 16.9 |
| Dinner | 54.3 | 51.3 | 53.4 | 54.8 | 34.8 | 32.2 | 38.0 | 31.5 |
| Evening | 54.6 | 52.8 | 53.8 | 55.2 | 33.5 | 32.0 | 36.6 | 31.7 |
|  |  |  |  |  |  |  |  |  |
|  |  |  |  |  |  |  |  |  |
| ***Seattle, WA (n=4)*** |  |  |  |  |  |  |  |  |
| Total | 50.3 | 51.0 | 55.6 | 56.0 | 38.7 | 37.1 | 36.2 | 35.3 |
| *Time of day* |  |  |  |  |  |  |  |  |
| Late night | 46.4 | 48.8 | 53.0 | 54.1 | 40.7 | 38.2 | 38.9 | 37.7 |
| Breakfast | 43.2 | 42.8 | 47.5 | 48.3 | 51.6 | 49.1 | 51.4 | 48.7 |
| Lunch | 54.8 | 54.0 | 61.5 | 61.1 | 42.7 | 41.6 | 41.2 | 40.3 |
| Afternoon | 52.6 | 52.8 | 57.3 | 56.4 | 22.7 | 22.1 | 20.4 | 21.0 |
| Dinner | 49.9 | 51.5 | 53.6 | 54.3 | 40.9 | 39.4 | 36.7 | 35.6 |
| Evening | 48.5 | 50.0 | 53.7 | 54.3 | 40.6 | 39.0 | 38.0 | 37.1 |
|  |  |  |  |  |  |  |  |  |
| ***Cook County, IL (n=47)*** |  |  |  |  |  |  |  |  |
| Total | 50.7 | 50.4 | 54.0 | 54.1 | 32.7 | 28.5 | 35.5 | 32.0 |
| *Time of day* |  |  |  |  |  |  |  |  |
| Late night | 48.1 | 49.5 | 50.0 | 51.7 | 34.3 | 29.9 | 37.2 | 31.6 |
| Breakfast | 43.8 | 44.2 | 46.1 | 47.0 | 48.8 | 42.7 | 53.9 | 48.4 |
| Lunch | 55.9 | 55.7 | 59.7 | 60.0 | 36.8 | 31.8 | 40.4 | 36.2 |
| Afternoon | 54.1 | 51.8 | 57.3 | 54.9 | 17.1 | 16.0 | 18.5 | 19.1 |
| Dinner | 47.6 | 47.8 | 51.2 | 51.8 | 35.6 | 30.0 | 37.7 | 32.3 |
| Evening | 48.7 | 49.1 | 51.3 | 52.2 | 35.1 | 30.0 | 36.8 | 31.8 |
|  |  |  |  |  |  |  |  |  |
|  |  |  |  |  |  |  |  |  |
| ***CONDITIONAL***^b^ |  |  |  |  |  |  |  |  |
| ***Philadelphia, PA (n=4)*** |  |  |  |  |  |  |  |  |
| Total | - | - | - | - | - | - | - | - |
| *Time of day* |  |  |  |  |  |  |  |  |
| Late night | - | - | - | - | - | - | - | - |
| Breakfast | - | - | - | - | - | - | - | - |
| Lunch | - | - | - | - | - | - | - | - |
| Afternoon | - | - | - | - | - | - | - | - |
| Dinner | - | - | - | - | - | - | - | - |
| Evening | - | - | - | - | - | - | - | - |
|  |  |  |  |  |  |  |  |  |
| ***Albany, CA (n=1)*** |  |  |  |  |  |  |  |  |
| Total | - | - | - | - | - | - | - | - |
| *Time of day* |  |  |  |  |  |  |  |  |
| Late night | - | - | - | - | - | - | - | - |
| Breakfast | - | - | - | - | - | - | - | - |
| Lunch | - | - | - | - | - | - | - | - |
| Afternoon | - | - | - | - | - | - | - | - |
| Dinner | - | - | - | - | - | - | - | - |
| Evening | - | - | - | - | - | - | - | - |
|  |  |  |  |  |  |  |  |  |
|  |  |  |  |  |  |  |  |  |
| ***Oakland, CA (n=4)*** |  |  |  |  |  |  |  |  |
| Total | - | - | - | - | - | - | - | - |
| *Time of day* |  |  |  |  |  |  |  |  |
| Late night | - | - | - | - | - | - | - | - |
| Breakfast | - | - | - | - | - | - | - | - |
| Lunch | - | - | - | - | - | - | - | - |
| Afternoon | - | - | - | - | - | - | - | - |
| Dinner | - | - | - | - | - | - | - | - |
| Evening | - | - | - | - | - | - | - | - |
|  |  |  |  |  |  |  |  |  |
|  |  |  |  |  |  |  |  |  |
| ***Seattle, WA (n=4)*** |  |  |  |  |  |  |  |  |
| Total | - | - | - | - | - | - | - | - |
| *Time of day* |  |  |  |  |  |  |  |  |
| Late night | - | - | - | - | - | - | - | - |
| Breakfast | - | - | - | - | - | - | - | - |
| Lunch | - | - | - | - | - | - | - | - |
| Afternoon | - | - | - | - | - | - | - | - |
| Dinner | - | - | - | - | - | - | - | - |
| Evening | - | - | - | - | - | - | - | - |
|  |  |  |  |  |  |  |  |  |
| ***Cook County, IL (n=47)*** |  |  |  |  |  |  |  |  |
| Total | - | - | - | - | - | - | - | - |
| *Time of day* |  |  |  |  |  |  |  |  |
| Late night | - | - | - | - | - | - | - | - |
| Breakfast | - | - | - | - | - | - | - | - |
| Lunch | - | - | - | - | - | - | - | - |
| Afternoon | - | - | - | - | - | - | - | - |
| Dinner | - | - | - | - | - | - | - | - |
| Evening | - | - | - | - | - | - | - | - |

^a^Unconditional on a transaction including a beverage item.

^b^Conditional on a transaction including a beverage item.

**Table C.2.** Descriptive statistics of secondary outcomes, total calories, overall and by location and time of day

|  | *Total calories,  single items* | | | | *Total calories,  combo meals* | | | |
| --- | --- | --- | --- | --- | --- | --- | --- | --- |
|  | Tax group | | Comparison group | | Tax group | | Comparison group | |
|  | Baseline | Follow-up | Baseline | Follow-up | Baseline | Follow-up | Baseline | Follow-up |
|  | Mean (SD) | | Mean (SD) | | Mean (SD) | | Mean (SD) | |
| ***UNCONDITIONAL***^a^ |  |  |  |  |  |  |  |  |
| ***Philadelphia, PA (n=4)*** |  |  |  |  |  |  |  |  |
| Total | 793.46 (110.26) | 682.5 (99.74) | 837.96 (173.32) | 750.42 (131.86) | 547.13 (181.13) | 623.6 (168.97) | 531.3 (112.84) | 608.03 (107.47) |
| *Time of day* |  |  |  |  |  |  |  |  |
| Late night | 999.22 (105.74) | 884.19 (146.91) | 994.8 (88.97) | 884.03 (146.3) | 518.58 (183.65) | 618.96 (179.95) | 492.9 (138.31) | 587.55 (169.42) |
| Breakfast | 594.48 (90.53) | 466.42 (91.08) | 670.43 (102.27) | 572.03 (87.94) | 272.89 (76.42) | 295.91 (83.04) | 313.75 (103.5) | 367.93 (106.43) |
| Lunch | 681.28 (95.29) | 583.42 (96.94) | 754.83 (141.89) | 659.5 (116.32) | 530.41 (161.51) | 594.96 (161.05) | 512.44 (113.2) | 589 (103.11) |
| Afternoon | 694.66 (104.98) | 602.41 (108.39) | 765.75 (163.83) | 685.41 (124.31) | 573.1 (190.99) | 640.73 (169.13) | 526.84 (105.8) | 597.21 (112.45) |
| Dinner | 877.24 (132.35) | 764.82 (112.4) | 979.8 (197.37) | 879.14 (147.93) | 601.28 (216.51) | 684.66 (200.33) | 598.73 (149.75) | 688.49 (154.38) |
| Evening | 918.69 (107.3) | 797.67 (87.01) | 940.02 (140.46) | 849.07 (108.52) | 550.19 (212.02) | 644.17 (193.34) | 561.86 (120.76) | 643.45 (114.61) |
|  |  |  |  |  |  |  |  |  |
| ***Albany, CA (n=1)*** |  |  |  |  |  |  |  |  |
| Total | 859.54 (37.35) | 812.47 (20.69) | 844.81 (47.38) | 778.31 (20.25) | 376.65 (70.65) | 446.45 (56.56) | 391.66 (73.82) | 449.43 (61.03) |
| *Time of day* |  |  |  |  |  |  |  |  |
| Late night | 1001.67 (91.97) | 988.39 (163.45) | 922.71 (59.09) | 849.92 (35.36) | 405.71 (100.79) | 423.91 (85.37) | 415.71 (88.92) | 479.05 (66.01) |
| Breakfast | 558.28 (44.4) | 513.29 (33.25) | 604.28 (47.57) | 552.88 (20) | 208.49 (33.69) | 271.64 (40.69) | 220.68 (22.41) | 263.79 (24.81) |
| Lunch | 781.9 (38.71) | 729.37 (21.07) | 766.21 (51.1) | 704.04 (22.24) | 389.65 (78.8) | 462.49 (56.72) | 402.65 (73.88) | 457.15 (56.31) |
| Afternoon | 795.64 (49.16) | 753.96 (34.42) | 792.84 (38.74) | 735.47 (25.91) | 399.4 (79.48) | 450.04 (59.38) | 411.08 (75.34) | 452.33 (61.31) |
| Dinner | 1012.44 (42.26) | 960.62 (33.68) | 1018.19 (52.91) | 941.46 (24.99) | 411.97 (80.53) | 497.18 (73.58) | 439.92 (87.78) | 509.5 (78.45) |
| Evening | 1030.65 (40.21) | 963.39 (76.18) | 941.61 (64.43) | 872.18 (28.71) | 400.06 (72.48) | 470.24 (63.27) | 417.98 (80.59) | 483.71 (72.02) |
|  |  |  |  |  |  |  |  |  |
|  |  |  |  |  |  |  |  |  |
| ***Oakland, CA (n=4)*** |  |  |  |  |  |  |  |  |
| Total | 749.98 (78.17) | 729.08 (60.66) | 804.48 (55.65) | 753.16 (31.87) | 604.9 (104.13) | 593.94 (81.57) | 555.86 (73.55) | 602.11 (76.48) |
| *Time of day* |  |  |  |  |  |  |  |  |
| Late night | 887.09 (167.98) | 852.96 (124.67) | 887.65 (58.57) | 844.72 (44.59) | 620.29 (107.48) | 609.8 (98.93) | 563.81 (75.91) | 604.81 (83.76) |
| Breakfast | 551 (41.02) | 527.95 (33.17) | 589.71 (40.05) | 557.79 (28.26) | 314.17 (58.17) | 347.84 (53.94) | 276.95 (32.94) | 317.47 (37.28) |
| Lunch | 665.88 (60.19) | 651.25 (47.03) | 712.17 (51.41) | 672.85 (28.72) | 583.45 (92.72) | 581.58 (68.57) | 526.42 (71.04) | 568.2 (72.63) |
| Afternoon | 641.85 (60.62) | 633.23 (65.83) | 734.84 (51.68) | 694.13 (38.3) | 621.02 (103.38) | 579.92 (79.68) | 560.72 (66.13) | 598.5 (83.1) |
| Dinner | 818.4 (78.73) | 802.12 (72.25) | 932.12 (59.79) | 869.74 (43.77) | 664.62 (140.54) | 655.12 (104.72) | 632.43 (88.23) | 693.39 (92.48) |
| Evening | 862.61 (108.42) | 848.6 (75.75) | 893.13 (63) | 840.66 (40.06) | 675.91 (116.7) | 659.24 (102.61) | 603.25 (71.52) | 652.44 (82.63) |
|  |  |  |  |  |  |  |  |  |
|  |  |  |  |  |  |  |  |  |
| ***Seattle, WA (n=4)*** |  |  |  |  |  |  |  |  |
| Total | 869.67 (32.89) | 880.97 (33.8) | 787.61 (32.61) | 797.55 (37.87) | 442.76 (77.92) | 479.02 (60.77) | 524.33 (77.77) | 543.59 (59.55) |
| *Time of day* |  |  |  |  |  |  |  |  |
| Late night | 1004.83 (62.45) | 1006.06 (58.22) | 957.39 (51.5) | 956.42 (63.98) | 435.86 (101.49) | 493.35 (88.64) | 544.92 (103.8) | 558.89 (84.59) |
| Breakfast | 620.54 (40.03) | 652.44 (65.82) | 554.38 (37.95) | 567.89 (43.86) | 245.87 (40.55) | 264.5 (33.29) | 286.68 (47.42) | 308.17 (43.63) |
| Lunch | 764.92 (41.07) | 775.27 (35.55) | 689.68 (28.81) | 697.43 (31.64) | 451.96 (77.78) | 462.08 (51.22) | 526.69 (79.13) | 526.19 (60.67) |
| Afternoon | 778.82 (46.33) | 784.92 (37.54) | 726.68 (40.24) | 732.12 (39.43) | 440.71 (77.89) | 476.8 (64.58) | 525.05 (81.01) | 526.28 (65.6) |
| Dinner | 970.34 (49) | 979.07 (41.13) | 929.84 (46.82) | 926.68 (47.01) | 485.15 (91.22) | 532.44 (74.03) | 613.43 (103.35) | 620.32 (82.36) |
| Evening | 980.22 (33.05) | 983.41 (43.23) | 897.74 (40.88) | 908.15 (46.71) | 466.27 (95.94) | 506.16 (75.96) | 554.99 (85.47) | 568.99 (71.69) |
|  |  |  |  |  |  |  |  |  |
| ***Cook County, IL (n=47)*** |  |  |  |  |  |  |  |  |
| Total | 742.12 (110.31) | 706.61 (110.24) | 739.45 (85.13) | 711.87 (85.52) | 488.74 (114.42) | 560.5 (142.5) | 496.13 (83.46) | 578.62 (111.31) |
| *Time of day* |  |  |  |  |  |  |  |  |
| Late night | 874 (151.18) | 830.36 (161.34) | 873.35 (103.7) | 831.95 (105.21) | 513.21 (128.57) | 588.48 (155.87) | 517.45 (89.42) | 613.55 (125.2) |
| Breakfast | 595.14 (78.1) | 550.79 (79.8) | 551.14 (55.38) | 506.74 (51.42) | 286.04 (94.83) | 330.84 (119.41) | 258.21 (35.46) | 296.67 (48.38) |
| Lunch | 648.71 (91.11) | 611.87 (88.52) | 654.38 (70.72) | 621.55 (64.89) | 485.02 (108.66) | 556.78 (138.05) | 491.95 (76.52) | 571.3 (104.29) |
| Afternoon | 653.52 (97.52) | 626.71 (95.92) | 663.53 (76.4) | 646 (76.25) | 492.57 (118.82) | 553.73 (148.1) | 492.39 (81.42) | 559.23 (109.02) |
| Dinner | 834.09 (130.29) | 801.49 (132.91) | 856.57 (103.28) | 826.9 (102.86) | 525.21 (134.14) | 609.2 (163.43) | 555.89 (103.15) | 652.88 (133.83) |
| Evening | 837.46 (121.7) | 796.73 (127.57) | 841.33 (92.71) | 803.11 (89.97) | 521.44 (128.49) | 594.5 (153.02) | 537.62 (90.77) | 626.25 (122.28) |
|  |  |  |  |  |  |  |  |  |
|  |  |  |  |  |  |  |  |  |
| ***CONDITIONAL***^b^ |  |  |  |  |  |  |  |  |
| ***Philadelphia, PA (n=4)*** | 956.11 (115.34) | 875.43 (99.52) | 999.11 (151.6) | 904.76 (101.37) | 1240.12 (168.19) | 1195.9 (190.96) | 1238.06 (194.55) | 1210.17 (186.98) |
| Total |  |  |  |  |  |  |  |  |
| *Time of day* |  |  |  |  |  |  |  |  |
| Late night | 1140.08 (103.47) | 1053.05 (130.65) | 1127.32 (88.39) | 1034.6 (142.56) | 1439 (129.52) | 1312.87 (199.21) | 1375.6 (110.86) | 1304.09 (195.31) |
| Breakfast | 698.01 (118.42) | 563.24 (103.77) | 792.93 (104.21) | 687.16 (87.25) | 1092.8 (147.23) | 1003.68 (128.69) | 1127.97 (99.28) | 1079.33 (104.54) |
| Lunch | 862.72 (99.76) | 790.68 (90.51) | 931.85 (116.95) | 822.58 (78.1) | 1260.52 (148.68) | 1166.09 (220.03) | 1249.56 (174.3) | 1188.72 (178.98) |
| Afternoon | 864.42 (116.07) | 804.14 (110.67) | 927.45 (146.92) | 843.01 (95.37) | 949.54 (172.9) | 1008.97 (188.82) | 947.54 (168.49) | 989.38 (180.37) |
| Dinner | 1035.08 (139.13) | 956.7 (112.68) | 1128.73 (186.93) | 1025.08 (130.03) | 1480.44 (190.48) | 1346.96 (228.07) | 1466.91 (236.12) | 1391.5 (228.46) |
| Evening | 1067.36 (110.01) | 979.84 (85.74) | 1092.11 (114.6) | 1001.39 (88.05) | 1438.7 (167.56) | 1330.24 (189.8) | 1412.64 (114) | 1343.63 (156.41) |
|  |  |  |  |  |  |  |  |  |
| ***Albany, CA (n=1)*** |  |  |  |  |  |  |  |  |
| Total | 983.32 (27.19) | 966.5 (24.62) | 959.97 (40.42) | 899.59 (24.42) | 1081.5 (86.06) | 1075.11 (113.43) | 1102.53 (95.4) | 1060.75 (119.29) |
| *Time of day* |  |  |  |  |  |  |  |  |
| Late night | 1138.6 (61.22) | 1113.63 (188.73) | 1058.6 (39.94) | 990.07 (51.19) | 1287.51 (167.64) | 1248.36 (224.25) | 1249.38 (94.66) | 1163.81 (148.37) |
| Breakfast | 634.24 (48.56) | 616.61 (37.34) | 680.4 (54.9) | 625.55 (20.06) | 1009.34 (88.45) | 936.97 (78.88) | 1053.75 (70.77) | 985.38 (55.6) |
| Lunch | 919.42 (24.99) | 905.25 (30.47) | 897.84 (47.5) | 839.57 (28.34) | 1212.37 (83.45) | 1151.16 (117.79) | 1178.11 (78.73) | 1107.47 (117.72) |
| Afternoon | 934.85 (42.6) | 921.47 (43.91) | 914.75 (37.67) | 861.33 (28.67) | 746.3 (116.84) | 810.41 (106.53) | 789.89 (113.78) | 804.14 (107.72) |
| Dinner | 1133.31 (36.75) | 1106.8 (41.63) | 1122.77 (40.73) | 1059.91 (25.91) | 1316.83 (82.47) | 1247.92 (149.76) | 1347.64 (84.2) | 1255.42 (145.26) |
| Evening | 1143.51 (28.9) | 1105.66 (77.66) | 1055.74 (49.58) | 999.36 (33.39) | 1323.53 (79.04) | 1251.26 (162.62) | 1286.04 (82.38) | 1190.26 (143.7) |
|  |  |  |  |  |  |  |  |  |
|  |  |  |  |  |  |  |  |  |
| ***Oakland, CA (n=4)*** |  |  |  |  |  |  |  |  |
| Total | 930.99 (70) | 902.58 (67.19) | 968.19 (55.8) | 921.52 (32.11) | 1261.86 (130.46) | 1170.75 (125.35) | 1269.11 (123.02) | 1201.14 (137.03) |
| *Time of day* |  |  |  |  |  |  |  |  |
| Late night | 1061.18 (158.52) | 1018.65 (122.41) | 1044.65 (59.71) | 1005.67 (43.73) | 1380.58 (132.83) | 1300.55 (144.47) | 1375.07 (132.26) | 1283.23 (158.03) |
| Breakfast | 645.58 (44.89) | 636.17 (39.36) | 675.77 (39.1) | 653.32 (32.4) | 1139.56 (91.52) | 1042.85 (94.91) | 1173.12 (97.07) | 1089.77 (92.03) |
| Lunch | 857.42 (57.78) | 831.38 (72.69) | 892.76 (49.71) | 857.58 (30.18) | 1328.62 (124.85) | 1228.3 (119.25) | 1287.93 (110.8) | 1199.37 (138.95) |
| Afternoon | 853.24 (55.71) | 833.08 (69.96) | 915.12 (54.09) | 881.98 (38.89) | 935.27 (135.87) | 864.32 (131.53) | 944.97 (108.29) | 944.17 (126.38) |
| Dinner | 998.71 (70.78) | 972.84 (80.01) | 1094.4 (56.73) | 1036.62 (38.95) | 1450.9 (161.2) | 1351.86 (162.92) | 1496.93 (133.91) | 1387.14 (167.1) |
| Evening | 1045.83 (98.41) | 1020.44 (74.87) | 1055.76 (60.99) | 1007.01 (37.92) | 1456.68 (140.24) | 1354.34 (147.92) | 1423.9 (125.87) | 1325.56 (158.68) |
|  |  |  |  |  |  |  |  |  |
|  |  |  |  |  |  |  |  |  |
| ***Seattle, WA (n=4)*** |  |  |  |  |  |  |  |  |
| Total | 975.85 (58.83) | 994.96 (56.53) | 903.9 (40.5) | 911.71 (53.26) | 1173.33 (136.81) | 1123.93 (105.97) | 1206.61 (128.25) | 1148.02 (97.11) |
| *Time of day* |  |  |  |  |  |  |  |  |
| Late night | 1111.39 (62.81) | 1117.34 (50.51) | 1074.77 (55.39) | 1064.74 (70.57) | 1280.26 (166.51) | 1223.32 (141.33) | 1346.6 (161.11) | 1249.14 (125.35) |
| Breakfast | 686.88 (51.47) | 728.75 (83.79) | 626.12 (47) | 637.81 (52.89) | 1057.64 (100.25) | 1013.17 (94.05) | 1104.33 (81.85) | 1048.34 (61.68) |
| Lunch | 879.3 (83.26) | 902.63 (81.52) | 818.1 (44.33) | 814.59 (55.11) | 1223.74 (136.79) | 1153.42 (105.08) | 1238.6 (124.82) | 1157.61 (96.48) |
| Afternoon | 893.85 (69.63) | 912.43 (66.06) | 850.25 (49.09) | 851 (52.87) | 894.32 (134.05) | 879.08 (97.11) | 936.21 (132.36) | 896.38 (90.03) |
| Dinner | 1075.12 (67.9) | 1087.68 (61.19) | 1041.15 (55.09) | 1034.23 (60.68) | 1317.69 (158.35) | 1256.8 (136.68) | 1426.3 (154.76) | 1321.24 (128.8) |
| Evening | 1086.31 (49.16) | 1089.99 (58) | 1012.56 (43.09) | 1017.52 (55.79) | 1296.22 (151.73) | 1225.12 (134.52) | 1339.36 (137.18) | 1251.22 (117.58) |
|  |  |  |  |  |  |  |  |  |
| ***Cook County, IL (n=47)*** |  |  |  |  |  |  |  |  |
| Total | 904.23 (103.96) | 878.52 (113.81) | 895.77 (76.41) | 879.04 (79.39) | 1163.75 (145.21) | 1208.97 (173.24) | 1166.56 (134.12) | 1240.98 (170.23) |
| *Time of day* |  |  |  |  |  |  |  |  |
| Late night | 1032.35 (143.04) | 997.28 (170.04) | 1029.18 (88.63) | 1004.3 (94.76) | 1303.63 (178.41) | 1333.01 (207) | 1305.56 (140.98) | 1367.81 (173.76) |
| Breakfast | 696.36 (88.18) | 660.3 (94.66) | 636.33 (57.68) | 599.94 (54.38) | 1110.78 (137.74) | 1094.53 (151.78) | 1089.44 (90.71) | 1064.99 (92.65) |
| Lunch | 824.86 (95.52) | 797.98 (106.57) | 828.36 (65.07) | 800.91 (61.1) | 1173.98 (146.73) | 1208.13 (169.79) | 1188.58 (130.34) | 1239.56 (156.61) |
| Afternoon | 825.87 (93.23) | 809.19 (100.45) | 829.83 (67.51) | 820.49 (69.37) | 907.2 (142.07) | 971.41 (183.84) | 872.66 (117.76) | 962.02 (165.8) |
| Dinner | 991.65 (119.6) | 970.64 (130.76) | 1011.12 (88.66) | 994.22 (87.55) | 1334.58 (169.42) | 1376.91 (188.87) | 1367.23 (161.7) | 1434.74 (188.13) |
| Evening | 997.2 (108.82) | 964.22 (129.53) | 995.77 (77.96) | 972.29 (73.79) | 1315.12 (155.23) | 1342.33 (176.94) | 1327.38 (139.97) | 1383.71 (171.63) |

SD=standard deviation

^a^Unconditional on a transaction including a beverage item.

^b^Conditional on a transaction including a beverage item.

**Table C.3.** Descriptive statistics of secondary outcomes, beverage sugar (g), overall and by location and time of day

|  | *Beverage sugar (g),  single items* | | | | *Beverage sugar (g),  combo meals* | | | |
| --- | --- | --- | --- | --- | --- | --- | --- | --- |
|  | Tax group | | Comparison group | | Tax group | | Comparison group | |
|  | Baseline | Follow-up | Baseline | Follow-up | Baseline | Follow-up | Baseline | Follow-up |
|  | Mean (SD) | | Mean (SD) | | Mean (SD) | | Mean (SD) | |
| ***UNCONDITIONAL***^a^ |  |  |  |  |  |  |  |  |
| ***Philadelphia, PA (n=4)*** |  |  |  |  |  |  |  |  |
| Total | 11.8 (2.3) | 10.0 (2.0) | 14.8 (3.1) | 13.1 (2.8) | 32.7 (6.7) | 37.4 (7.3) | 32.0 (5.6) | 36.3 (4.9) |
| *Time of day* |  |  |  |  |  |  |  |  |
| Late night | 14.6 (4.4) | 11.9 (3.2) | 15.7 (3.1) | 14.1 (3.7) | 28.8 (8.9) | 35.3 (10.3) | 27.6 (5.1) | 34.0 (9.9) |
| Breakfast | 9.8 (1.9) | 8.2 (1.5) | 14.7 (2.4) | 12.8 (2.5) | 14.1 (3.6) | 16.4 (4.8) | 18.1 (7.2) | 21.6 (8.2) |
| Lunch | 13.7 (2.0) | 11.4 (2.2) | 17.5 (3.7) | 15.0 (3.3) | 32.5 (5.9) | 36.6 (8.1) | 31.1 (5.8) | 36.0 (5.1) |
| Afternoon | 4.1 (1.5) | 4.2 (1.8) | 8.4 (3.0) | 8.1 (2.8) | 44.0 (8.3) | 44.6 (7.0) | 40.2 (5.3) | 41.5 (4.7) |
| Dinner | 13.9 (2.6) | 11.6 (2.6) | 16.4 (3.1) | 14.0 (2.7) | 30.3 (7.7) | 36.5 (8.8) | 30.8 (4.7) | 37.1 (4.7) |
| Evening | 15.1 (3.6) | 12.7 (2.6) | 16.8 (2.9) | 14.7 (2.8) | 30.2 (9.1) | 37.2 (9.8) | 31.7 (6.0) | 37.2 (5.4) |
|  |  |  |  |  |  |  |  |  |
| ***Albany, CA (n=1)*** |  |  |  |  |  |  |  |  |
| Total | 10.0 (0.7) | 9.4 (0.6) | 14.4 (1.3) | 13.0 (0.6) | 22.0 (1.7) | 24.2 (1.1) | 23.3 (2.1) | 25.7 (1.0) |
| *Time of day* |  |  |  |  |  |  |  |  |
| Late night | 9.9 (2.6) | 8.9 (4.4) | 15.9 (1.6) | 14.6 (1.3) | 21.1 (5.2) | 23.1 (5.2) | 24.2 (3.0) | 27.3 (1.8) |
| Breakfast | 7.2 (1.7) | 7.6 (1.1) | 12.5 (1.1) | 10.9 (0.7) | 10.1 (1.3) | 12.8 (1.5) | 11.2 (1.1) | 13.4 (1.1) |
| Lunch | 13.9 (1.8) | 12.0 (0.9) | 17.1 (1.6) | 15.6 (0.7) | 21.3 (2.6) | 24.4 (0.9) | 22.8 (2.5) | 25.6 (1.4) |
| Afternoon | 5.0 (0.5) | 5.4 (0.8) | 7.9 (1.0) | 7.7 (0.5) | 32.6 (2.5) | 30.9 (2.3) | 34.3 (1.6) | 33.6 (1.2) |
| Dinner | 11.6 (0.9) | 10.6 (1.2) | 16.1 (1.6) | 14.5 (0.8) | 20.6 (2.1) | 24.1 (1.6) | 22.2 (3.1) | 25.8 (1.6) |
| Evening | 10.7 (1.2) | 10.3 (1.5) | 16.8 (1.4) | 15.4 (1.1) | 21.0 (2.7) | 24.8 (1.6) | 23.3 (2.4) | 26.7 (1.6) |
|  |  |  |  |  |  |  |  |  |
|  |  |  |  |  |  |  |  |  |
| ***Oakland, CA (n=4)*** |  |  |  |  |  |  |  |  |
| Total | 11.4 (1.4) | 9.9 (1.1) | 13.6 (1.4) | 11.9 (0.7) | 34.7 (4.9) | 32.7 (3.3) | 31.5 (2.2) | 33.6 (1.9) |
| *Time of day* |  |  |  |  |  |  |  |  |
| Late night | 13.5 (2.0) | 11.7 (1.9) | 15.4 (1.5) | 13.9 (1.5) | 34.3 (5.6) | 33.4 (5.5) | 32.0 (3.4) | 34.5 (2.7) |
| Breakfast | 9.4 (1.2) | 8.5 (1.3) | 12.4 (1.2) | 11.6 (1.4) | 15.3 (3.1) | 17.3 (2.9) | 14.2 (1.7) | 16.6 (1.4) |
| Lunch | 14.7 (2.6) | 12.2 (1.3) | 15.7 (1.7) | 13.6 (0.9) | 31.9 (3.9) | 31.7 (2.5) | 28.8 (2.6) | 31.2 (2.0) |
| Afternoon | 4.1 (0.9) | 4.5 (1.2) | 6.9 (0.7) | 6.4 (0.6) | 48.1 (7.0) | 39.0 (3.4) | 42.1 (2.2) | 39.8 (2.1) |
| Dinner | 13.1 (1.9) | 10.9 (1.1) | 15.2 (1.5) | 13.0 (1.0) | 33.7 (6.1) | 32.7 (3.9) | 31.2 (3.1) | 35.2 (2.4) |
| Evening | 13.4 (1.7) | 11.6 (1.5) | 15.5 (1.5) | 13.7 (1.1) | 35.5 (5.9) | 34.6 (5.0) | 32.8 (2.6) | 35.9 (2.4) |
|  |  |  |  |  |  |  |  |  |
|  |  |  |  |  |  |  |  |  |
| ***Seattle, WA (n=4)*** |  |  |  |  |  |  |  |  |
| Total | 11.0 (1.1) | 11.3 (1.1) | 12.6 (0.9) | 12.8 (0.8) | 24.4 (1.8) | 26.1 (1.8) | 29.7 (1.9) | 30.7 (1.6) |
| *Time of day* |  |  |  |  |  |  |  |  |
| Late night | 11.6 (2.2) | 11.7 (1.9) | 14.7 (2.3) | 14.8 (2.3) | 23.2 (3.1) | 25.9 (3.6) | 29.7 (3.2) | 31.2 (3.9) |
| Breakfast | 8.5 (1.5) | 9.0 (1.4) | 11.1 (1.0) | 11.5 (1.3) | 11.5 (1.5) | 12.7 (1.7) | 14.7 (1.9) | 16.2 (2.4) |
| Lunch | 12.6 (1.2) | 12.6 (1.4) | 14.6 (0.9) | 14.8 (1.1) | 24.7 (2.0) | 25.1 (1.5) | 29.3 (2.2) | 29.9 (2.2) |
| Afternoon | 6.0 (0.8) | 6.5 (1.1) | 6.9 (0.8) | 7.4 (1.1) | 30.8 (2.7) | 31.9 (2.9) | 36.8 (2.1) | 36.0 (1.9) |
| Dinner | 12.6 (1.7) | 13.0 (1.7) | 13.8 (1.3) | 13.9 (1.5) | 24.5 (2.1) | 26.8 (2.0) | 30.8 (2.8) | 32.0 (2.6) |
| Evening | 12.6 (2.0) | 13.1 (2.0) | 14.8 (1.7) | 15.0 (1.9) | 24.4 (2.8) | 26.5 (2.8) | 30.3 (2.5) | 31.4 (2.4) |
|  |  |  |  |  |  |  |  |  |
| ***Cook County, IL (n=47)*** |  |  |  |  |  |  |  |  |
| Total | 10.5 (1.8) | 8.7 (1.6) | 12.6 (2.0) | 11.1 (1.8) | 28.2 (5.4) | 29.2 (5.0) | 29.5 (3.8) | 30.7 (3.3) |
| *Time of day* |  |  |  |  |  |  |  |  |
| Late night | 11.9 (3.2) | 10.2 (3.5) | 14.0 (3.1) | 12.2 (2.8) | 28.4 (6.5) | 30.6 (6.6) | 29.2 (4.4) | 32.1 (4.2) |
| Breakfast | 10.0 (2.6) | 8.4 (2.2) | 12.0 (1.7) | 10.4 (1.6) | 15.3 (5.8) | 16.4 (5.9) | 13.9 (2.1) | 15.3 (2.1) |
| Lunch | 11.9 (2.1) | 9.9 (1.8) | 14.6 (2.2) | 12.7 (1.8) | 28.2 (5.3) | 29.7 (5.2) | 29.1 (3.7) | 31.1 (3.4) |
| Afternoon | 5.7 (1.9) | 4.7 (1.6) | 6.9 (1.8) | 6.5 (1.6) | 35.6 (6.8) | 33.9 (5.1) | 38.1 (4.4) | 35.9 (3.1) |
| Dinner | 11.5 (1.9) | 9.5 (1.7) | 13.9 (2.3) | 11.9 (1.9) | 26.7 (5.9) | 28.6 (5.7) | 29.1 (4.2) | 31.5 (4.0) |
| Evening | 12.4 (2.3) | 10.4 (2.2) | 14.2 (2.5) | 12.3 (2.1) | 28.4 (5.9) | 30.0 (5.7) | 30.0 (4.2) | 32.2 (3.9) |
|  |  |  |  |  |  |  |  |  |
|  |  |  |  |  |  |  |  |  |
| ***CONDITIONAL***^b^ |  |  |  |  |  |  |  |  |
| ***Philadelphia, PA (n=4)*** |  |  |  |  |  |  |  |  |
| Total | 63.9 (5.2) | 63.6 (6.1) | 67.3 (7.5) | 66.0 (6.8) | 91.4 (4.4) | 91.6 (7.7) | 89.6 (4.0) | 90.2 (4.1) |
| *Time of day* |  |  |  |  |  |  |  |  |
| Late night | 69.5 (8.6) | 68.7 (11.5) | 74.6 (7.4) | 72.7 (7.4) | 97.9 (7.0) | 95.1 (12.7) | 95.0 (4.2) | 96.4 (14.4) |
| Breakfast | 42.0 (6.8) | 39.2 (6.7) | 58.6 (6.1) | 55.9 (6.4) | 63.9 (13.5) | 66.0 (8.6) | 72.7 (7.8) | 73.0 (7.7) |
| Lunch | 60.9 (3.5) | 59.9 (7.6) | 64.4 (7.1) | 62.5 (6.9) | 90.4 (4.8) | 87.7 (12.3) | 87.7 (3.8) | 86.9 (3.8) |
| Afternoon | 58.4 (12.3) | 58.5 (13.3) | 65.1 (9.8) | 62.5 (9.7) | 86.2 (4.1) | 88.1 (7.2) | 85.8 (4.1) | 86.7 (4.5) |
| Dinner | 67.8 (5.8) | 67.5 (9.7) | 72.1 (9.4) | 71.2 (7.4) | 97.6 (5.2) | 95.4 (12.5) | 96.5 (6.0) | 97.1 (5.0) |
| Evening | 71.1 (7.9) | 70.8 (8.0) | 75.6 (5.9) | 74.2 (6.2) | 98.1 (6.5) | 97.9 (8.6) | 97.2 (4.0) | 98.0 (4.2) |
|  |  |  |  |  |  |  |  |  |
| ***Albany, CA (n=1)*** |  |  |  |  |  |  |  |  |
| Total | 54.0 (2.1) | 52.1 (2.4) | 58.8 (1.3) | 58.1 (0.8) | 78.6 (1.6) | 78.0 (1.8) | 79.2 (1.4) | 78.9 (1.4) |
| *Time of day* |  |  |  |  |  |  |  |  |
| Late night | 70.0 (13.8) | 49.6 (20.6) | 67.8 (3.7) | 69.1 (2.4) | 84.8 (12.5) | 88.2 (12.2) | 88.0 (3.9) | 86.4 (2.4) |
| Breakfast | 31.3 (3.4) | 36.3 (3.5) | 43.5 (2.7) | 42.6 (2.1) | 56.0 (3.6) | 56.6 (3.9) | 59.9 (2.2) | 60.2 (2.6) |
| Lunch | 51.7 (2.6) | 50.0 (2.3) | 55.8 (1.2) | 56.2 (1.0) | 80.2 (3.0) | 76.9 (2.7) | 77.0 (2.5) | 77.0 (1.8) |
| Afternoon | 51.9 (5.4) | 50.4 (5.6) | 56.3 (2.7) | 55.3 (1.7) | 74.9 (2.9) | 76.0 (2.6) | 78.8 (1.6) | 78.2 (2.0) |
| Dinner | 63.0 (4.1) | 59.3 (3.5) | 66.3 (1.4) | 65.9 (1.6) | 85.3 (3.1) | 83.0 (3.0) | 85.5 (2.4) | 85.9 (1.8) |
| Evening | 69.3 (4.0) | 59.6 (6.7) | 69.9 (1.8) | 69.2 (1.4) | 86.8 (3.5) | 87.1 (3.6) | 87.6 (3.0) | 86.5 (2.4) |
|  |  |  |  |  |  |  |  |  |
|  |  |  |  |  |  |  |  |  |
| ***Oakland, CA (n=4)*** |  |  |  |  |  |  |  |  |
| Total | 60.8 (5.4) | 57.3 (4.1) | 64.2 (2.5) | 62.8 (1.9) | 91.0 (6.3) | 90.9 (4.8) | 88.6 (1.4) | 89.0 (2.7) |
| *Time of day* |  |  |  |  |  |  |  |  |
| Late night | 67.5 (6.8) | 63.1 (8.5) | 72.9 (3.9) | 71.7 (3.5) | 94.0 (6.4) | 96.4 (6.5) | 95.4 (2.9) | 94.8 (3.7) |
| Breakfast | 39.2 (4.8) | 38.8 (5.8) | 47.0 (2.7) | 47.9 (4.2) | 64.6 (6.0) | 65.5 (7.0) | 69.6 (3.7) | 70.6 (3.1) |
| Lunch | 60.5 (5.8) | 57.4 (3.8) | 58.9 (2.1) | 58.0 (2.2) | 88.5 (5.8) | 89.3 (4.5) | 83.2 (1.9) | 83.5 (2.8) |
| Afternoon | 52.7 (6.5) | 51.5 (5.9) | 59.4 (4.1) | 59.2 (3.5) | 89.6 (6.7) | 87.7 (5.0) | 86.4 (1.9) | 86.0 (2.8) |
| Dinner | 65.0 (6.7) | 61.3 (4.6) | 70.0 (2.3) | 68.3 (1.8) | 95.3 (9.2) | 95.7 (6.0) | 94.0 (2.4) | 94.9 (2.7) |
| Evening | 68.5 (6.0) | 64.1 (6.5) | 74.0 (2.6) | 72.1 (2.3) | 97.0 (7.5) | 97.6 (6.3) | 95.4 (1.8) | 96.0 (2.7) |
|  |  |  |  |  |  |  |  |  |
|  |  |  |  |  |  |  |  |  |
| ***Seattle, WA (n=4)*** |  |  |  |  |  |  |  |  |
| Total | 54.9 (6.2) | 57.2 (6.0) | 59.0 (1.6) | 60.6 (1.8) | 80.0 (4.6) | 82.3 (5.1) | 84.1 (2.5) | 85.3 (2.8) |
| *Time of day* |  |  |  |  |  |  |  |  |
| Late night | 59.1 (9.1) | 59.9 (6.4) | 67.8 (3.2) | 68.9 (6.2) | 84.2 (6.7) | 85.3 (5.6) | 90.8 (3.9) | 91.2 (4.4) |
| Breakfast | 37.4 (5.7) | 42.1 (6.7) | 44.4 (3.0) | 47.6 (2.4) | 56.8 (7.5) | 59.8 (5.8) | 65.1 (3.5) | 67.0 (3.9) |
| Lunch | 53.0 (6.9) | 54.7 (7.5) | 55.6 (1.9) | 57.4 (2.9) | 79.9 (4.1) | 81.0 (5.4) | 81.3 (2.4) | 82.3 (2.7) |
| Afternoon | 49.5 (10.7) | 52.8 (8.9) | 54.4 (3.8) | 57.2 (4.2) | 76.9 (4.5) | 79.1 (6.0) | 81.5 (2.7) | 81.6 (2.7) |
| Dinner | 59.2 (5.7) | 60.3 (6.0) | 65.5 (2.8) | 66.4 (3.0) | 83.9 (5.6) | 86.7 (5.8) | 90.6 (3.5) | 91.6 (3.3) |
| Evening | 62.0 (6.7) | 63.8 (5.7) | 68.9 (2.3) | 70.2 (2.9) | 84.6 (5.9) | 86.7 (5.7) | 90.6 (2.8) | 91.1 (3.1) |
|  |  |  |  |  |  |  |  |  |
| ***Cook County, IL (n=47)*** |  |  |  |  |  |  |  |  |
| Total | 61.5 (6.4) | 58.8 (6.0) | 62.6 (5.1) | 60.8 (4.9) | 83.7 (5.8) | 83.3 (5.6) | 85.4 (4.0) | 85.3 (4.1) |
| *Time of day* |  |  |  |  |  |  |  |  |
| Late night | 69.5 (10.2) | 67.2 (10.2) | 73.2 (9.5) | 71.6 (10.9) | 89.0 (8.6) | 89.1 (8.7) | 91.8 (4.8) | 91.8 (4.0) |
| Breakfast | 46.4 (9.8) | 44.2 (10.2) | 47.2 (5.3) | 45.2 (5.5) | 68.5 (11.2) | 65.8 (10.0) | 67.0 (5.1) | 64.7 (5.4) |
| Lunch | 56.7 (6.6) | 54.5 (6.0) | 58.6 (5.1) | 57.1 (4.9) | 80.8 (5.8) | 80.4 (5.8) | 82.3 (3.5) | 82.4 (3.6) |
| Afternoon | 59.3 (9.9) | 54.3 (9.5) | 59.4 (7.2) | 57.7 (6.8) | 80.5 (6.5) | 80.1 (6.2) | 82.8 (4.4) | 82.6 (4.2) |
| Dinner | 65.5 (6.3) | 63.1 (6.5) | 67.7 (4.8) | 66.3 (4.8) | 87.7 (6.1) | 87.8 (6.0) | 91.2 (4.2) | 91.6 (4.6) |
| Evening | 70.4 (6.5) | 67.9 (6.0) | 71.7 (4.3) | 70.0 (4.2) | 89.6 (5.5) | 89.0 (5.4) | 92.3 (3.7) | 92.1 (3.8) |

SD=standard deviation

^a^Unconditional on a transaction including a beverage item.

^b^Conditional on a transaction including a beverage item.

**Table C.4.** Descriptive statistics of secondary outcomes, beverage count, overall and by location and time of day

|  | *Beverage count,  single items* | | | | *Beverage count,  combo meals* | | | |
| --- | --- | --- | --- | --- | --- | --- | --- | --- |
|  | Tax group | | Comparison group | | Tax group | | Comparison group | |
|  | Baseline | Follow-up | Baseline | Follow-up | Baseline | Follow-up | Baseline | Follow-up |
|  | Mean (SD) | | Mean (SD) | | Mean (SD) | | Mean (SD) | |
| ***UNCONDITIONAL***^a^ |  |  |  |  |  |  |  |  |
| ***Philadelphia, PA (n=4)*** |  |  |  |  |  |  |  |  |
| Total | 0.21 (0.04) | 0.18 (0.03) | 0.26 (0.04) | 0.23 (0.04) | 0.45 (0.1) | 0.52 (0.09) | 0.45 (0.08) | 0.51 (0.06) |
| *Time of day* |  |  |  |  |  |  |  |  |
| Late night | 0.25 (0.06) | 0.2 (0.05) | 0.25 (0.04) | 0.23 (0.05) | 0.37 (0.12) | 0.47 (0.13) | 0.35 (0.07) | 0.46 (0.12) |
| Breakfast | 0.27 (0.06) | 0.23 (0.05) | 0.29 (0.04) | 0.26 (0.06) | 0.27 (0.07) | 0.3 (0.07) | 0.29 (0.08) | 0.35 (0.09) |
| Lunch | 0.26 (0.04) | 0.22 (0.09) | 0.31 (0.06) | 0.27 (0.05) | 0.43 (0.09) | 0.51 (0.11) | 0.42 (0.08) | 0.5 (0.06) |
| Afternoon | 0.07 (0.02) | 0.07 (0.03) | 0.14 (0.04) | 0.15 (0.04) | 0.64 (0.13) | 0.65 (0.09) | 0.6 (0.07) | 0.61 (0.06) |
| Dinner | 0.24 (0.04) | 0.2 (0.04) | 0.28 (0.04) | 0.24 (0.04) | 0.4 (0.11) | 0.5 (0.12) | 0.41 (0.07) | 0.51 (0.06) |
| Evening | 0.25 (0.05) | 0.21 (0.03) | 0.27 (0.04) | 0.24 (0.04) | 0.39 (0.13) | 0.49 (0.12) | 0.41 (0.09) | 0.5 (0.08) |
|  |  |  |  |  |  |  |  |  |
| ***Albany, CA (n=1)*** |  |  |  |  |  |  |  |  |
| Total | 0.21 (0.02) | 0.2 (0.01) | 0.29 (0.03) | 0.26 (0.01) | 0.34 (0.03) | 0.38 (0.02) | 0.35 (0.03) | 0.39 (0.01) |
| *Time of day* |  |  |  |  |  |  |  |  |
| Late night | 0.16 (0.04) | 0.22 (0.11) | 0.28 (0.03) | 0.25 (0.02) | 0.29 (0.07) | 0.33 (0.07) | 0.32 (0.05) | 0.38 (0.03) |
| Breakfast | 0.25 (0.04) | 0.23 (0.03) | 0.32 (0.01) | 0.29 (0.02) | 0.21 (0.02) | 0.27 (0.04) | 0.21 (0.02) | 0.26 (0.02) |
| Lunch | 0.3 (0.03) | 0.27 (0.02) | 0.36 (0.03) | 0.32 (0.02) | 0.31 (0.04) | 0.37 (0.01) | 0.34 (0.04) | 0.39 (0.02) |
| Afternoon | 0.11 (0.01) | 0.12 (0.01) | 0.16 (0.02) | 0.16 (0.01) | 0.54 (0.04) | 0.5 (0.04) | 0.55 (0.03) | 0.53 (0.03) |
| Dinner | 0.21 (0.02) | 0.21 (0.02) | 0.3 (0.03) | 0.26 (0.02) | 0.29 (0.03) | 0.35 (0.02) | 0.32 (0.05) | 0.38 (0.02) |
| Evening | 0.18 (0.01) | 0.2 (0.03) | 0.29 (0.03) | 0.27 (0.02) | 0.29 (0.04) | 0.35 (0.02) | 0.31 (0.04) | 0.37 (0.02) |
|  |  |  |  |  |  |  |  |  |
|  |  |  |  |  |  |  |  |  |
| ***Oakland, CA (n=4)*** |  |  |  |  |  |  |  |  |
| Total | 0.22 (0.02) | 0.2 (0.02) | 0.25 (0.03) | 0.22 (0.02) | 0.49 (0.05) | 0.46 (0.04) | 0.46 (0.03) | 0.49 (0.02) |
| *Time of day* |  |  |  |  |  |  |  |  |
| Late night | 0.24 (0.03) | 0.22 (0.03) | 0.26 (0.03) | 0.23 (0.03) | 0.46 (0.06) | 0.44 (0.06) | 0.42 (0.04) | 0.46 (0.03) |
| Breakfast | 0.27 (0.03) | 0.25 (0.03) | 0.3 (0.02) | 0.28 (0.02) | 0.28 (0.04) | 0.31 (0.04) | 0.25 (0.03) | 0.29 (0.02) |
| Lunch | 0.29 (0.06) | 0.24 (0.02) | 0.31 (0.03) | 0.27 (0.02) | 0.44 (0.04) | 0.43 (0.03) | 0.42 (0.03) | 0.46 (0.03) |
| Afternoon | 0.09 (0.02) | 0.1 (0.05) | 0.13 (0.02) | 0.12 (0.01) | 0.74 (0.11) | 0.58 (0.06) | 0.65 (0.04) | 0.61 (0.04) |
| Dinner | 0.24 (0.03) | 0.21 (0.02) | 0.27 (0.03) | 0.23 (0.02) | 0.46 (0.06) | 0.45 (0.04) | 0.44 (0.04) | 0.5 (0.03) |
| Evening | 0.24 (0.02) | 0.22 (0.02) | 0.25 (0.03) | 0.23 (0.02) | 0.47 (0.06) | 0.46 (0.05) | 0.44 (0.03) | 0.49 (0.03) |
|  |  |  |  |  |  |  |  |  |
|  |  |  |  |  |  |  |  |  |
| ***Seattle, WA (n=4)*** |  |  |  |  |  |  |  |  |
| Total | 0.23 (0.02) | 0.23 (0.01) | 0.25 (0.02) | 0.25 (0.01) | 0.37 (0.02) | 0.39 (0.03) | 0.44 (0.03) | 0.45 (0.03) |
| *Time of day* |  |  |  |  |  |  |  |  |
| Late night | 0.23 (0.03) | 0.23 (0.02) | 0.26 (0.04) | 0.26 (0.04) | 0.33 (0.04) | 0.37 (0.05) | 0.41 (0.05) | 0.43 (0.06) |
| Breakfast | 0.25 (0.03) | 0.24 (0.03) | 0.28 (0.02) | 0.27 (0.02) | 0.24 (0.03) | 0.25 (0.03) | 0.27 (0.03) | 0.29 (0.04) |
| Lunch | 0.27 (0.03) | 0.26 (0.03) | 0.3 (0.02) | 0.29 (0.01) | 0.36 (0.03) | 0.36 (0.03) | 0.43 (0.03) | 0.43 (0.04) |
| Afternoon | 0.14 (0.03) | 0.14 (0.03) | 0.15 (0.01) | 0.15 (0.02) | 0.48 (0.04) | 0.49 (0.05) | 0.57 (0.03) | 0.55 (0.04) |
| Dinner | 0.25 (0.02) | 0.25 (0.02) | 0.25 (0.02) | 0.25 (0.02) | 0.35 (0.03) | 0.38 (0.03) | 0.44 (0.04) | 0.45 (0.04) |
| Evening | 0.24 (0.06) | 0.24 (0.03) | 0.26 (0.03) | 0.26 (0.03) | 0.35 (0.03) | 0.38 (0.04) | 0.42 (0.04) | 0.43 (0.04) |
|  |  |  |  |  |  |  |  |  |
| ***Cook County, IL (n=47)*** |  |  |  |  |  |  |  |  |
| Total | 0.2 (0.04) | 0.17 (0.04) | 0.24 (0.04) | 0.21 (0.04) | 0.41 (0.07) | 0.43 (0.07) | 0.43 (0.05) | 0.45 (0.05) |
| *Time of day* |  |  |  |  |  |  |  |  |
| Late night | 0.2 (0.06) | 0.18 (0.09) | 0.24 (0.08) | 0.2 (0.04) | 0.39 (0.09) | 0.42 (0.09) | 0.39 (0.06) | 0.44 (0.06) |
| Breakfast | 0.24 (0.05) | 0.21 (0.04) | 0.29 (0.04) | 0.27 (0.06) | 0.25 (0.07) | 0.29 (0.07) | 0.24 (0.03) | 0.28 (0.03) |
| Lunch | 0.23 (0.04) | 0.2 (0.04) | 0.28 (0.04) | 0.26 (0.05) | 0.41 (0.07) | 0.43 (0.07) | 0.42 (0.05) | 0.45 (0.04) |
| Afternoon | 0.11 (0.04) | 0.1 (0.03) | 0.13 (0.03) | 0.13 (0.03) | 0.54 (0.1) | 0.51 (0.07) | 0.58 (0.06) | 0.54 (0.04) |
| Dinner | 0.2 (0.04) | 0.17 (0.04) | 0.25 (0.04) | 0.21 (0.04) | 0.38 (0.08) | 0.41 (0.08) | 0.41 (0.06) | 0.45 (0.05) |
| Evening | 0.21 (0.04) | 0.18 (0.04) | 0.24 (0.04) | 0.21 (0.04) | 0.39 (0.08) | 0.42 (0.08) | 0.41 (0.06) | 0.44 (0.05) |
|  |  |  |  |  |  |  |  |  |
|  |  |  |  |  |  |  |  |  |
| ***CONDITIONAL***^b^ |  |  |  |  |  |  |  |  |
| ***Philadelphia, PA (n=4)*** |  |  |  |  |  |  |  |  |
| Total | 1.16 (0.05) | 1.14 (0.04) | 1.18 (0.06) | 1.17 (0.06) | 1.24 (0.08) | 1.28 (0.08) | 1.25 (0.06) | 1.28 (0.06) |
| *Time of day* |  |  |  |  |  |  |  |  |
| Late night | 1.19 (0.07) | 1.15 (0.14) | 1.18 (0.05) | 1.18 (0.07) | 1.25 (0.08) | 1.28 (0.16) | 1.21 (0.05) | 1.29 (0.18) |
| Breakfast | 1.14 (0.07) | 1.1 (0.07) | 1.16 (0.04) | 1.14 (0.07) | 1.19 (0.1) | 1.21 (0.07) | 1.19 (0.06) | 1.2 (0.07) |
| Lunch | 1.13 (0.04) | 1.11 (0.04) | 1.16 (0.05) | 1.15 (0.05) | 1.19 (0.07) | 1.22 (0.15) | 1.19 (0.04) | 1.22 (0.05) |
| Afternoon | 1.09 (0.05) | 1.1 (0.05) | 1.14 (0.04) | 1.14 (0.07) | 1.26 (0.07) | 1.28 (0.07) | 1.27 (0.06) | 1.28 (0.06) |
| Dinner | 1.18 (0.05) | 1.15 (0.13) | 1.22 (0.05) | 1.2 (0.05) | 1.27 (0.09) | 1.3 (0.16) | 1.29 (0.05) | 1.34 (0.06) |
| Evening | 1.18 (0.07) | 1.16 (0.05) | 1.22 (0.05) | 1.19 (0.05) | 1.25 (0.09) | 1.31 (0.09) | 1.26 (0.05) | 1.31 (0.07) |
|  |  |  |  |  |  |  |  |  |
| ***Albany, CA (n=1)*** |  |  |  |  |  |  |  |  |
| Total | 1.14 (0.02) | 1.13 (0.02) | 1.18 (0.02) | 1.16 (0.01) | 1.21 (0.01) | 1.21 (0.02) | 1.19 (0.01) | 1.21 (0.01) |
| *Time of day* |  |  |  |  |  |  |  |  |
| Late night | 1.14 (0.11) | 1.19 (0.22) | 1.21 (0.02) | 1.19 (0.04) | 1.18 (0.12) | 1.25 (0.12) | 1.16 (0.02) | 1.2 (0.03) |
| Breakfast | 1.11 (0.04) | 1.11 (0.03) | 1.12 (0.01) | 1.12 (0.01) | 1.15 (0.03) | 1.18 (0.04) | 1.13 (0.02) | 1.15 (0.02) |
| Lunch | 1.11 (0.02) | 1.12 (0.02) | 1.16 (0.02) | 1.14 (0.01) | 1.18 (0.02) | 1.17 (0.03) | 1.13 (0.01) | 1.16 (0.01) |
| Afternoon | 1.13 (0.04) | 1.1 (0.03) | 1.16 (0.03) | 1.15 (0.02) | 1.24 (0.02) | 1.22 (0.03) | 1.26 (0.03) | 1.24 (0.02) |
| Dinner | 1.17 (0.03) | 1.15 (0.03) | 1.24 (0.02) | 1.2 (0.02) | 1.21 (0.03) | 1.22 (0.02) | 1.21 (0.03) | 1.26 (0.02) |
| Evening | 1.17 (0.04) | 1.15 (0.04) | 1.21 (0.03) | 1.19 (0.03) | 1.21 (0.03) | 1.24 (0.03) | 1.18 (0.02) | 1.21 (0.02) |
|  |  |  |  |  |  |  |  |  |
|  |  |  |  |  |  |  |  |  |
| ***Oakland, CA (n=4)*** |  |  |  |  |  |  |  |  |
| Total | 1.19 (0.06) | 1.17 (0.05) | 1.19 (0.04) | 1.16 (0.02) | 1.3 (0.05) | 1.28 (0.04) | 1.29 (0.04) | 1.29 (0.02) |
| *Time of day* |  |  |  |  |  |  |  |  |
| Late night | 1.21 (0.05) | 1.18 (0.04) | 1.21 (0.04) | 1.19 (0.04) | 1.26 (0.04) | 1.29 (0.05) | 1.25 (0.03) | 1.27 (0.03) |
| Breakfast | 1.14 (0.04) | 1.14 (0.05) | 1.14 (0.02) | 1.14 (0.02) | 1.19 (0.04) | 1.19 (0.04) | 1.21 (0.04) | 1.22 (0.03) |
| Lunch | 1.2 (0.24) | 1.14 (0.04) | 1.16 (0.03) | 1.14 (0.02) | 1.23 (0.04) | 1.22 (0.05) | 1.21 (0.02) | 1.23 (0.03) |
| Afternoon | 1.17 (0.15) | 1.19 (0.46) | 1.16 (0.03) | 1.14 (0.02) | 1.37 (0.09) | 1.31 (0.06) | 1.34 (0.04) | 1.31 (0.03) |
| Dinner | 1.21 (0.04) | 1.19 (0.06) | 1.22 (0.02) | 1.2 (0.02) | 1.3 (0.05) | 1.31 (0.05) | 1.31 (0.03) | 1.34 (0.03) |
| Evening | 1.21 (0.03) | 1.19 (0.03) | 1.21 (0.03) | 1.19 (0.02) | 1.3 (0.05) | 1.31 (0.04) | 1.27 (0.03) | 1.3 (0.03) |
|  |  |  |  |  |  |  |  |  |
|  |  |  |  |  |  |  |  |  |
| ***Seattle, WA (n=4)*** |  |  |  |  |  |  |  |  |
| Total | 1.15 (0.07) | 1.15 (0.02) | 1.16 (0.02) | 1.17 (0.02) | 1.2 (0.03) | 1.21 (0.03) | 1.24 (0.03) | 1.25 (0.03) |
| *Time of day* |  |  |  |  |  |  |  |  |
| Late night | 1.16 (0.06) | 1.18 (0.06) | 1.21 (0.03) | 1.21 (0.04) | 1.21 (0.05) | 1.23 (0.05) | 1.26 (0.04) | 1.26 (0.04) |
| Breakfast | 1.12 (0.05) | 1.1 (0.04) | 1.13 (0.02) | 1.13 (0.02) | 1.16 (0.05) | 1.16 (0.05) | 1.19 (0.03) | 1.2 (0.04) |
| Lunch | 1.12 (0.03) | 1.12 (0.02) | 1.14 (0.02) | 1.14 (0.02) | 1.16 (0.02) | 1.17 (0.02) | 1.19 (0.03) | 1.2 (0.03) |
| Afternoon | 1.11 (0.04) | 1.12 (0.03) | 1.15 (0.02) | 1.15 (0.02) | 1.19 (0.05) | 1.22 (0.04) | 1.26 (0.04) | 1.25 (0.03) |
| Dinner | 1.15 (0.04) | 1.16 (0.03) | 1.2 (0.03) | 1.2 (0.03) | 1.21 (0.04) | 1.24 (0.04) | 1.29 (0.04) | 1.3 (0.03) |
| Evening | 1.19 (0.24) | 1.17 (0.04) | 1.19 (0.03) | 1.19 (0.02) | 1.21 (0.05) | 1.23 (0.04) | 1.25 (0.04) | 1.25 (0.03) |
|  |  |  |  |  |  |  |  |  |
| ***Cook County, IL (n=47)*** |  |  |  |  |  |  |  |  |
| Total | 1.13 (0.03) | 1.11 (0.02) | 1.16 (0.04) | 1.16 (0.07) | 1.21 (0.06) | 1.22 (0.05) | 1.24 (0.05) | 1.25 (0.07) |
| *Time of day* |  |  |  |  |  |  |  |  |
| Late night | 1.16 (0.14) | 1.16 (0.12) | 1.23 (0.27) | 1.18 (0.1) | 1.22 (0.1) | 1.24 (0.11) | 1.24 (0.05) | 1.26 (0.05) |
| Breakfast | 1.09 (0.05) | 1.09 (0.05) | 1.12 (0.03) | 1.15 (0.24) | 1.15 (0.06) | 1.16 (0.06) | 1.18 (0.03) | 1.19 (0.04) |
| Lunch | 1.1 (0.04) | 1.09 (0.03) | 1.13 (0.03) | 1.14 (0.1) | 1.16 (0.05) | 1.18 (0.05) | 1.18 (0.03) | 1.19 (0.03) |
| Afternoon | 1.1 (0.04) | 1.09 (0.03) | 1.13 (0.04) | 1.13 (0.04) | 1.23 (0.08) | 1.21 (0.06) | 1.27 (0.06) | 1.24 (0.04) |
| Dinner | 1.15 (0.05) | 1.13 (0.03) | 1.19 (0.03) | 1.17 (0.03) | 1.24 (0.06) | 1.26 (0.06) | 1.28 (0.04) | 1.3 (0.04) |
| Evening | 1.16 (0.05) | 1.14 (0.04) | 1.18 (0.03) | 1.16 (0.03) | 1.23 (0.06) | 1.24 (0.06) | 1.25 (0.05) | 1.26 (0.04) |

SD=standard deviation

^a^Unconditional on a transaction including a beverage item.

^b^Conditional on a transaction including a beverage item.

**Table D.** Difference-in-differences model-based estimates of purchase outcomes after tax implementation, by location and time of day, average effect across months 3-24

|  | *Beverage calories* | *Total calories* | *Beverage sugar (g)* | *Beverage count* | *% transactions with a beverage* |
| --- | --- | --- | --- | --- | --- |
|  | Difference-in-differences, β (95% CI) | Difference-in-differences, β (95% CI) | Difference-in-differences, β (95% CI) | Difference-in-differences, β (95% CI) | Difference-in-differences, β (95% CI) |
| ***UNCONDITIONAL*^a^** |  |  |  |  |  |
| *Philadelphia, PA (n=4)* |  |  |  |  |  |
| Total | -1.20 (-3.88, 1.48) | -28.18 (-40.86, -15.51) | -0.28 (-0.95, 0.38) | -0.01 (-0.02, 0.00) | -0.92 (-1.60, -0.24) |
| *Time of day* |  |  |  |  |  |
| Late night | -2.21 (-7.56, 3.13) | 3.00 (-44.14, 50.14) | -0.54 (-1.88, 0.80) | -0.02 (-0.04, -0.00) | -1.22 (-2.67, 0.23) |
| Breakfast | -6.34 (-9.83, -2.86) | -72.59 (-90.21, -54.97) | -1.54 (-2.40, -0.67) | -0.05 (-0.07, -0.04) | -3.59 (-4.82, -2.37) |
| Lunch | -2.83 (-7.72, 2.06) | -15.45 (-30.33, -0.56) | -0.70 (-1.93, 0.52) | -0.00 (-0.02, 0.01) | -0.00 (-1.30, 1.29) |
| Afternoon | -4.41 (-8.08, -0.75) | -18.91 (-33.75, -4.07) | -1.09 (-1.99, -0.19) | -0.03 (-0.05, -0.02) | -2.51 (-3.49, -1.53) |
| Dinner | -1.52 (-5.56, 2.51) | -24.00 (-41.53, -6.46) | -0.37 (-1.37, 0.64) | -0.02 (-0.03, -0.00) | -0.98 (-1.86, -0.10) |
| Evening | 0.92 (-2.94, 4.78) | -25.88 (-41.60, -10.17) | 0.25 (-0.71, 1.22) | -0.01 (-0.02, 0.01) | -0.47 (-1.31, 0.37) |
|  |  |  |  |  |  |
| *Albany, CA (n=1) ^c^* |  |  |  |  |  |
| Total | 1.94 (0.77, 5.24) | 33.09 (7.29, 42.65) | 0.50 (0.22, 1.30) | 0.02 (0.01, 0.02) | 1.27 (1.02, 1.91) |
| *Time of day* |  |  |  |  |  |
| Late night | -0.34 (-5.74, 8.81) | -28.00 (-95.59, 19.93) | -1.24 (-2.11, 0.79) | 0.06 (0.04, 0.10) | 3.07 (2.55, 4.06) |
| Breakfast | 8.21 (5.86, 8.98) | 33.88 (24.49, 55.88) | 2.72 (1.82, 2.92) | 0.03 (0.02, 0.05) | 2.89 (2.05, 4.02) |
| Lunch | -0.44 (-2.16, 1.32) | 33.17 (4.93, 46.63) | -0.04 (-0.68, 1.24) | 0.02 (0.02, 0.03) | 1.29 (1.11, 1.96) |
| Afternoon | 3.50 (1.96, 4.07) | 27.60 (3.95, 35.90) | -0.33 (-0.95, 1.00) | -0.02 (-0.03, -0.00) | -1.84 (-2.01, -0.51) |
| Dinner | 3.50 (2.83, 4.69) | 41.52 (19.61, 47.55) | 0.35 (-0.07, 1.18) | 0.02 (0.02, 0.03) | 1.77 (1.44, 2.34) |
| Evening | 4.88 (1.95, 8.49) | 5.21 (-17.95, 12.57) | 1.52 (1.20, 2.08) | 0.05 (0.05, 0.06) | 4.06 (3.62, 4.51) |
|  |  |  |  |  |  |
|  |  |  |  |  |  |
| *Oakland, CA (n=4)* |  |  |  |  |  |
| Total | -15.66 (-18.78, -12.53) | -45.71 (-57.99, -33.42) | -3.90 (-4.68, -3.13) | -0.05 (-0.06, -0.04) | -3.33 (-3.99, -2.67) |
| *Time of day* |  |  |  |  |  |
| Late night | -16.64 (-20.84, -12.44) | -73.73 (-102.50, -44.95) | -4.15 (-5.20, -3.10) | -0.05 (-0.07, -0.04) | -3.38 (-4.30, -2.47) |
| Breakfast | -0.96 (-3.55, 1.63) | 0.74 (-14.25, 15.73) | -0.23 (-0.88, 0.42) | -0.00 (-0.01, 0.01) | 0.02 (-0.79, 0.82) |
| Lunch | -9.95 (-13.47, -6.43) | -25.07 (-38.91, -11.22) | -2.46 (-3.34, -1.58) | -0.05 (-0.07, -0.03) | -2.35 (-3.12, -1.58) |
| Afternoon | -24.36 (-29.75, -18.97) | -59.62 (-76.70, -42.53) | -6.07 (-7.39, -4.74) | -0.09 (-0.11, -0.07) | -5.14 (-6.17, -4.11) |
| Dinner | -20.77 (-24.40, -17.14) | -40.07 (-55.07, -25.06) | -5.18 (-6.09, -4.27) | -0.07 (-0.08, -0.06) | -4.72 (-5.40, -4.04) |
| Evening | -16.33 (-20.00, -12.67) | -44.01 (-60.99, -27.04) | -4.07 (-4.99, -3.15) | -0.05 (-0.06, -0.04) | -3.21 (-3.99, -2.43) |
|  |  |  |  |  |  |
|  |  |  |  |  |  |
| *Seattle, WA (n=4)* |  |  |  |  |  |
| Total | 2.80 (0.61, 5.00) | 22.11 (10.45, 33.78) | 0.67 (0.12, 1.21) | 0.02 (0.01, 0.03) | 1.24 (0.66, 1.83) |
| *Time of day* |  |  |  |  |  |
| Late night | 0.97 (-3.96, 5.91) | 38.95 (12.99, 64.90) | 0.23 (-1.00, 1.46) | 0.02 (0.00, 0.04) | 1.32 (0.30, 2.34) |
| Breakfast | -1.03 (-4.87, 2.80) | 10.60 (-3.65, 24.85) | -0.22 (-1.19, 0.74) | -0.00 (-0.02, 0.01) | 0.20 (-0.96, 1.37) |
| Lunch | -0.09 (-2.68, 2.50) | 5.62 (-5.57, 16.82) | -0.05 (-0.69, 0.60) | 0.01 (0.00, 0.02) | 1.30 (0.45, 2.14) |
| Afternoon | 6.25 (3.71, 8.78) | 19.54 (4.09, 34.99) | 1.52 (0.89, 2.15) | 0.03 (0.02, 0.04) | 1.93 (1.07, 2.79) |
| Dinner | 2.47 (-0.60, 5.55) | 34.43 (15.77, 53.08) | 0.59 (-0.18, 1.35) | 0.02 (0.01, 0.03) | 1.06 (0.36, 1.75) |
| Evening | 0.58 (-2.48, 3.64) | 3.48 (-13.55, 20.52) | 0.11 (-0.66, 0.87) | 0.00 (-0.02, 0.03) | 0.87 (0.15, 1.59) |
|  |  |  |  |  |  |
| *Cook County, IL (n=47) ^d^* |  |  |  |  |  |
| Total | -1.79 (-2.59, -0.99) | -13.02 (-17.42, -8.62) | -0.45 (-0.65, -0.26) | 0.00 (-0.00, 0.01) | -0.33 (-0.53, -0.13) |
| *Time of day* |  |  |  |  |  |
| Late night | -3.78 (-5.50, -2.06) | -17.47 (-25.48, -9.46) | -0.94 (-1.37, -0.51) | -0.02 (-0.02, -0.01) | -1.38 (-1.78, -0.98) |
| Breakfast | 1.07 (-0.19, 2.33) | 3.31 (-2.86, 9.48) | 0.28 (-0.04, 0.60) | -0.02 (-0.02, -0.01) | 0.31 (-0.05, 0.67) |
| Lunch | -3.81 (-4.67, -2.95) | -13.13 (-17.61, -8.66) | -0.95 (-1.16, -0.74) | -0.01 (-0.02, -0.01) | -0.72 (-0.94, -0.51) |
| Afternoon | -0.47 (-1.91, 0.97) | -15.54 (-20.97, -10.11) | -0.15 (-0.50, 0.20) | 0.00 (-0.00, 0.01) | -0.10 (-0.44, 0.23) |
| Dinner | -2.17 (-3.11, -1.24) | -14.62 (-19.90, -9.34) | -0.55 (-0.78, -0.32) | -0.00 (-0.01, -0.00) | -0.33 (-0.56, -0.09) |
| Evening | -2.22 (-3.29, -1.16) | -20.07 (-25.64, -14.49) | -0.56 (-0.82, -0.30) | -0.00 (-0.01, -0.00) | -0.31 (-0.57, -0.05) |
|  |  |  |  |  |  |
|  |  |  |  |  |  |
| ***CONDITIONAL*^b^** |  |  |  |  |  |
| *Philadelphia, PA (n=4)* |  |  |  |  |  |
| Total | 3.39 (0.02, 6.77) | -28.18 (-40.86, -15.51) | 0.88 (0.03, 1.72) | -0.00 (-0.01, 0.01) | - |
| *Time of day* |  |  |  |  |  |
| Late night | 0.22 (-9.22, 9.67) | 3.00 (-44.14, 50.14) | 0.08 (-2.28, 2.44) | -0.04 (-0.10, 0.02) | - |
| Breakfast | 4.41 (-0.51, 9.32) | -72.59 (-90.21, -54.97) | 1.17 (-0.08, 2.41) | -0.02 (-0.04, 0.00) | - |
| Lunch | -3.18 (-12.17, 5.81) | -15.45 (-30.33, -0.56) | -0.78 (-3.03, 1.47) | -0.01 (-0.04, 0.02) | - |
| Afternoon | 7.30 (3.52, 11.07) | -18.91 (-33.75, -4.07) | 1.83 (0.89, 2.77) | 0.02 (-0.00, 0.04) | - |
| Dinner | 2.79 (-2.86, 8.44) | -24.00 (-41.53, -6.46) | 0.72 (-0.69, 2.14) | -0.01 (-0.05, 0.02) | - |
| Evening | 5.27 (1.58, 8.97) | -25.88 (-41.60, -10.17) | 1.36 (0.44, 2.28) | 0.01 (-0.01, 0.03) | - |
|  |  |  |  |  |  |
| *Albany, CA (n=1) ^c^* |  |  |  |  |  |
| Total | -3.14 (-7.89, 1.26) | 33.09 (7.29, 42.65) | -0.73 (-1.90, 0.31) | -0.00 (-0.01, 0.00) | - |
| *Time of day* |  |  |  |  |  |
| Late night | -97.83 (-108.35, -64.38) | -28.00 (-95.59, 19.93) | -7.49 (-9.81, -4.57) | 0.08 (-0.01, 0.17) | - |
| Breakfast | 23.21 (15.19, 27.91) | 33.88 (24.49, 55.88) | 3.70 (1.53, 4.63) | 0.01 (-0.01, 0.03) | - |
| Lunch | -8.11 (-13.12, 1.37) | 33.17 (4.93, 46.63) | -1.54 (-2.70, 0.00) | -0.01 (-0.02, -0.01) | - |
| Afternoon | -2.19 (-9.02, 6.07) | 27.60 (3.95, 35.90) | 1.70 (0.33, 2.51) | -0.03 (-0.04, -0.02) | - |
| Dinner | -12.18 (-14.41, -5.29) | 41.52 (19.61, 47.55) | -2.12 (-2.70, -1.14) | -0.02 (-0.03, -0.01) | - |
| Evening | -39.53 (-44.98, -25.87) | 5.21 (-17.95, 12.57) | -3.96 (-4.76, -2.72) | -0.00 (-0.01, 0.02) | - |
|  |  |  |  |  |  |
|  |  |  |  |  |  |
| *Oakland, CA (n=4)* |  |  |  |  |  |
| Total | -8.13 (-11.28, -4.97) | -45.71 (-57.99, -33.42) | -2.05 (-2.84, -1.26) | -0.03 (-0.05, -0.01) | - |
| *Time of day* |  |  |  |  |  |
| Late night | -9.46 (-15.23, -3.68) | -73.73 (-102.50, -44.95) | -2.35 (-3.80, -0.91) | 0.01 (-0.01, 0.03) | - |
| Breakfast | -1.04 (-5.66, 3.58) | 0.74 (-14.25, 15.73) | -0.22 (-1.40, 0.96) | -0.00 (-0.02, 0.02) | - |
| Lunch | -3.84 (-8.22, 0.54) | -25.07 (-38.91, -11.22) | -0.93 (-2.03, 0.16) | -0.06 (-0.13, 0.01) | - |
| Afternoon | -10.61 (-15.23, -6.00) | -59.62 (-76.70, -42.53) | -2.79 (-3.92, -1.66) | -0.01 (-0.11, 0.09) | - |
| Dinner | -7.71 (-12.43, -3.00) | -40.07 (-55.07, -25.06) | -1.94 (-3.12, -0.75) | -0.01 (-0.03, 0.00) | - |
| Evening | -8.97 (-13.16, -4.78) | -44.01 (-60.99, -27.04) | -2.24 (-3.30, -1.18) | -0.01 (-0.02, 0.01) | - |
|  |  |  |  |  |  |
|  |  |  |  |  |  |
| *Seattle, WA (n=4)* |  |  |  |  |  |
| Total | -0.92 (-3.16, 1.31) | 22.11 (10.45, 33.78) | -0.29 (-0.84, 0.27) | -0.01 (-0.03, 0.02) | - |
| *Time of day* |  |  |  |  |  |
| Late night | -4.96 (-12.78, 2.86) | 38.95 (12.99, 64.90) | -1.26 (-3.21, 0.69) | 0.01 (-0.02, 0.04) | - |
| Breakfast | -0.52 (-6.47, 5.43) | 10.60 (-3.65, 24.85) | -0.03 (-1.55, 1.49) | -0.03 (-0.05, -0.01) | - |
| Lunch | -5.69 (-9.15, -2.22) | 5.62 (-5.57, 16.82) | -1.46 (-2.33, -0.60) | 0.00 (-0.01, 0.01) | - |
| Afternoon | 1.46 (-2.10, 5.01) | 19.54 (4.09, 34.99) | 0.31 (-0.55, 1.17) | 0.01 (-0.01, 0.03) | - |
| Dinner | -0.68 (-4.27, 2.92) | 34.43 (15.77, 53.08) | -0.22 (-1.11, 0.67) | 0.02 (0.01, 0.03) | - |
| Evening | -3.34 (-7.41, 0.73) | 3.48 (-13.55, 20.52) | -0.91 (-1.92, 0.10) | -0.04 (-0.14, 0.07) | - |
|  |  |  |  |  |  |
| *Cook County, IL (n=47)^d^* |  |  |  |  |  |
| Total | -1.21 (-1.90, -0.51) | -13.02 (-17.42, -8.62) | -0.31 (-0.49, -0.14) | -0.01 (-0.01, -0.00) | - |
| *Time of day* |  |  |  |  |  |
| Late night | 2.31 (0.16, 4.47) | -17.47 (-25.48, -9.46) | 0.59 (0.05, 1.13) | 0.02 (0.00, 0.03) | - |
| Breakfast | 1.55 (-0.53, 3.62) | 3.31 (-2.86, 9.48) | 0.42 (-0.10, 0.95) | -0.09 (-0.11, -0.07) | - |
| Lunch | -2.69 (-3.64, -1.75) | -13.13 (-17.61, -8.66) | -0.67 (-0.91, -0.43) | -0.03 (-0.03, -0.02) | - |
| Afternoon | -0.33 (-1.45, 0.79) | -15.54 (-20.97, -10.11) | -0.14 (-0.42, 0.13) | 0.01 (-0.01, 0.04) | - |
| Dinner | -1.60 (-2.57, -0.63) | -14.62 (-19.90, -9.34) | -0.41 (-0.65, -0.17) | 0.00 (-0.00, 0.01) | - |
| Evening | -1.85 (-2.99, -0.71) | -20.07 (-25.64, -14.49) | -0.47 (-0.75, -0.19) | 0.00 (-0.01, 0.01) | - |

CI=confidence interval

^a^Unconditional on a transaction including a beverage item.

^b^Conditional on a transaction including a beverage item.

^c^We used a bootstrap method with 100 reiterations to construct 95% confidence intervals for estimates derived from the one restaurant unit located in Albany, CA.

^d^Estimates correspond to an average effect across 5 months, given how the tax was repealed in Cook County, IL.

**Table E.** Difference-in-differences model estimates of beverage calories purchased per transaction, by months open after tax implementation^a^

|  | *Single items* | | | *Combo meals* | | |
| --- | --- | --- | --- | --- | --- | --- |
| Month relative to tax implementation time | Difference-in-differences,  β (95% CI),  **≤12 months** | Difference-in-differences,  β (95% CI),  **≤18 months** | Difference-in-differences,  β (95% CI),  **≤24 months** | Difference-in-differences,  β (95% CI),  **≤12 months** | Difference-in-differences,  β (95% CI),  **≤18 months** | Difference-in-differences,  β (95% CI),  **≤24 months** |
| ***UNCONDITIONAL^a^*** |  |  |  |  |  |  |
| Philadelphia, PA (n=4) | -0.39 (-1.68, 0.90) | -0.02 (-1.23, 1.19) | 0.14 (-0.97, 1.25) | -1.58 (-5.10, 1.94) | -1.90 (-4.76, 0.96) | -1.36 (-4.38, 1.67) |
| Albany, CA (n=1)***^b^*** | 4.39 (3.13, 5.63) | 4.36 (3.52, 5.00) | 3.37 (1.91, 4.79) | -2.11 (-2.89, -0.06) | -2.45 (-3.02, -0.79) | -1.35 (-2.00, 0.54) |
| Oakland, CA (n=4) | 2.65 (1.13, 4.17) | 2.58 (1.36, 3.81) | 1.15 (-0.01, 2.32) | -13.45 (-16.99, -9.92) | -13.24 (-15.94, -10.53) | -16.83 (-19.59, -14.07) |
| Seattle, WA (n=4) | 0.29 (-0.80, 1.39) | 0.35 (-0.60, 1.30) | -0.48 (-1.52, 0.56) | 3.32 (1.58, 5.07) | 2.57 (0.67, 4.47) | 2.99 (1.04, 4.95) |
| Cook County, IL (n=47) | -0.97 (-1.33, -0.60) | -0.69 (-1.06, -0.31) | -0.84 (-1.18, -0.50) | -0.63 (-1.36, 0.09) | -0.13 (-0.81, 0.55) | -0.98 (-1.69, -0.26) |
|  |  |  |  |  |  |  |
| ***CONDITIONAL^c^*** |  |  |  |  |  |  |
| Philadelphia, PA (n=4) | 4.01 (0.58, 7.43) | 2.77 (-0.19, 5.73) | 5.22 (2.36, 8.09) | 1.41 (-4.45, 7.27) | -0.12 (-4.38, 4.14) | 2.19 (-2.15, 6.53) |
| Albany, CA (n=1)***^b^*** | -1.05 (-5.70, 9.76) | -1.50 (-5.04, 6.17) | -7.69 (-13.98, 1.36) | -2.11 (-2.89, -0.06) | -2.45 (-3.02, -0.79) | -1.35 (-2.00, 0.54) |
| Oakland, CA (n=4) | -4.04 (-8.18, 0.10) | -3.43 (-7.18, 0.32) | -8.12 (-11.53, -4.71) | -1.05 (-6.42, 4.32) | 0.49 (-3.87, 4.86) | -1.01 (-4.93, 2.92) |
| Seattle, WA (n=4) | -4.22 (-8.31, -0.13) | -1.97 (-5.30, 1.36) | -5.00 (-8.57, -1.42) | -0.67 (-2.79, 1.44) | -2.65 (-4.62, -0.68) | -0.58 (-2.72, 1.55) |
| Cook County, IL (n=47) | -3.40 (-4.69, -2.12) | -1.98 (-3.15, -0.81) | -2.77 (-3.93, -1.62) | -0.84 (-1.67, -0.01) | -1.28 (-2.07, -0.50) | -1.33 (-2.09, -0.58) |

CI=confidence interval

^a^Unconditional on a transaction including a beverage item.

^b^ We used a bootstrap method with 100 reiterations to construct 95% confidence intervals for estimates derived from the one restaurant unit located in Albany, CA.

^c^Conditional on a transaction including a beverage item.

**Table F.** Difference-in-differences model-based estimates of beverage calories purchased per transaction after tax implementation, 6-month baseline period and 1-month washout periods

|  | *Beverage calories,  single items* | | | *Beverage calories,  combo meals* | | |
| --- | --- | --- | --- | --- | --- | --- |
|  | Difference, soda tax restaurants | Difference, comparison restaurants | Difference-in-differences | Difference, soda tax restaurants | Difference, comparison restaurants | Difference-in-differences |
|  | β (95% CI) | β (95% CI) | β (95% CI) | β (95% CI) | β (95% CI) | β (95% CI) |
| ***UNCONDITIONAL^a^*** |  |  |  |  |  |  |
| **Unmatched** |  |  |  |  |  |  |
| Philadelphia, PA (n=4) | -8.17 (-9.53, -6.82) | -8.81 (-8.85, -8.77) | 0.64 (-0.72, 1.99) | 22.70 (18.71, 26.69) | 19.79 (19.73, 19.86) | 2.91 (-1.08, 6.90) |
| Albany, CA (n=1) | -3.61 (-3.61, -3.61) | -7.72 (-7.76, -7.69) | 4.11 (4.08, 4.15) | 9.99 (9.99, 9.99) | 15.32 (15.26, 15.38) | -5.33 (-5.39, -5.27) |
| Oakland, CA (n=4) | -5.92 (-6.54, -5.29) | -5.71 (-5.75, -5.67) | -0.21 (-0.83, 0.42) | -7.89 (-9.27, -6.51) | 10.21 (10.14, 10.27) | -18.09 (-19.48, -16.71) |
| Seattle, WA (n=4) | 0.25 (-0.56, 1.06) | -0.06 (-0.10, -0.03) | 0.31 (-0.50, 1.12) | 6.02 (4.91, 7.13) | 3.66 (3.60, 3.72) | 2.36 (1.25, 3.47) |
| Cook County, IL (n=47) | -6.41 (-6.69, -6.13) | -5.07 (-5.10, -5.03) | -1.35 (-1.63, -1.06) | 6.91 (6.28, 7.54) | 8.99 (8.93, 9.06) | -2.09 (-2.72, -1.46) |
|  |  |  |  |  |  |  |
| **Matched** |  |  |  |  |  |  |
| Philadelphia, PA (n=4) | -8.50 (-9.36, -7.64) | -8.65 (-9.35, -7.95) | 0.14 (-0.97, 1.25) | 21.88 (19.33, 24.42) | 23.23 (21.59, 24.87) | -1.36 (-4.38, 1.67) |
| Albany, CA (n=1) | -3.61 (-3.61, -3.61) | -6.99 (-8.40, -5.52) | 3.37 (1.91, 4.79) | 9.99 (9.99, 9.99) | 11.34 (9.45, 11.99) | -1.35 (-2.00, 0.54) |
| Oakland, CA (n=4) | -5.92 (-6.93, -4.90) | -7.07 (-7.63, -6.51) | 1.15 (-0.01, 2.32) | -7.89 (-10.14, -5.64) | 8.94 (7.34, 10.54) | -16.83 (-19.59, -14.07) |
| Seattle, WA (n=4) | 0.25 (-0.73, 1.22) | 0.73 (0.37, 1.09) | -0.48 (-1.52, 0.56) | 6.02 (4.67, 7.38) | 3.03 (1.62, 4.44) | 2.99 (1.04, 4.95) |
| Cook County, IL (n=47) | -6.36 (-6.65, -6.08) | -5.52 (-5.70, -5.35) | -0.84 (-1.18, -0.50) | 7.14 (6.51, 7.77) | 8.11 (7.78, 8.44) | -0.98 (-1.69, -0.26) |
|  |  |  |  |  |  |  |
| **Matched, 6 months baseline** |  |  |  |  |  |  |
| Philadelphia, PA (n=4) | -9.11 (-10.28, -7.94) | -8.67 (-9.45, -7.89) | -0.44 (-1.85, 0.97) | 14.93 (11.57, 18.28) | 17.42 (15.51, 19.33) | -2.50 (-6.36, 1.36) |
| Albany, CA (n=1) | -1.52 (-1.52, -1.52) | -3.46 (-5.11, -2.81) | 1.95 (1.29, 3.59) | 5.94 (5.94, 5.94) | 4.95 (4.20, 5.75) | 0.99 (0.20, 1.74) |
| Oakland, CA (n=4) | -1.99 (-3.51, -0.47) | -2.18 (-2.84, -1.51) | 0.19 (-1.47, 1.85) | -10.33 (-12.93, -7.73) | 3.82 (1.90, 5.74) | -14.15 (-17.38, -10.92) |
| Seattle, WA (n=4) | 0.09 (-1.01, 1.18) | 0.97 (0.60, 1.35) | -0.89 (-2.04, 0.27) | 6.18 (4.54, 7.82) | 3.00 (1.54, 4.45) | 3.19 (1.00, 5.38) |
| Cook County, IL (n=47) | -4.06 (-4.46, -3.67) | -2.75 (-2.97, -2.54) | -1.31 (-1.76, -0.86) | 4.74 (3.80, 5.68) | 5.19 (4.75, 5.63) | -0.45 (-1.49, 0.59) |
|  |  |  |  |  |  |  |
| **Matched, 1-month washout period** |  |  |  |  |  |  |
| Philadelphia, PA (n=4) | -8.18 (-9.06, -7.30) | -8.15 (-8.84, -7.47) | -0.03 (-1.15, 1.08) | 19.55 (17.01, 22.08) | 21.21 (19.65, 22.76) | -1.66 (-4.63, 1.31) |
| Albany, CA (n=1) | -2.65 (-2.65, -2.65) | -6.05 (-7.48, -4.68) | 3.40 (2.03, 4.83) | 8.38 (8.38, 8.38) | 9.56 (7.97, 10.20) | -1.18 (-1.82, 0.42) |
| Oakland, CA (n=4) | -5.71 (-6.81, -4.60) | -6.52 (-7.07, -5.97) | 0.82 (-0.42, 2.05) | -8.66 (-11.14, -6.19) | 7.26 (5.76, 8.76) | -15.92 (-18.82, -13.03) |
| Seattle, WA (n=4) | 0.40 (-0.58, 1.38) | 0.93 (0.57, 1.29) | -0.53 (-1.57, 0.51) | 5.82 (4.54, 7.10) | 2.81 (1.50, 4.13) | 3.01 (1.17, 4.84) |
| Cook County, IL (n=47) | -5.89 (-6.17, -5.61) | -4.98 (-5.15, -4.81) | -0.91 (-1.23, -0.58) | 5.92 (5.31, 6.54) | 6.94 (6.63, 7.25) | -1.02 (-1.71, -0.33) |
|  |  |  |  |  |  |  |
| **Matched, 2-month follow-up period data only** |  |  |  |  |  |  |
| Cook County, IL (n=47) | -7.68 (-8.25, -7.12) | -6.95 (-7.35, -6.55) | -0.73 (-1.42, -0.04) | 5.64 (4.41, 6.87) | 7.74 (6.98, 8.50) | -2.10 (-3.55, -0.65) |
| All other locations (n=13) | -8.46 (-9.00, -7.93) | -7.52 (-7.81, -7.22) | -0.95 (-1.56, -0.34) | 5.76 (4.77, 6.75) | 7.36 (6.80, 7.92) | -1.60 (-2.73, -0.46) |
|  |  |  |  |  |  |  |
| **Matched, 24-month follow-up period data** |  |  |  |  |  |  |
| Cook County, IL (n=47) | -6.36 (-6.65, -6.08) | -5.52 (-5.70, -5.35) | -0.84 (-1.18, -0.50) | 7.14 (6.51, 7.77) | 8.11 (7.78, 8.44) | -0.98 (-1.69, -0.26) |
| All other locations (n=13) | -5.40 (-6.26, -4.55) | -5.46 (-6.27, -4.64) | 0.06 (-1.13, 1.24) | 7.28 (5.06, 9.50) | 12.16 (10.51, 13.80) | -4.88 (-7.64, -2.11) |
|  |  |  |  |  |  |  |
| **Matched, by observed pass-through, yes/no** |  |  |  |  |  |  |
| Pass-through (Cook County, Philadelphia) | -6.56 (-6.88, -6.24) | -5.76 (-5.99, -5.52) | -0.81 (-1.20, -0.41) | 8.48 (7.76, 9.21) | 9.21 (8.75, 9.67) | -0.72 (-1.58, 0.13) |
| No pass-through (Albany, Oakland, Seattle) | -3.68 (-4.64, -2.72) | -3.93 (-4.88, -2.98) | 0.25 (-1.10, 1.60) | -0.97 (-2.87, 0.92) | 7.16 (5.59, 8.73) | -8.13 (-10.59, -5.67) |
|  |  |  |  |  |  |  |
| **Matched, taxable beverages only** |  |  |  |  |  |  |
| Philadelphia, PA (n=4) | -8.44 (-9.30, -7.59) | -8.61 (-9.31, -7.91) | 0.17 (-0.94, 1.27) | 22.04 (19.49, 24.59) | 23.29 (21.66, 24.93) | -1.26 (-4.29, 1.77) |
| Albany, CA (n=1) | -3.54 (-3.54, -3.54) | -6.98 (-8.37, -5.51) | 3.44 (1.97, 4.84) | 10.06 (10.06, 10.06) | 11.36 (9.48, 12.01) | -1.30 (-1.95, 0.57) |
| Oakland, CA (n=4) | -5.84 (-6.87, -4.82) | -7.04 (-7.60, -6.47) | 1.19 (0.03, 2.36) | -7.74 (-9.98, -5.50) | 9.00 (7.41, 10.60) | -16.75 (-19.50, -14.00) |
| Seattle, WA (n=4) | 0.28 (-0.69, 1.26) | 0.77 (0.40, 1.14) | -0.49 (-1.52, 0.55) | 6.04 (4.69, 7.40) | 3.08 (1.68, 4.49) | 2.96 (1.01, 4.91) |
| Cook County, IL (n=47) | -6.33 (-6.62, -6.04) | -5.49 (-5.67, -5.32) | -0.84 (-1.18, -0.50) | 7.21 (6.58, 7.84) | 8.17 (7.84, 8.50) | -0.96 (-1.67, -0.25) |
|  |  |  |  |  |  |  |
| ***CONDITIONAL^b^*** |  |  |  |  |  |  |
| **Unmatched** |  |  |  |  |  |  |
| Philadelphia, PA (n=4) | -0.77 (-7.62, 6.08) | -4.44 (-4.55, -4.32) | 3.67 (-3.18, 10.52) | 6.50 (-3.76, 16.76) | 1.48 (1.37, 1.58) | 5.02 (-5.23, 15.28) |
| Albany, CA (n=1) | -11.38 (-11.38, -11.38) | -4.61 (-4.72, -4.50) | -6.77 (-6.88, -6.66) | -3.69 (-3.69, -3.69) | -0.92 (-1.02, -0.81) | -2.77 (-2.88, -2.67) |
| Oakland, CA (n=4) | -13.58 (-15.54, -11.63) | -2.93 (-3.04, -2.82) | -10.65 (-12.61, -8.69) | -0.02 (-2.11, 2.07) | 0.55 (0.45, 0.65) | -0.57 (-2.66, 1.52) |
| Seattle, WA (n=4) | 2.84 (0.21, 5.47) | 3.30 (3.19, 3.40) | -0.45 (-3.09, 2.18) | 3.95 (2.37, 5.53) | 0.99 (0.90, 1.09) | 2.95 (1.37, 4.54) |
| Cook County, IL (n=47) | -6.84 (-7.83, -5.85) | -2.17 (-2.27, -2.06) | -4.67 (-5.67, -3.68) | -1.44 (-2.09, -0.78) | 0.65 (0.55, 0.75) | -2.09 (-2.75, -1.43) |
|  |  |  |  |  |  |  |
| **Matched** |  |  |  |  |  |  |
| Philadelphia, PA (n=4) | -1.65 (-3.89, 0.59) | -6.88 (-8.66, -5.09) | 5.22 (2.36, 8.09) | 5.10 (1.00, 9.20) | 2.91 (1.49, 4.34) | 2.19 (-2.15, 6.53) |
| Albany, CA (n=1) | -11.38 (-11.38, -11.38) | -3.70 (-12.74, 2.60) | -7.69 (-13.98, 1.36) | -3.69 (-3.69, -3.69) | -2.68 (-4.42, 1.89) | -1.01 (-5.58, 0.73) |
| Oakland, CA (n=4) | -13.58 (-16.77, -10.39) | -5.46 (-6.68, -4.24) | -8.12 (-11.53, -4.71) | -0.02 (-3.42, 3.39) | 0.99 (-0.97, 2.94) | -1.01 (-4.93, 2.92) |
| Seattle, WA (n=4) | 2.84 (-0.35, 6.04) | 7.84 (6.23, 9.45) | -5.00 (-8.57, -1.42) | 3.95 (2.08, 5.81) | 4.53 (3.49, 5.57) | -0.58 (-2.72, 1.55) |
| Cook County, IL (n=47) | -6.57 (-7.58, -5.56) | -3.80 (-4.35, -3.24) | -2.77 (-3.93, -1.62) | -1.26 (-1.93, -0.58) | 0.08 (-0.26, 0.41) | -1.33 (-2.09, -0.58) |
|  |  |  |  |  |  |  |
| **Matched, 6 months baseline** |  |  |  |  |  |  |
| Philadelphia, PA (n=4) | -3.54 (-6.43, -0.64) | -8.51 (-10.69, -6.34) | 4.98 (1.35, 8.60) | 9.99 (3.66, 16.33) | 6.76 (5.03, 8.48) | 3.24 (-3.32, 9.80) |
| Albany, CA (n=1) | -13.51 (-13.51, -13.51) | -2.05 (-8.41, 3.25) | -11.47 (-16.77, -5.11) | -4.86 (-4.86, -4.86) | -0.60 (-2.13, 2.08) | -4.26 (-6.94, -2.73) |
| Oakland, CA (n=4) | -9.01 (-13.18, -4.83) | -2.01 (-3.67, -0.35) | -7.00 (-11.49, -2.51) | -0.99 (-4.29, 2.32) | -1.25 (-3.28, 0.77) | 0.27 (-3.61, 4.15) |
| Seattle, WA (n=4) | 5.19 (0.98, 9.41) | 10.02 (8.08, 11.96) | -4.83 (-9.46, -0.19) | 4.90 (2.45, 7.35) | 5.57 (4.20, 6.94) | -0.67 (-3.48, 2.14) |
| Cook County, IL (n=47) | -6.00 (-7.37, -4.62) | -2.26 (-2.98, -1.53) | -3.74 (-5.29, -2.19) | -2.84 (-3.75, -1.92) | -1.70 (-2.16, -1.23) | -1.14 (-2.17, -0.12) |
|  |  |  |  |  |  |  |
| **Matched, 1-month washout period** |  |  |  |  |  |  |
| Philadelphia, PA (n=4) | -1.97 (-4.15, 0.22) | -7.19 (-8.95, -5.43) | 5.23 (2.42, 8.03) | 3.71 (-0.23, 7.65) | 1.97 (0.61, 3.33) | 1.74 (-2.42, 5.91) |
| Albany, CA (n=1) | -9.62 (-9.62, -9.62) | -3.65 (-11.62, 2.69) | -5.98 (-12.31, 2.00) | -3.75 (-3.75, -3.75) | -1.89 (-3.49, 2.59) | -1.86 (-6.34, -0.27) |
| Oakland, CA (n=4) | -13.07 (-16.49, -9.65) | -5.02 (-6.24, -3.80) | -8.05 (-11.67, -4.42) | -0.50 (-4.03, 3.03) | 1.02 (-0.90, 2.93) | -1.52 (-5.54, 2.50) |
| Seattle, WA (n=4) | 2.95 (-0.20, 6.09) | 7.47 (5.96, 8.99) | -4.53 (-8.02, -1.04) | 3.56 (1.69, 5.44) | 4.17 (3.12, 5.23) | -0.61 (-2.76, 1.54) |
| Cook County, IL (n=47) | -6.43 (-7.44, -5.42) | -3.56 (-4.10, -3.02) | -2.88 (-4.02, -1.73) | -1.77 (-2.44, -1.10) | -0.45 (-0.78, -0.12) | -1.33 (-2.07, -0.58) |
|  |  |  |  |  |  |  |
| **Matched, 2-month follow-up period data only** |  |  |  |  |  |  |
| Cook County, IL (n=47) | -9.86 (-11.34, -8.37) | -6.49 (-7.39, -5.59) | -3.36 (-5.10, -1.63) | -0.90 (-1.99, 0.19) | -0.37 (-0.95, 0.20) | -0.53 (-1.76, 0.70) |
| All other locations (n=13) | -11.05 (-12.79, -9.30) | -7.22 (-8.19, -6.26) | -3.82 (-5.82, -1.83) | -1.45 (-2.51, -0.39) | -0.43 (-0.99, 0.12) | -1.02 (-2.21, 0.18) |
|  |  |  |  |  |  |  |
| **Matched, 24-month follow-up period data** |  |  |  |  |  |  |
| Cook County, IL (n=47) | -6.57 (-7.58, -5.56) | -3.80 (-4.35, -3.24) | -2.77 (-3.93, -1.62) | -1.26 (-1.93, -0.58) | 0.08 (-0.26, 0.41) | -1.33 (-2.09, -0.58) |
| All other locations (n=13) | -5.91 (-7.79, -4.03) | -2.37 (-3.63, -1.11) | -3.54 (-5.80, -1.28) | 2.58 (0.42, 4.74) | 2.28 (0.95, 3.60) | 0.30 (-2.23, 2.84) |
|  |  |  |  |  |  |  |
| **Matched, by observed pass-through, yes/no** |  |  |  |  |  |  |
| Pass-through (Cook County, Philadelphia) | -6.13 (-7.09, -5.17) | -4.03 (-4.58, -3.47) | -2.10 (-3.22, -0.99) | -0.69 (-1.46, 0.08) | 0.28 (-0.11, 0.68) | -0.97 (-1.84, -0.11) |
| No pass-through (Albany, Oakland, Seattle) | -8.46 (-10.78, -6.14) | -0.21 (-1.63, 1.21) | -8.25 (-10.97, -5.53) | 1.19 (-1.12, 3.50) | 1.97 (0.40, 3.55) | -0.78 (-3.58, 2.01) |
|  |  |  |  |  |  |  |
| **Matched, taxable beverages only** |  |  |  |  |  |  |
| Philadelphia, PA (n=4) | -1.41 (-3.67, 0.86) | -6.74 (-8.53, -4.96) | 5.34 (2.45, 8.22) | 5.53 (1.39, 9.66) | 3.10 (1.67, 4.54) | 2.43 (-1.95, 6.80) |
| Albany, CA (n=1) | -11.01 (-11.01, -11.01) | -3.70 (-12.70, 2.58) | -7.31 (-13.59, 1.69) | -3.41 (-3.41, -3.41) | -2.59 (-4.30, 2.07) | -0.82 (-5.49, 0.89) |
| Oakland, CA (n=4) | -13.23 (-16.38, -10.08) | -5.32 (-6.55, -4.10) | -7.91 (-11.29, -4.53) | 0.33 (-3.08, 3.74) | 1.17 (-0.78, 3.12) | -0.84 (-4.76, 3.09) |
| Seattle, WA (n=4) | 3.02 (-0.16, 6.20) | 8.02 (6.39, 9.65) | -5.00 (-8.57, -1.42) | 4.02 (2.15, 5.89) | 4.68 (3.65, 5.71) | -0.66 (-2.80, 1.48) |
| Cook County, IL (n=47) | -6.40 (-7.40, -5.39) | -3.66 (-4.22, -3.11) | -2.73 (-3.89, -1.58) | -1.03 (-1.71, -0.36) | 0.25 (-0.09, 0.58) | -1.28 (-2.04, -0.53) |

CI=confidence interval

^a^Unconditional on a transaction including a beverage item.

^b^Conditional on a transaction including a beverage item.

**Table G.** Difference-in-differences model-based estimates of beverage calories purchased per transaction after tax implementation, by location and month

|  | *Philadelphia, PA (n=4)* | | *Albany, CA (n=1)^a^* | | *Oakland, CA (n=4)* | | *Seattle, WA (n=4)* | | *Cook County, IL (n=47)* | |
| --- | --- | --- | --- | --- | --- | --- | --- | --- | --- | --- |
|  | Single items | Combo meals | Single items | Combo meals | Single items | Combo meals | Single items | Combo meals | Single items | Combo meals |
| Month relative to sugary drink tax implementation time | Difference-in-differences,  β (95% CI) | | Difference-in-differences,  β (95% CI) | | Difference-in-differences,  β (95% CI) | | Difference-in-differences,  β (95% CI) | | Difference-in-differences,  β (95% CI) | |
| ***UNCONDITIONAL^b^*** |  |  |  |  |  |  |  |  |  |  |
| 3 | -3.29 (-8.31, 1.72) | 0.95 (-15.99, 17.89) | -1.07 (-2.03, 1.69) | -0.07 (-1.23, 1.89) | 0.34 (-3.32, 4.00) | -9.17 (-16.98, -1.36) | -1.15 (-2.95, 0.65) | 4.32 (-2.15, 10.79) | -0.98 (-2.53, 0.56) | -2.18 (-4.48, 0.13) |
| 4 | -1.69 (-5.69, 2.31) | 0.77 (-8.56, 10.09) | 1.44 (0.33, 3.44) | -2.31 (-3.03, 1.10) | 0.14 (-4.26, 4.54) | -12.54 (-18.87, -6.21) | -3.27 (-6.76, 0.23) | 4.44 (-1.84, 10.73) | -1.01 (-2.55, 0.52) | -2.29 (-4.64, 0.05) |
| 5 | -2.12 (-6.02, 1.78) | -2.72 (-10.33, 4.90) | -1.04 (-1.96, 1.48) | 0.98 (-0.28, 3.29) | -2.25 (-6.45, 1.95) | -12.54 (-22.45, -2.63) | -2.21 (-6.46, 2.03) | 6.68 (0.48, 12.88) | -1.46 (-2.99, 0.06) | -3.75 (-6.14, -1.36) |
| 6 | -2.91 (-7.29, 1.47) | -3.28 (-15.72, 9.15) | 5.24 (3.22, 6.70) | -6.11 (-7.49, -1.91) | -1.63 (-5.52, 2.26) | -7.71 (-14.30, -1.12) | -2.19 (-5.43, 1.06) | 4.43 (-2.28, 11.14) | -0.86 (-2.41, 0.68) | -1.46 (-3.83, 0.92) |
| 7 | -0.69 (-4.76, 3.39) | -2.00 (-10.41, 6.42) | 5.71 (3.52, 6.63) | -4.08 (-6.01, 2.24) | -1.95 (-6.17, 2.28) | -6.91 (-14.98, 1.16) | -0.90 (-3.15, 1.34) | 3.36 (-2.41, 9.14) | -1.58 (-3.07, -0.09) | -1.63 (-3.86, 0.61) |
| 8 | 1.12 (-2.64, 4.88) | 0.82 (-7.17, 8.80) | 8.21 (4.87, 9.96) | -4.96 (-6.07, -1.78) | 0.12 (-3.30, 3.55) | -8.00 (-16.62, 0.62) | -0.46 (-3.83, 2.90) | 5.60 (-1.38, 12.58) | -1.17 (-2.68, 0.34) | -1.90 (-4.24, 0.43) |
| 9 | 1.27 (-2.98, 5.53) | -0.84 (-10.65, 8.97) | 7.79 (4.79, 9.18) | 1.05 (-0.97, 1.55) | -2.20 (-5.60, 1.21) | -8.14 (-16.03, -0.24) | -0.44 (-2.66, 1.78) | 0.73 (-6.25, 7.71) | -1.20 (-2.65, 0.26) | -1.49 (-3.99, 1.01) |
| 10 | -0.42 (-4.56, 3.73) | 4.01 (-5.15, 13.16) | 3.31 (1.39, 3.44) | -1.00 (-3.42, 1.32) | -4.45 (-8.76, -0.15) | -9.56 (-18.56, -0.56) | -1.57 (-6.00, 2.86) | 3.43 (-3.83, 10.69) | -1.10 (-2.53, 0.33) | -0.63 (-2.99, 1.73) |
| 11 | 0.27 (-4.54, 5.07) | 1.82 (-6.41, 10.04) | 6.69 (5.92, 8.48) | 2.26 (0.06, 4.29) | -4.29 (-8.89, 0.31) | -8.07 (-16.16, 0.03) | 2.18 (-1.41, 5.77) | 2.00 (-4.71, 8.70) | -0.69 (-2.19, 0.81) | -0.27 (-2.81, 2.27) |
| 12 | 1.94 (-3.02, 6.90) | 3.37 (-5.19, 11.93) | 7.61 (6.64, 9.20) | -7.01 (-8.08, -5.41) | -0.67 (-10.00, 8.67) | 1.39 (-21.27, 24.04) | 1.28 (-1.30, 3.86) | 4.95 (-0.83, 10.72) | -1.66 (-3.16, -0.16) | -2.31 (-5.00, 0.38) |
| 13 | 1.15 (-3.57, 5.88) | -1.09 (-8.83, 6.65) | 4.10 (3.62, 5.72) | -1.18 (-1.74, 0.91) | -4.38 (-8.35, -0.42) | -10.22 (-18.91, -1.53) | -0.92 (-6.26, 4.42) | 6.92 (-0.81, 14.64) | -1.48 (-2.91, -0.04) | -1.04 (-3.50, 1.43) |
| 14 | 0.90 (-3.28, 5.07) | -4.47 (-13.21, 4.26) | 2.38 (1.50, 5.60) | -4.27 (-5.48, -2.36) | -3.05 (-7.09, 0.98) | -14.97 (-22.00, -7.94) | -1.43 (-3.80, 0.95) | 3.25 (-4.30, 10.80) | -1.20 (-2.68, 0.29) | -1.09 (-3.60, 1.41) |
| 15 | -1.69 (-5.50, 2.12) | -0.21 (-7.73, 7.30) | 1.47 (0.79, 2.52) | -1.15 (-2.34, 2.01) | -4.80 (-8.80, -0.81) | -18.18 (-25.66, -10.71) | -2.02 (-4.57, 0.53) | 4.54 (-3.45, 12.54) | -1.15 (-2.59, 0.30) | -2.34 (-4.77, 0.09) |
| 16 | -0.97 (-5.28, 3.35) | -2.30 (-10.44, 5.85) | 2.52 (0.05, 3.46) | -0.67 (-1.92, 0.85) | -1.85 (-5.43, 1.73) | -16.88 (-24.29, -9.46) | -0.61 (-2.32, 1.10) | 7.15 (0.24, 14.06) | -1.44 (-2.98, 0.10) | -3.32 (-5.76, -0.89) |
| 17 | 0.45 (-3.62, 4.52) | 3.77 (-5.30, 12.84) | -0.82 (-2.40, 0.57) | -5.63 (-7.05, -4.85) | -4.18 (-7.95, -0.40) | -15.72 (-22.82, -8.61) | -2.45 (-4.14, -0.75) | 5.94 (0.10, 11.78) | -1.36 (-2.93, 0.21) | -1.53 (-3.84, 0.79) |
| 18 | 0.47 (-3.60, 4.53) | 0.57 (-9.08, 10.23) | 4.32 (1.27, 8.77) | 0.23 (-0.93, 2.57) | -3.42 (-7.01, 0.17) | -16.81 (-23.48, -10.15) | -1.82 (-3.56, -0.09) | 3.46 (-3.62, 10.55) | -1.23 (-2.79, 0.33) | -1.11 (-3.89, 1.68) |
| 19 | 1.10 (-2.96, 5.17) | 1.08 (-11.63, 13.80) | 3.99 (2.59, 4.99) | -3.91 (-5.03, -1.35) | -3.73 (-8.09, 0.63) | -10.91 (-17.62, -4.19) | -3.86 (-5.67, -2.05) | 3.54 (-3.56, 10.64) | -1.13 (-2.72, 0.45) | -2.45 (-5.13, 0.23) |
| 20 | 2.44 (-1.45, 6.33) | 2.26 (-11.29, 15.80) | 5.42 (2.62, 7.67) | -6.28 (-8.12, -2.90) | -5.33 (-9.95, -0.71) | -14.79 (-22.89, -6.69) | -3.92 (-7.44, -0.40) | 3.67 (-4.25, 11.59) | -1.08 (-2.63, 0.47) | -3.38 (-5.88, -0.87) |
| 21 | 0.89 (-3.34, 5.12) | 3.45 (-7.10, 14.00) | 4.46 (2.08, 4.98) | 1.73 (-0.56, 5.07) | -5.64 (-8.96, -2.32) | -9.37 (-17.51, -1.24) | -1.88 (-8.40, 4.63) | 2.35 (-4.51, 9.21) | -0.33 (-1.90, 1.24) | -3.79 (-6.91, -0.68) |
| 22 | -0.24 (-4.80, 4.31) | 0.51 (-9.73, 10.74) | 2.34 (1.20, 3.25) | 2.39 (-0.10, 5.25) | -7.88 (-11.13, -4.62) | -11.42 (-21.48, -1.36) | -1.22 (-3.33, 0.89) | 1.24 (-6.15, 8.63) | 0.37 (-1.32, 2.07) | -3.00 (-6.21, 0.21) |
| 23 | 0.05 (-4.52, 4.61) | 1.17 (-6.77, 9.11) | 2.20 (-0.15, 4.36) | 3.62 (2.42, 5.19) | -6.49 (-10.53, -2.46) | -14.30 (-23.72, -4.89) | -5.15 (-6.94, -3.37) | 1.92 (-6.09, 9.93) | -0.29 (-1.94, 1.36) | -2.80 (-6.02, 0.41) |
| 24 | -0.63 (-4.90, 3.64) | 3.58 (-3.85, 11.00) | -2.09 (-3.11, 0.26) | 6.46 (3.62, 8.08) | -2.37 (-6.74, 2.00) | -18.23 (-28.52, -7.95) | -2.27 (-4.00, -0.54) | -3.37 (-12.20, 5.46) | -0.97 (-2.70, 0.76) | -2.90 (-6.35, 0.56) |
|  |  |  |  |  |  |  |  |  |  |  |
| ***CONDITIONAL^c^*** |  |  |  |  |  |  |  |  |  |  |
| 3 | 1.24 (-7.29, 9.77) | 6.27 (-4.21, 16.76) | -10.74 (-13.10, -1.39) | -3.30 (-6.12, -2.03) | -7.41 (-24.88, 10.06) | -3.51 (-15.86, 8.84) | -11.47 (-22.04, -0.90) | 7.01 (0.70, 13.32) | -3.84 (-8.22, 0.54) | -0.29 (-3.12, 2.53) |
| 4 | 3.96 (-4.53, 12.44) | 13.16 (5.17, 21.15) | 3.61 (0.47, 14.76) | -2.28 (-7.39, 1.55) | -2.35 (-17.88, 13.19) | -2.60 (-14.74, 9.53) | -8.69 (-21.79, 4.41) | 3.96 (-2.08, 10.00) | -5.42 (-10.00, -0.84) | 0.53 (-2.30, 3.35) |
| 5 | -0.44 (-7.62, 6.74) | 8.01 (-2.21, 18.22) | -2.69 (-8.85, 13.31) | -2.38 (-7.61, 2.56) | -5.56 (-19.70, 8.58) | -2.96 (-17.33, 11.41) | -6.40 (-18.50, 5.70) | 4.20 (-3.43, 11.82) | -5.82 (-10.22, -1.42) | -0.77 (-3.64, 2.09) |
| 6 | 3.92 (-9.03, 16.86) | -5.14 (-42.08, 31.79) | -3.05 (-7.68, 12.81) | 2.66 (-0.37, 3.33) | -11.19 (-24.11, 1.72) | -6.27 (-20.04, 7.50) | -8.17 (-19.39, 3.06) | 2.65 (-5.20, 10.49) | -2.08 (-7.04, 2.88) | -0.24 (-3.26, 2.77) |
| 7 | 7.40 (-5.06, 19.86) | 3.20 (-14.46, 20.86) | 3.37 (-1.60, 12.93) | -4.89 (-6.96, 0.25) | -3.38 (-20.81, 14.05) | -7.00 (-20.22, 6.21) | -11.05 (-22.55, 0.45) | 4.94 (-2.39, 12.26) | -5.00 (-9.65, -0.34) | 0.26 (-2.61, 3.13) |
| 8 | 8.90 (-0.27, 18.07) | 17.51 (4.84, 30.19) | 5.78 (-4.44, 16.48) | -6.34 (-11.43, -2.38) | -7.36 (-24.41, 9.70) | -8.25 (-26.36, 9.86) | -5.14 (-16.93, 6.64) | 7.65 (-0.43, 15.74) | -4.90 (-8.99, -0.80) | -0.08 (-2.99, 2.83) |
| 9 | 7.28 (-6.29, 20.85) | 6.77 (-4.59, 18.13) | 1.22 (-6.67, 14.05) | 1.06 (-9.51, 3.92) | -7.70 (-22.64, 7.24) | -7.01 (-23.14, 9.12) | -6.79 (-19.27, 5.70) | 4.54 (-3.57, 12.64) | -3.51 (-7.76, 0.74) | 0.33 (-2.63, 3.29) |
| 10 | 10.26 (1.73, 18.79) | 5.94 (-4.17, 16.06) | -4.54 (-6.76, -1.28) | -3.72 (-12.21, -1.59) | -17.60 (-34.51, -0.69) | -7.05 (-19.37, 5.26) | -10.60 (-33.49, 12.30) | 3.67 (-3.57, 10.92) | -3.86 (-8.04, 0.31) | 1.24 (-1.50, 3.97) |
| 11 | 5.89 (-3.01, 14.79) | 6.85 (-4.37, 18.08) | -3.69 (-6.85, 5.32) | 1.28 (-0.17, 3.56) | -15.63 (-29.24, -2.02) | -9.95 (-25.28, 5.37) | -5.37 (-21.87, 11.14) | 1.58 (-5.80, 8.96) | -4.10 (-8.58, 0.39) | 2.02 (-1.03, 5.07) |
| 12 | 9.77 (-0.65, 20.18) | 10.11 (1.05, 19.17) | 0.34 (-2.85, 9.98) | 2.47 (-3.39, 4.30) | -12.48 (-29.01, 4.04) | 7.61 (-29.44, 44.67) | -14.41 (-29.76, 0.93) | 6.19 (0.68, 11.71) | -7.43 (-12.22, -2.64) | -2.92 (-5.75, -0.09) |
| 13 | 6.13 (-2.55, 14.82) | 6.83 (-2.74, 16.39) | -1.37 (-5.86, 4.93) | 5.96 (3.53, 9.02) | -14.32 (-28.52, -0.12) | -4.86 (-22.71, 12.98) | -10.05 (-19.55, -0.55) | -1.89 (-7.42, 3.64) | -5.32 (-9.84, -0.79) | -2.50 (-5.40, 0.40) |
| 14 | 11.73 (1.62, 21.84) | 4.98 (-5.12, 15.08) | -7.27 (-14.40, 2.62) | -7.18 (-11.12, -3.07) | -10.56 (-26.70, 5.58) | -1.75 (-14.54, 11.04) | -12.84 (-26.00, 0.33) | 0.92 (-6.57, 8.42) | -7.40 (-11.74, -3.07) | -3.61 (-6.49, -0.73) |
| 15 | 5.07 (-4.53, 14.67) | 4.39 (-5.95, 14.74) | -9.95 (-16.20, -1.40) | -2.94 (-6.86, 0.71) | -17.83 (-33.40, -2.26) | 0.09 (-14.75, 14.93) | -10.02 (-20.11, 0.06) | -2.64 (-13.48, 8.20) | -5.60 (-9.94, -1.26) | -3.96 (-6.80, -1.11) |
| 16 | 5.41 (-5.48, 16.30) | 6.85 (-1.08, 14.79) | -14.69 (-24.04, -8.18) | -4.15 (-11.08, -2.98) | -14.66 (-28.65, -0.68) | -3.56 (-16.85, 9.73) | -3.47 (-15.05, 8.12) | 7.42 (-2.05, 16.89) | -1.78 (-6.40, 2.85) | -2.51 (-5.45, 0.43) |
| 17 | 7.29 (-1.25, 15.82) | 17.53 (7.44, 27.62) | -22.56 (-27.34, -12.58) | -2.38 (-13.51, 1.50) | -21.75 (-36.07, -7.42) | -5.42 (-18.36, 7.52) | -8.63 (-19.74, 2.49) | 6.09 (-3.37, 15.55) | -4.31 (-9.07, 0.45) | 1.03 (-2.13, 4.19) |
| 18 | 6.94 (-3.91, 17.79) | 12.87 (2.74, 23.00) | -16.34 (-27.63, 0.76) | 0.14 (-8.75, 5.67) | -16.18 (-30.39, -1.98) | -7.04 (-19.52, 5.45) | -11.24 (-21.81, -0.67) | 2.30 (-11.24, 15.84) | 0.42 (-4.87, 5.70) | 1.74 (-1.91, 5.39) |
| 19 | 12.79 (3.12, 22.46) | 10.13 (-0.22, 20.49) | -11.67 (-21.65, -3.15) | 0.08 (-7.68, 5.45) | -23.19 (-37.31, -9.08) | -9.85 (-22.28, 2.59) | -14.81 (-28.14, -1.47) | 1.46 (-10.34, 13.27) | -2.65 (-7.37, 2.08) | -0.93 (-4.09, 2.23) |
| 20 | 15.89 (6.88, 24.89) | 8.26 (-3.88, 20.41) | -8.44 (-20.32, 5.84) | 1.27 (-6.46, 2.95) | -23.67 (-39.02, -8.31) | -4.16 (-16.37, 8.05) | -19.64 (-32.73, -6.55) | 7.76 (-2.69, 18.21) | -2.22 (-7.14, 2.69) | 0.96 (-2.28, 4.21) |
| 21 | 8.08 (-1.77, 17.92) | 8.11 (-1.41, 17.64) | -16.65 (-26.48, -8.87) | 2.60 (-5.45, 5.92) | -20.47 (-35.52, -5.41) | -4.64 (-17.39, 8.12) | -13.53 (-27.27, 0.21) | 10.78 (5.41, 16.15) | -4.11 (-8.65, 0.43) | -0.12 (-3.00, 2.75) |
| 22 | 4.42 (-6.23, 15.08) | 3.83 (-6.66, 14.33) | -17.90 (-26.16, -13.14) | -0.27 (-5.50, 2.06) | -17.01 (-31.99, -2.04) | -2.13 (-15.72, 11.45) | -4.82 (-13.91, 4.27) | 8.83 (0.63, 17.03) | -2.34 (-7.64, 2.95) | 0.48 (-2.47, 3.43) |
| 23 | 6.44 (-1.19, 14.08) | 8.84 (-1.29, 18.98) | -17.00 (-26.39, -11.83) | 4.49 (-4.29, 5.99) | -12.17 (-28.44, 4.11) | -8.85 (-22.22, 4.52) | -8.26 (-21.42, 4.90) | 4.54 (-0.54, 9.61) | -4.10 (-9.46, 1.25) | -0.46 (-3.70, 2.78) |
| 24 | 6.39 (-2.85, 15.64) | 11.68 (0.23, 23.13) | -14.95 (-20.45, -9.70) | -4.58 (-11.56, -3.07) | -6.76 (-20.40, 6.89) | -3.31 (-19.06, 12.45) | -5.50 (-23.83, 12.84) | 12.18 (4.38, 19.99) | -2.13 (-6.91, 2.66) | -1.14 (-4.55, 2.27) |

CI=confidence interval

^a^The absence of a confidence interval for Albany, CA estimates is due to the sample size of one restaurant.

^b^Unconditional on a transaction including a beverage item.

^c^Conditional on a transaction including a beverage item.

**Table H.** Difference-in-differences model-based estimates of beverage calories purchased per transaction after tax implementation, by restaurant location

|  | Single items | Combo meals |
| --- | --- | --- |
| Restaurant location | Difference-in-differences,  β | |
| ***UNCONDITIONAL*** |  |  |
| Philadelphia |  |  |
| 1 | 9.67 | -39.11 |
| 2 | 6.31 | -15.43 |
| 3 | 11.75 | -28.49 |
| 4 | 7.51 | -8.55 |
| Albany |  |  |
| 5 | 11.57 | -13.11 |
| Oakland |  |  |
| 6 | 7.18 | -32.10 |
| 7 | 8.63 | -17.78 |
| 8 | 4.23 | -12.92 |
| 9 | 16.07 | -46.97 |
| Seattle |  |  |
| 10 | -0.87 | 6.25 |
| 11 | 0.64 | -7.03 |
| 12 | 2.00 | -1.57 |
| 13 | -2.75 | -4.78 |
| Cook County |  |  |
| 14 | 3.71 | -2.96 |
| 15 | 4.83 | -13.73 |
| 16 | 4.90 | -10.36 |
| 17 | 6.50 | -15.44 |
| 18 | 3.79 | -11.26 |
| 19 | -0.27 | 3.62 |
| 20 | 5.18 | -16.72 |
| 21 | 4.38 | -7.36 |
| 22 | 5.90 | -15.03 |
| 23 | 5.20 | -3.06 |
| 24 | 8.70 | -14.76 |
| 25 | 1.29 | -7.21 |
| 26 | 5.62 | -9.89 |
| 27 | 0.34 | -0.54 |
| 28 | 2.70 | -2.41 |
| 29 | 3.79 | -10.21 |
| 30 | 7.43 | -16.34 |
| 31 | 5.94 | -9.42 |
| 32 | 5.59 | 0.66 |
| 33 | 6.79 | -9.00 |
| 34 | 4.03 | -8.44 |
| 35 | 5.27 | -17.07 |
| 36 | 13.86 | -15.70 |
| 37 | 6.94 | 8.48 |
| 38 | 5.34 | -10.05 |
| 39 | 3.09 | -0.48 |
| 40 | 5.30 | -14.57 |
| 41 | 6.40 | -8.18 |
| 42 | 7.20 | -8.88 |
| 43 | 15.12 | -13.42 |
| 44 | 6.06 | -7.87 |
| 45 | -3.10 | -7.21 |
| 46 | -4.35 | -1.36 |
| 47 | 9.41 | -12.58 |
| 48 | 6.48 | -9.36 |
| 49 | 4.45 | 4.73 |
| 50 | 3.31 | -2.33 |
| 51 | 6.26 | -21.39 |
| 52 | -3.63 | 0.08 |
| 53 | 7.77 | -7.97 |
| 54 | 0.28 | -10.90 |
| 55 | 6.75 | -13.52 |
| 56 | 3.97 | -1.35 |
| 57 | 6.38 | -16.33 |
| 58 | 4.79 | -3.48 |
| 59 | 2.69 | -17.91 |
| 60 | 11.16 | -9.68 |
|  |  |  |
| ***CONDITIONAL*** |  |  |
| Philadelphia |  |  |
| 1 | 12.07 | 12.07 |
| 2 | 4.73 | 4.73 |
| 3 | 26.09 | 26.09 |
| 4 | -7.48 | -7.48 |
| Albany |  |  |
| 5 | -2.88 | -2.88 |
| Oakland |  |  |
| 6 | -6.91 | -6.91 |
| 7 | 8.47 | 8.47 |
| 8 | -6.90 | -6.90 |
| 9 | -3.82 | -3.82 |
| Seattle |  |  |
| 10 | -12.89 | -12.89 |
| 11 | 3.89 | 3.89 |
| 12 | -14.47 | -14.47 |
| 13 | -18.82 | -18.82 |
| Cook County |  |  |
| 14 | -5.19 | -5.19 |
| 15 | -3.45 | -3.45 |
| 16 | 4.19 | 4.19 |
| 17 | 5.10 | 5.10 |
| 18 | -2.18 | -2.18 |
| 19 | 1.49 | 1.49 |
| 20 | -2.59 | -2.59 |
| 21 | 12.90 | 12.90 |
| 22 | 1.30 | 1.30 |
| 23 | 22.87 | 22.87 |
| 24 | 8.46 | 8.46 |
| 25 | 3.04 | 3.04 |
| 26 | 5.17 | 5.17 |
| 27 | -1.08 | -1.08 |
| 28 | 4.90 | 4.90 |
| 29 | -5.84 | -5.84 |
| 30 | 1.64 | 1.64 |
| 31 | -4.11 | -4.11 |
| 32 | -14.23 | -14.23 |
| 33 | 12.41 | 12.41 |
| 34 | -6.32 | -6.32 |
| 35 | 7.40 | 7.40 |
| 36 | 17.77 | 17.77 |
| 37 | 31.28 | 31.28 |
| 38 | 3.51 | 3.51 |
| 39 | -10.96 | -10.96 |
| 40 | -2.00 | -2.00 |
| 41 | -3.79 | -3.79 |
| 42 | -10.49 | -10.49 |
| 43 | 12.93 | 12.93 |
| 44 | 30.39 | 30.39 |
| 45 | -8.25 | -8.25 |
| 46 | -17.84 | -17.84 |
| 47 | 1.71 | 1.71 |
| 48 | 0.04 | 0.04 |
| 49 | 3.35 | 3.35 |
| 50 | 6.53 | 6.53 |
| 51 | -3.63 | -3.63 |
| 52 | -4.59 | -4.59 |
| 53 | 12.50 | 12.50 |
| 54 | 13.00 | 13.00 |
| 55 | -1.83 | -1.83 |
| 56 | -2.21 | -2.21 |
| 57 | 1.01 | 1.01 |
| 58 | -21.64 | -21.64 |
| 59 | -2.72 | -2.72 |
| 60 | -0.06 | -0.06 |

**Figure A.** Map^a,b,c^ of the locations of the restaurants in the tax group and comparison group in the final sample


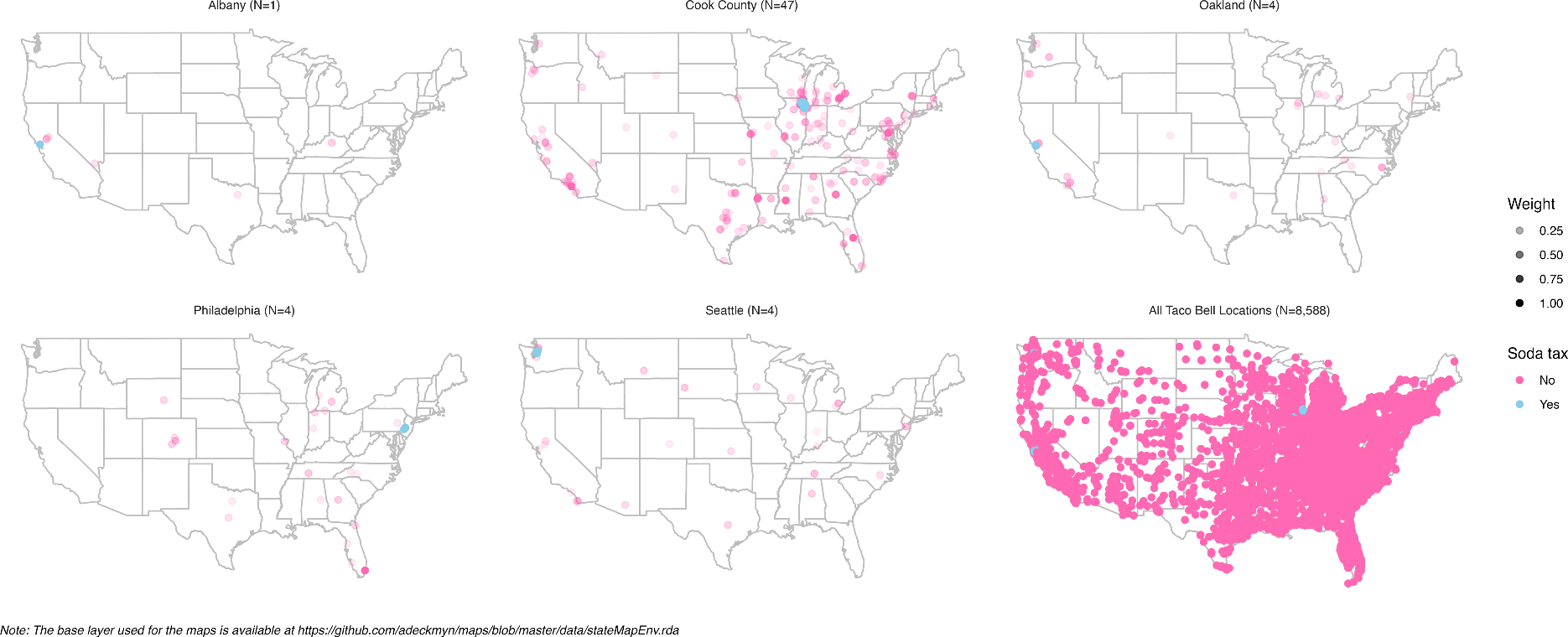


^a^The base map layer data: <https://github.com/adeckmyn/maps/blob/master/data/stateMapEnv.rda>

^b^The license for the library: <https://cran.r-project.org/web/licenses/GPL-2>

^c^Official documentation for the library: <https://cran.r-project.org/web/packages/maps/maps.pdf>

**Figure B.** Difference-in-differences model estimates^a^ of beverage calories purchased per transaction, by location, individual items, conditional^b^


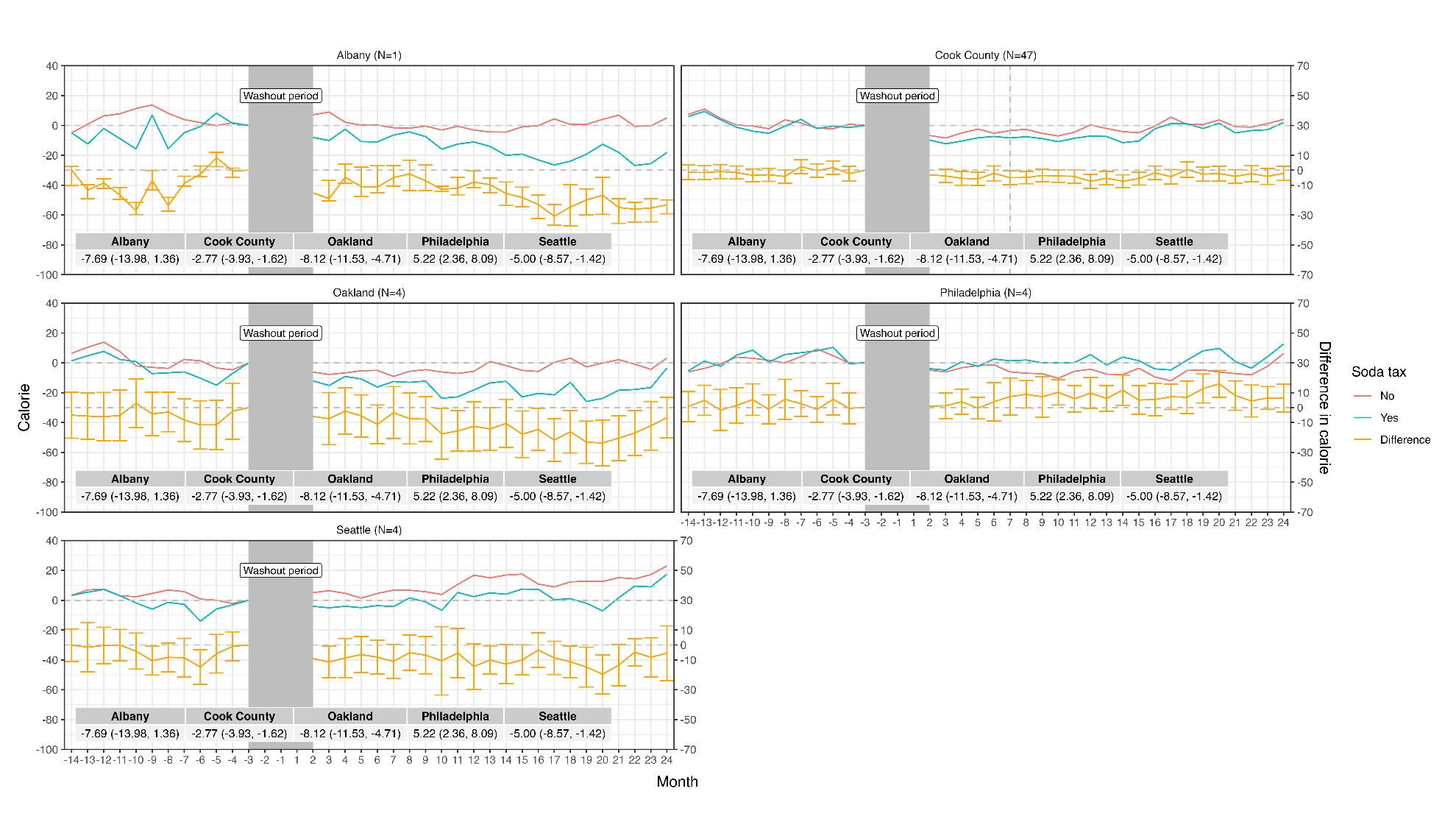


^a^Estimates in legends represent difference-in-differences estimates with 95% confidence intervals

^b^Conditional on a transaction including a beverage item.

**Figure C.** Difference-in-differences model estimates^a^ of beverage calories purchased per transaction, by location, combo meals, conditional^b^


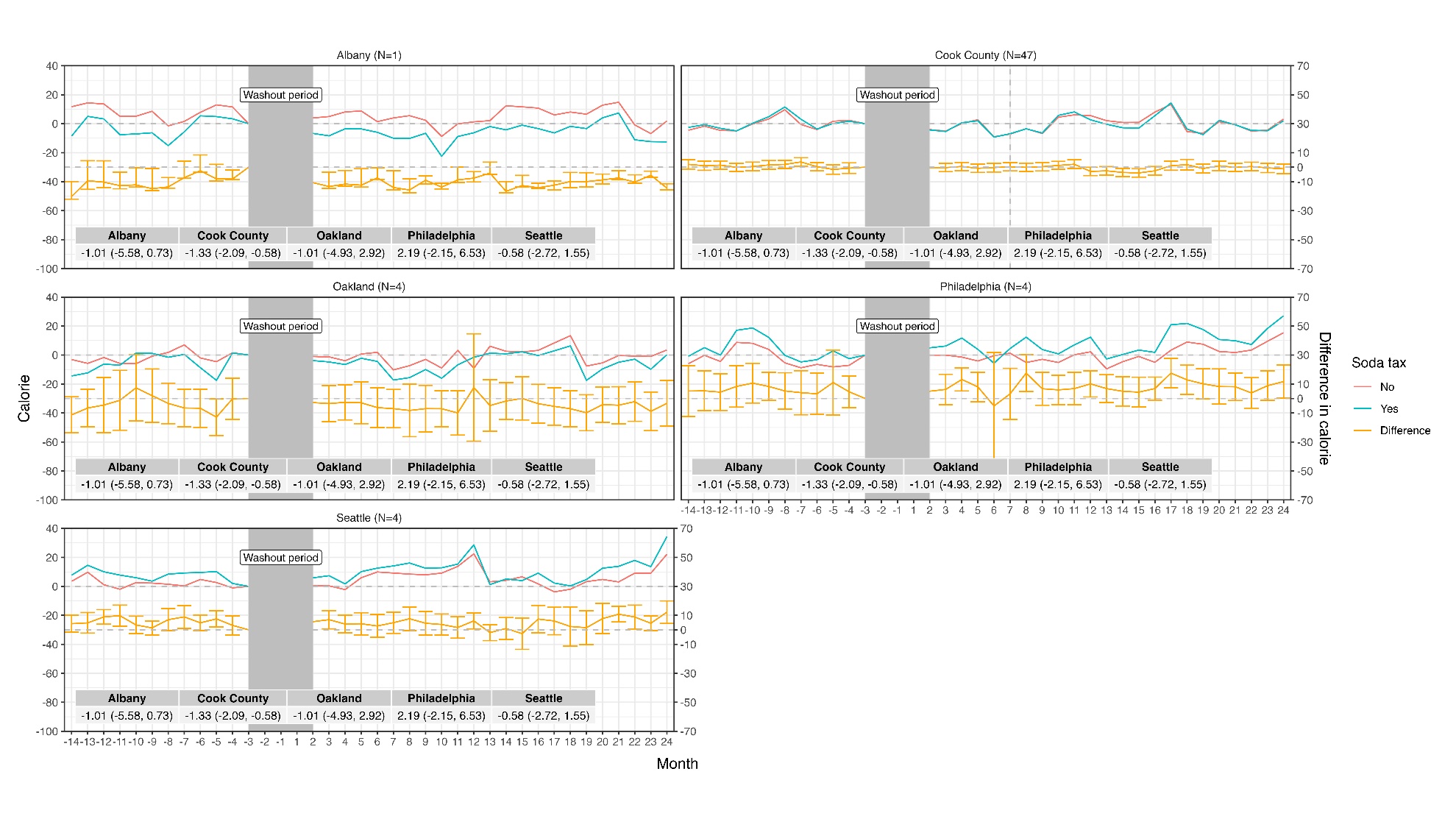


^a^Estimates in legends represent difference-in-differences estimates with 95% confidence intervals

^b^Conditional on a transaction including a beverage item.

**Figure D.1.** Percentage sales of individual items by location and time of day, tax group and comparison group


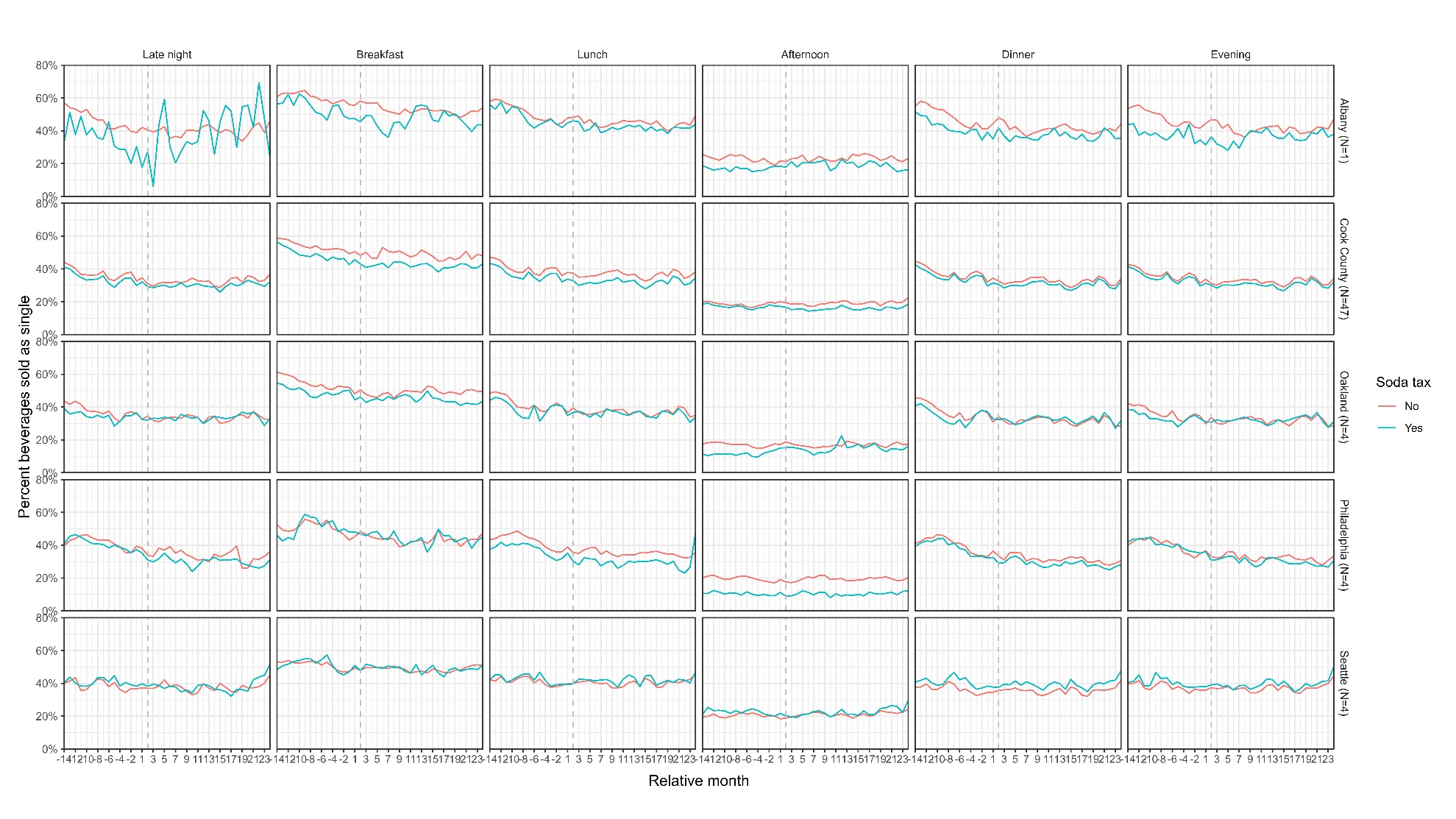


**Figure D.2.** Percentage sales of combo meals, by location and time of day, tax group and comparison group

**
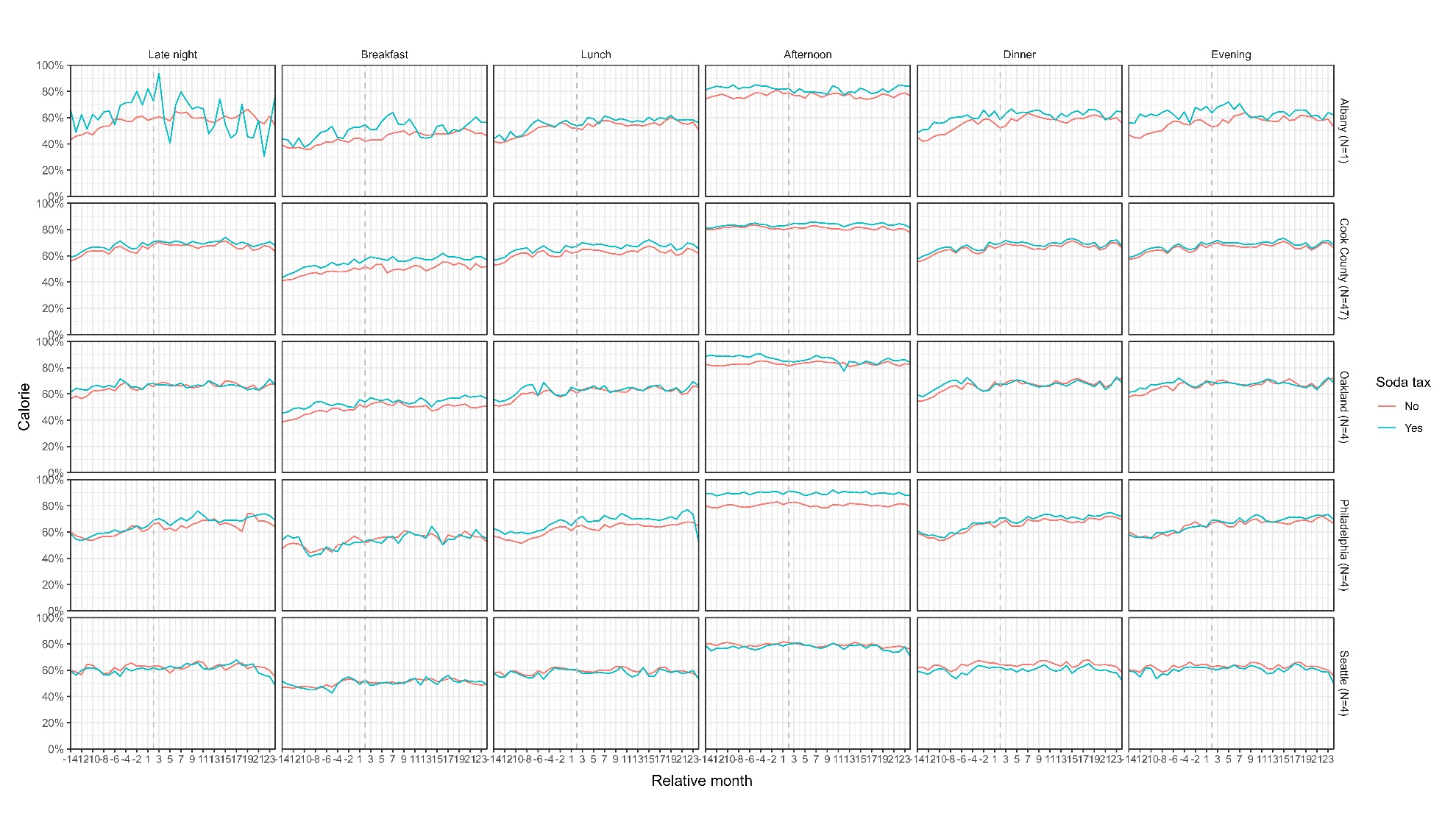
**

**Figure E.1.** Difference-in-differences model estimates of beverage calories purchased per transaction, individual items, by location and time of day, unconditional^a^


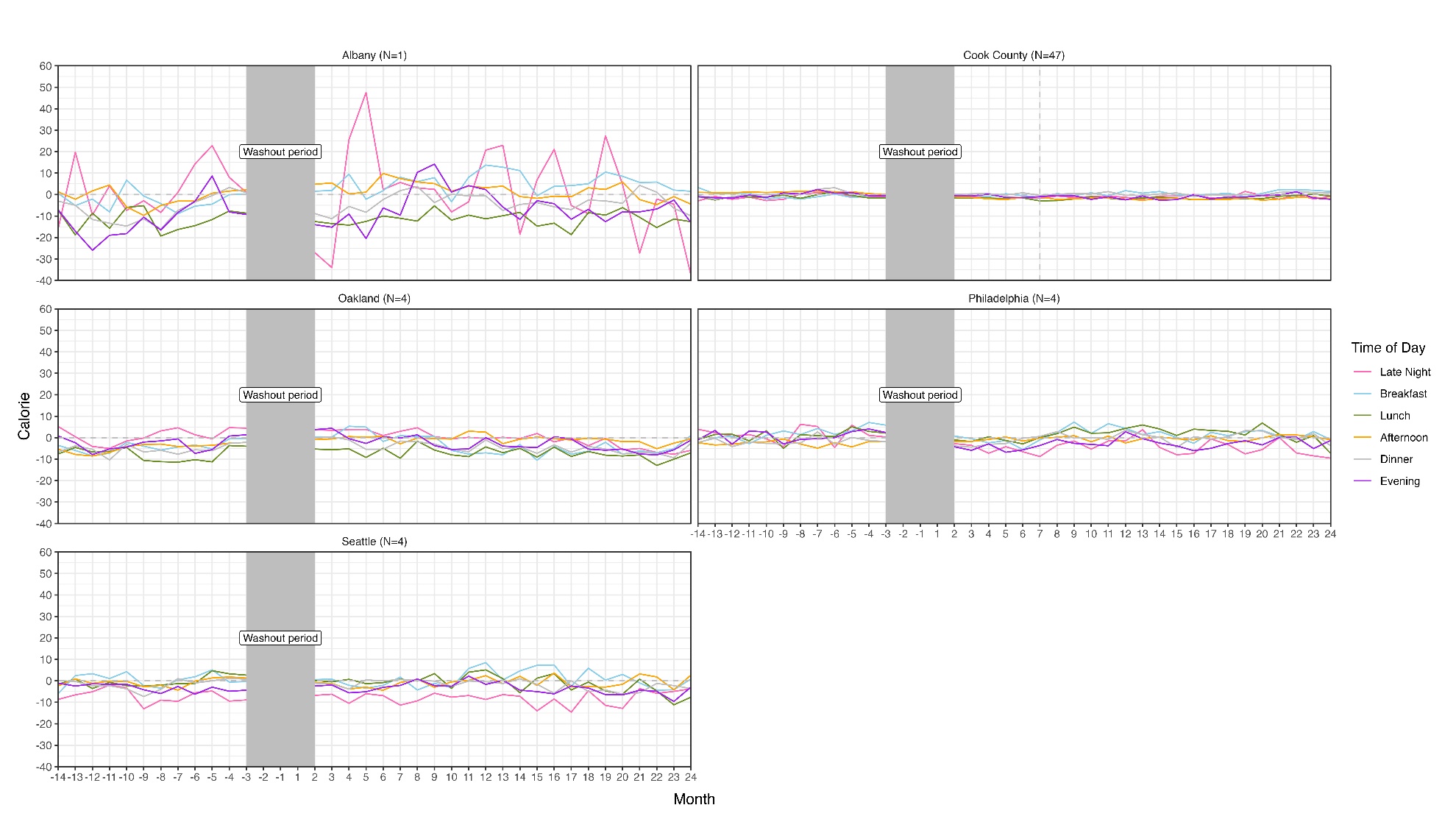


^a^Unconditional on a transaction including a beverage item.

**Figure E.2.** Difference-in-differences model estimates of beverage calories purchased per transaction, combo meals, by location and time of day, unconditional^a^


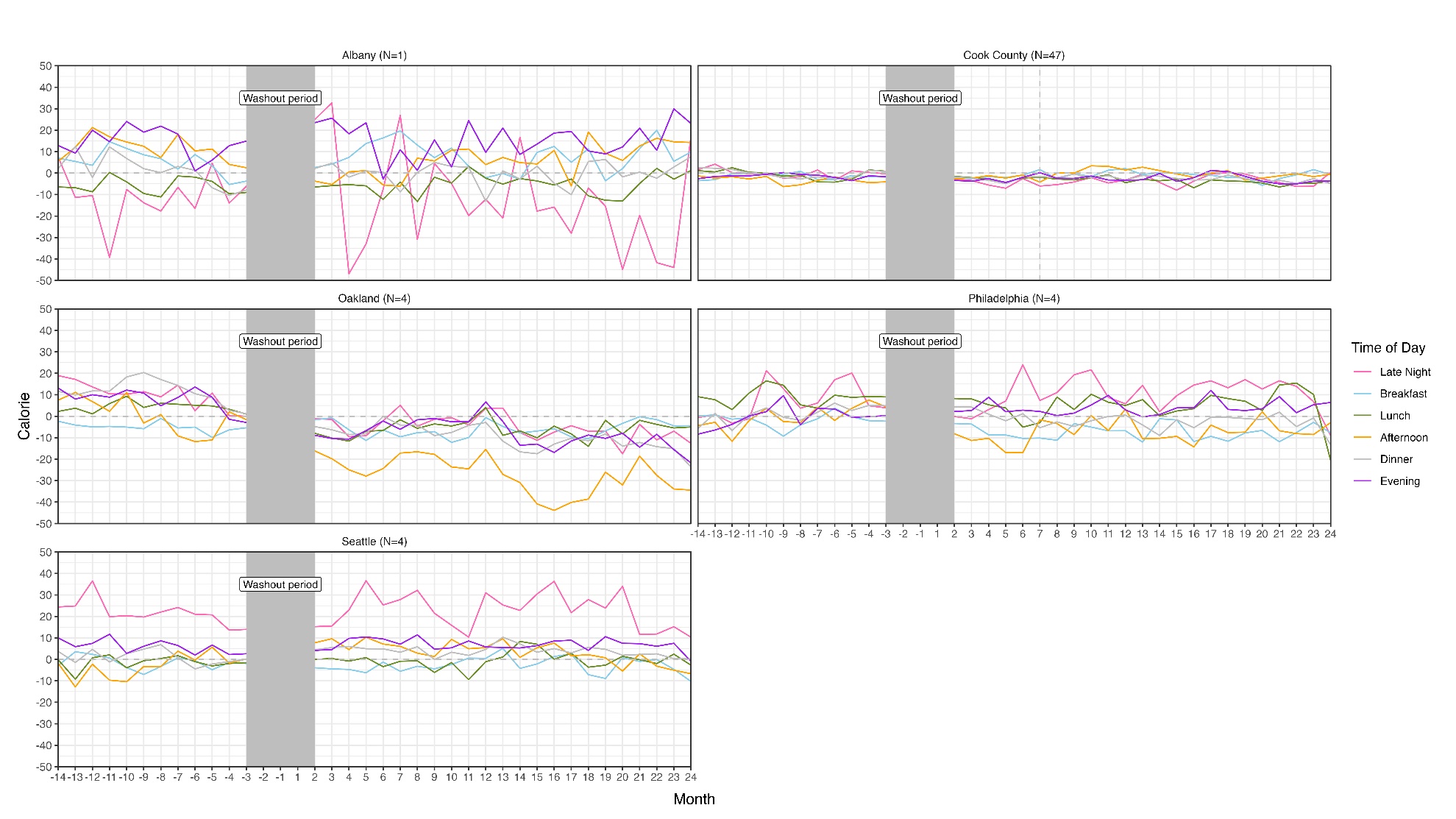


^a^Unconditional on a transaction including a beverage item.

**Figure F.1.** Difference-in-differences model estimates of beverage calories purchased from individual items per transaction after tax implementation, by months open after tax implementation, unconditional^a^


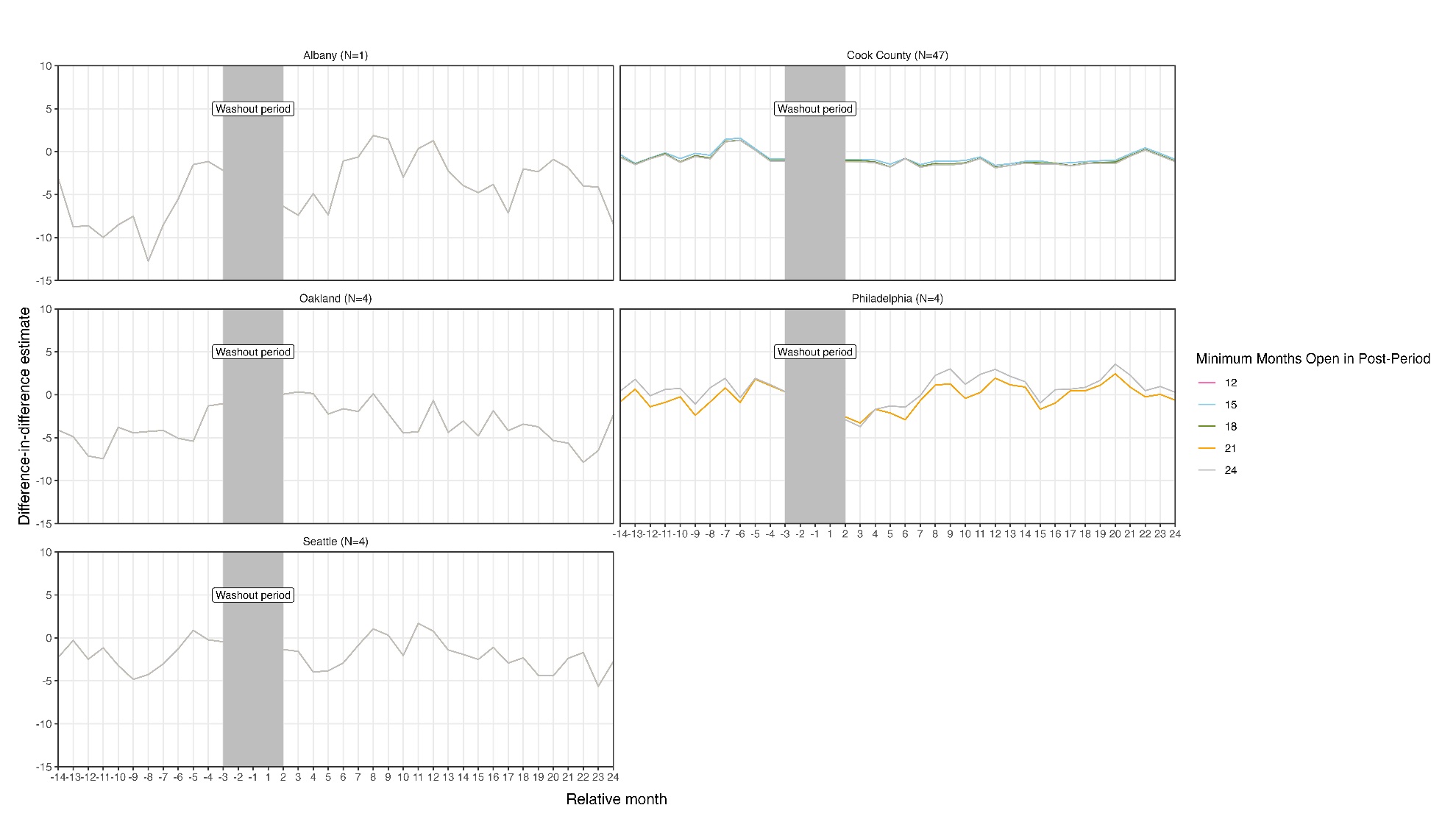
^a^Unconditional on a transaction including a beverage item.

**Figure F.2.** Difference-in-differences model estimates of beverage calories purchased from combo meals per transaction after tax implementation, by months open after soda tax implementation, unconditional^a^


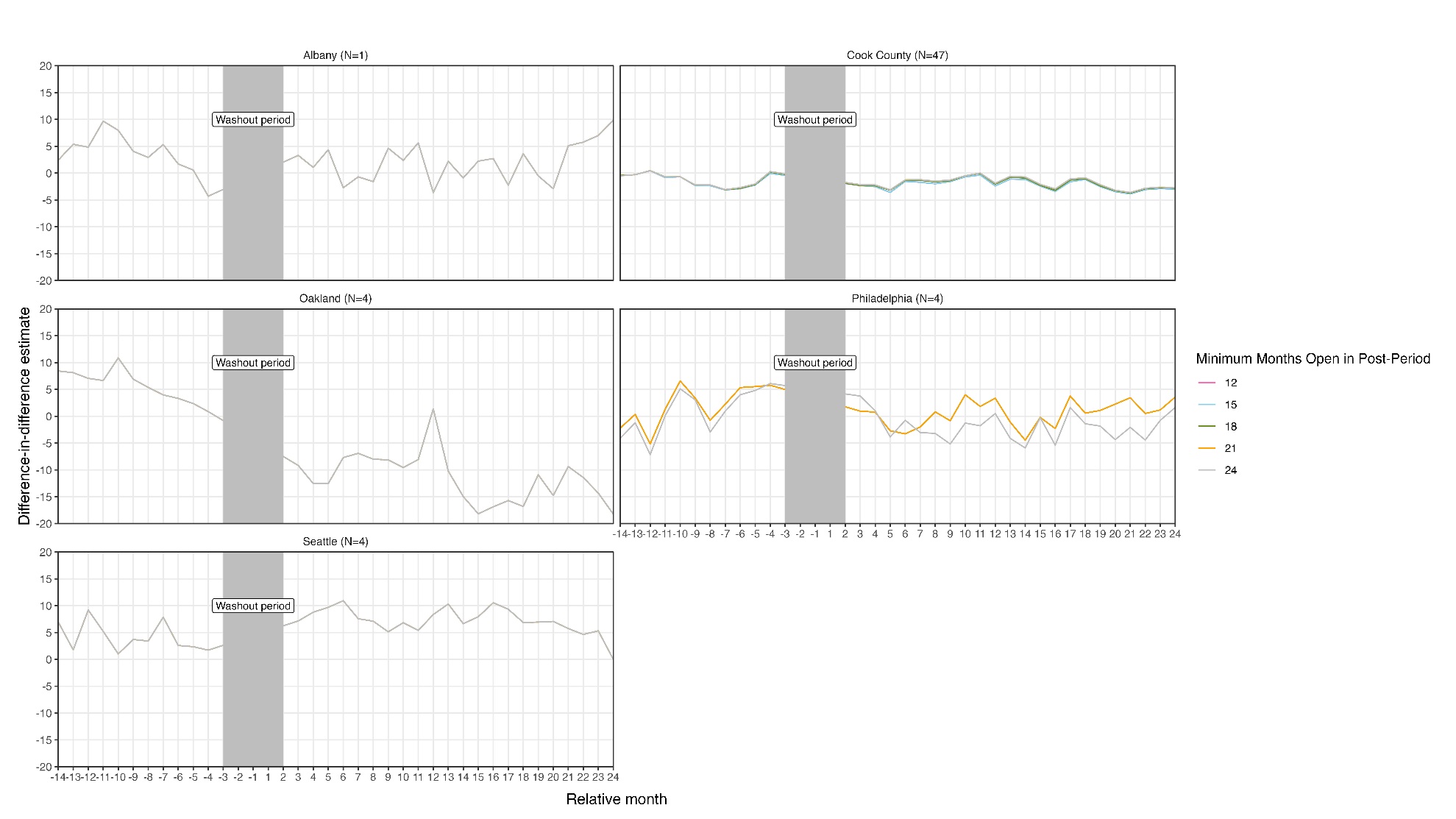


^a^Unconditional on a transaction including a beverage item.

**Figure G.1.** Difference-in-differences model estimates of beverage calories purchased per transaction after tax implementation, by restaurant, individual items, unconditional^a^


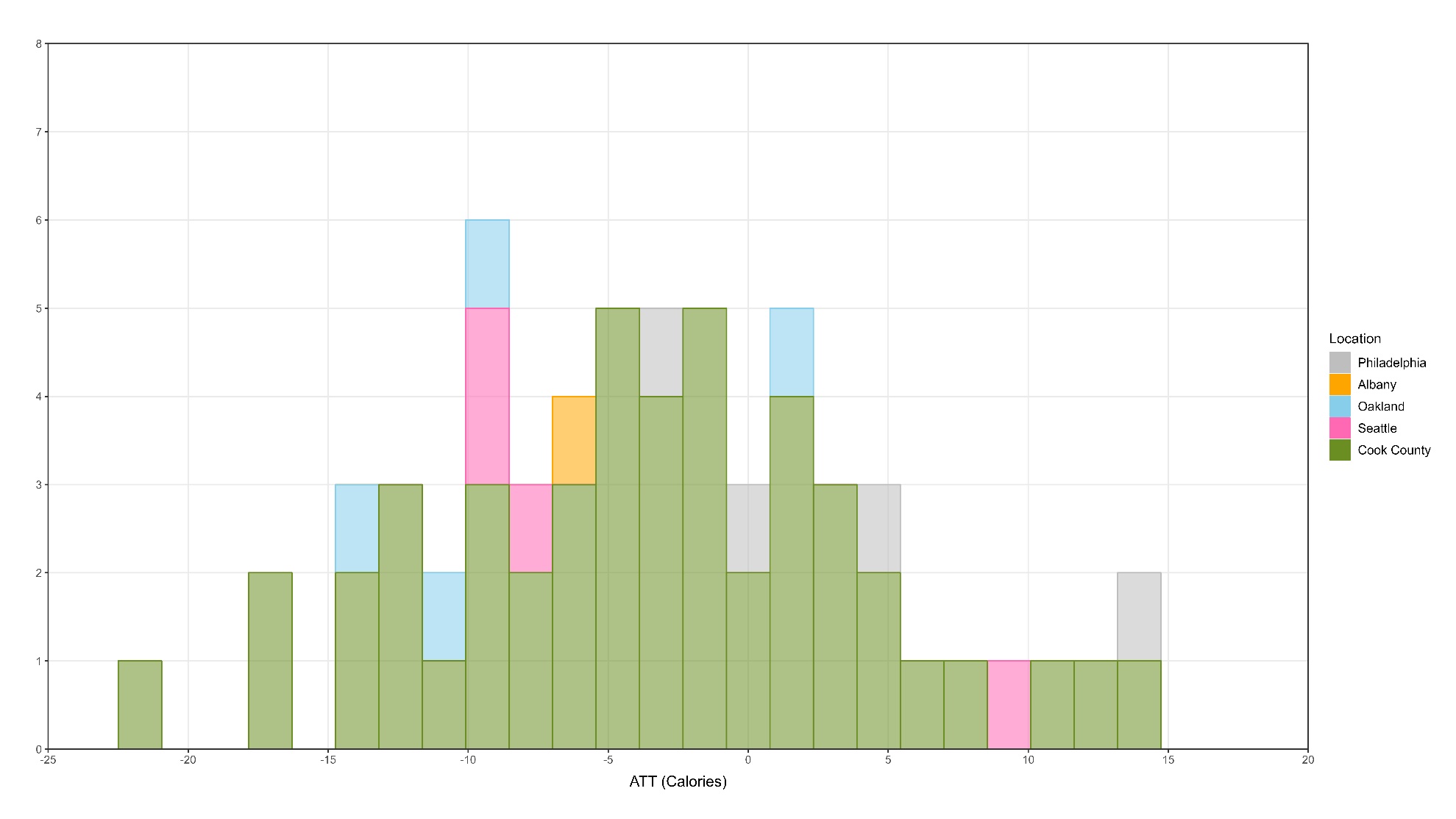
^a^Unconditional on a transaction including a beverage item.

**Figure G.2.** Difference-in-differences model estimates of beverage calories purchased per transaction after tax implementation, by restaurant, combo meals, conditional, unconditional^a^


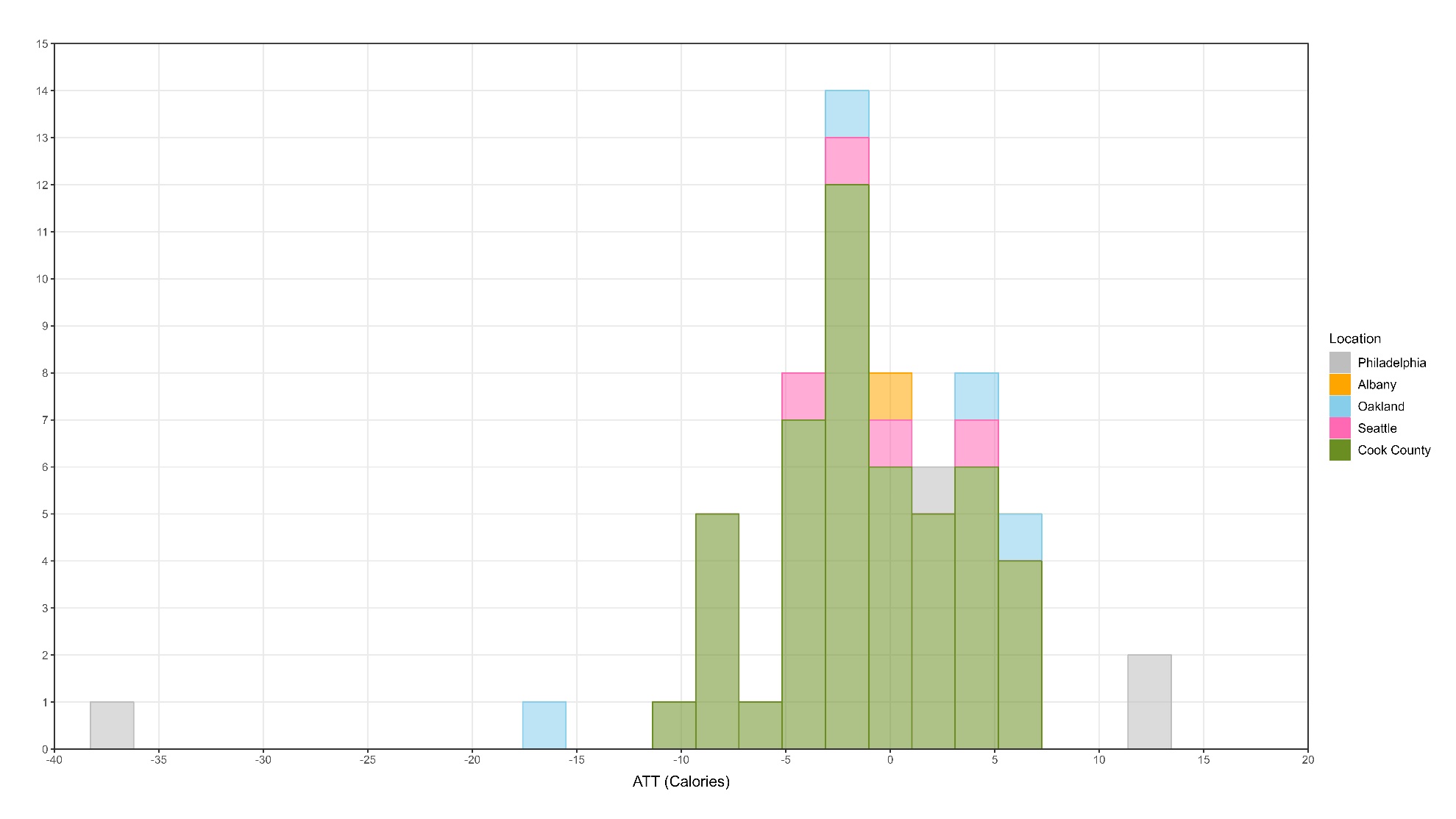


^a^Unconditional on a transaction including a beverage item.

**eMethods.** Supplementary description of matching procedures and statistical analyses

*Sample size of sugary drink tax and comparison restaurants*


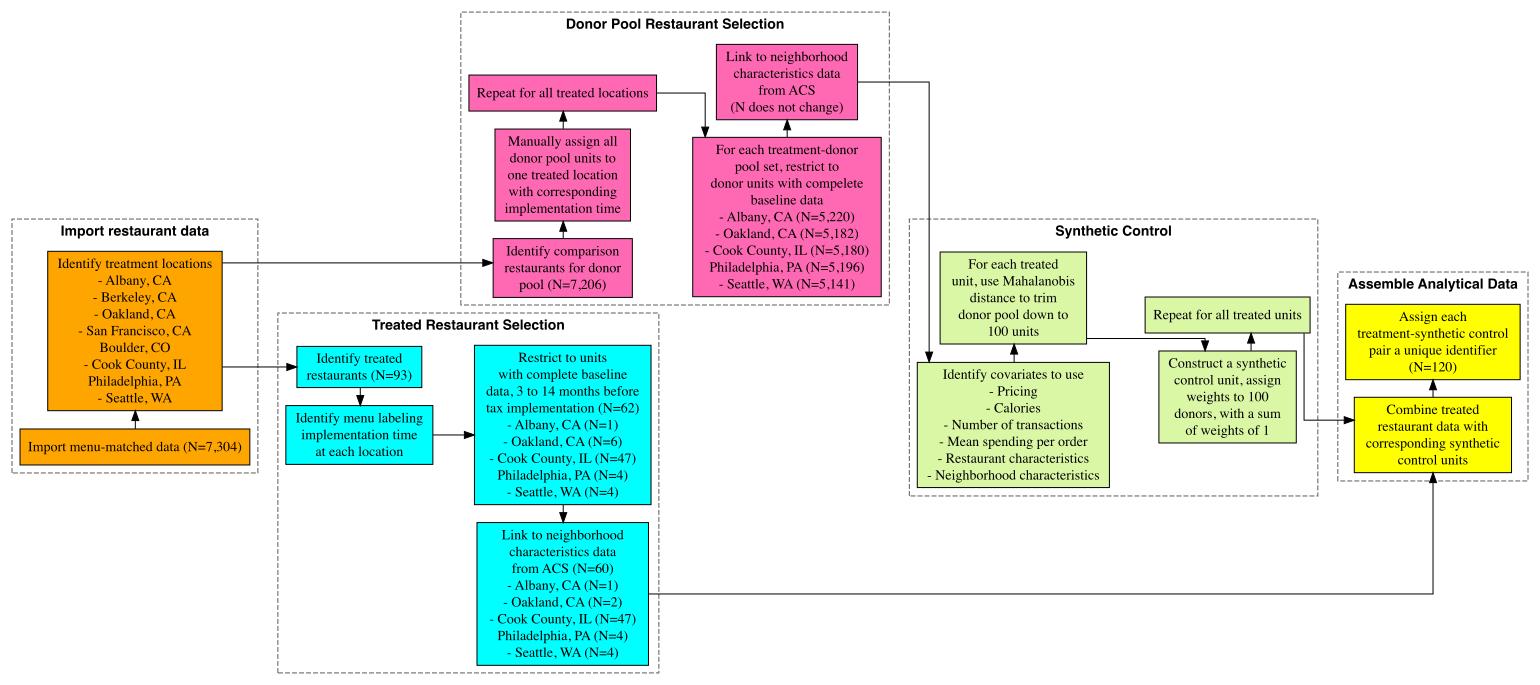


NOTE: A comparison restaurant can serve as a comparison to multiple treated restaurants. Each synthetic control unit is comprised of 100 non-zero-weighted comparison restaurants, with weights summing to 1.

*Code related to the matching procedures and the statistical analyses, including annotations* <file:///C:/Users/rummop01/AppData/Local/Microsoft/Windows/INetCache/Content.Outlook/E4MTJOF7/codeshare.html>

*Methods for matching of menu items with nutrition information in MenuStat*

Using the stringdist function in R, we calculated the Jaccard distance (i.e., degree of similarity in text strings) between items in our database and MenuStat and selected the most similar pair of items. To address ambiguity in non-exact matches (93%), five Research Assistants completed two rounds of manual item name matching. Each Research Assistant was assigned two lists of 1,000 items and instructed to confirm whether items were a match, not a match, or maybe a match. Each item was evaluated twice by two different Research Assistants independently, and inter-rater reliability was substantial (Cohen’s weighted Kappa=0.71). Prior to arbitration, we filtered the dataset to the top 95% of sales for item pairs classified as not a match or maybe a match. For item pairs classified as maybe a match, a third Research Assistant classified the items as a match or not a match. For all item pairs classified as not a match by at least two Research Assistants, a manual search of similar items on MenuStat and other internet sources was undertaken.
